# Measurement of the *W*-boson mass in pp collisions at $\sqrt{s}=7\, \hbox {TeV}$ with the ATLAS detector

**DOI:** 10.1140/epjc/s10052-017-5475-4

**Published:** 2018-02-06

**Authors:** M. Aaboud, G. Aad, B. Abbott, J. Abdallah, O. Abdinov, B. Abeloos, S. H. Abidi, O. S. AbouZeid, N. L. Abraham, H. Abramowicz, H. Abreu, R. Abreu, Y. Abulaiti, B. S. Acharya, S. Adachi, L. Adamczyk, D. L. Adams, J. Adelman, M. Adersberger, T. Adye, A. A. Affolder, T. Agatonovic-Jovin, C. Agheorghiesei, J. A. Aguilar-Saavedra, S. P. Ahlen, F. Ahmadov, G. Aielli, S. Akatsuka, H. Akerstedt, T. P. A. Åkesson, A. V. Akimov, G. L. Alberghi, J. Albert, M. J. Alconada Verzini, M. Aleksa, I. N. Aleksandrov, C. Alexa, G. Alexander, T. Alexopoulos, M. Alhroob, B. Ali, M. Aliev, G. Alimonti, J. Alison, S. P. Alkire, B. M. M. Allbrooke, B. W. Allen, P. P. Allport, A. Aloisio, A. Alonso, F. Alonso, C. Alpigiani, A. A. Alshehri, M. Alstaty, B. Alvarez Gonzalez, D. Álvarez Piqueras, M. G. Alviggi, B. T. Amadio, Y. Amaral Coutinho, C. Amelung, D. Amidei, S. P. Amor Dos Santos, A. Amorim, S. Amoroso, G. Amundsen, C. Anastopoulos, L. S. Ancu, N. Andari, T. Andeen, C. F. Anders, J. K. Anders, K. J. Anderson, A. Andreazza, V. Andrei, S. Angelidakis, I. Angelozzi, A. Angerami, F. Anghinolfi, A. V. Anisenkov, N. Anjos, A. Annovi, C. Antel, M. Antonelli, A. Antonov, D. J. Antrim, F. Anulli, M. Aoki, L. Aperio Bella, G. Arabidze, Y. Arai, J. P. Araque, V. Araujo Ferraz, A. T. H. Arce, R. E. Ardell, F. A. Arduh, J-F. Arguin, S. Argyropoulos, M. Arik, A. J. Armbruster, L. J. Armitage, O. Arnaez, H. Arnold, M. Arratia, O. Arslan, A. Artamonov, G. Artoni, S. Artz, S. Asai, N. Asbah, A. Ashkenazi, L. Asquith, K. Assamagan, R. Astalos, M. Atkinson, N. B. Atlay, K. Augsten, G. Avolio, B. Axen, M. K. Ayoub, G. Azuelos, A. E. Baas, M. J. Baca, H. Bachacou, K. Bachas, M. Backes, M. Backhaus, P. Bagiacchi, P. Bagnaia, J. T. Baines, M. Bajic, O. K. Baker, E. M. Baldin, P. Balek, T. Balestri, F. Balli, W. K. Balunas, E. Banas, Sw. Banerjee, A. A. E. Bannoura, L. Barak, E. L. Barberio, D. Barberis, M. Barbero, T. Barillari, M-S. Barisits, T. Barklow, N. Barlow, S. L. Barnes, B. M. Barnett, R. M. Barnett, Z. Barnovska-Blenessy, A. Baroncelli, G. Barone, A. J. Barr, L. Barranco Navarro, F. Barreiro, J. Barreiro Guimarães da Costa, R. Bartoldus, A. E. Barton, P. Bartos, A. Basalaev, A. Bassalat, R. L. Bates, S. J. Batista, J. R. Batley, M. Battaglia, M. Bauce, F. Bauer, H. S. Bawa, J. B. Beacham, M. D. Beattie, T. Beau, P. H. Beauchemin, P. Bechtle, H. P. Beck, K. Becker, M. Becker, M. Beckingham, C. Becot, A. J. Beddall, A. Beddall, V. A. Bednyakov, M. Bedognetti, C. P. Bee, T. A. Beermann, M. Begalli, M. Begel, J. K. Behr, A. S. Bell, G. Bella, L. Bellagamba, A. Bellerive, M. Bellomo, K. Belotskiy, O. Beltramello, N. L. Belyaev, O. Benary, D. Benchekroun, M. Bender, K. Bendtz, N. Benekos, Y. Benhammou, E. Benhar Noccioli, J. Benitez, D. P. Benjamin, M. Benoit, J. R. Bensinger, S. Bentvelsen, L. Beresford, M. Beretta, D. Berge, E. Bergeaas Kuutmann, N. Berger, J. Beringer, S. Berlendis, N. R. Bernard, G. Bernardi, C. Bernius, F. U. Bernlochner, T. Berry, P. Berta, C. Bertella, G. Bertoli, F. Bertolucci, I. A. Bertram, C. Bertsche, D. Bertsche, G. J. Besjes, O. Bessidskaia Bylund, M. Bessner, N. Besson, C. Betancourt, A. Bethani, S. Bethke, A. J. Bevan, R. M. Bianchi, O. Biebel, D. Biedermann, M. Bianco, R. Bielski, N. V. Biesuz, M. Biglietti, J. Bilbao De Mendizabal, T. R. V. Billoud, H. Bilokon, M. Bindi, A. Bingul, C. Bini, S. Biondi, T. Bisanz, C. Bittrich, D. M. Bjergaard, C. W. Black, J. E. Black, K. M. Black, D. Blackburn, R. E. Blair, J.-B. Blanchard, T. Blazek, I. Bloch, C. Blocker, A. Blue, W. Blum, U. Blumenschein, S. Blunier, G. J. Bobbink, V. S. Bobrovnikov, S. S. Bocchetta, A. Bocci, C. Bock, M. Boehler, D. Boerner, D. Bogavac, A. G. Bogdanchikov, C. Bohm, V. Boisvert, P. Bokan, T. Bold, A. S. Boldyrev, M. Bomben, M. Bona, M. Boonekamp, A. Borisov, G. Borissov, J. Bortfeldt, D. Bortoletto, V. Bortolotto, K. Bos, D. Boscherini, M. Bosman, J. D. Bossio Sola, J. Boudreau, J. Bouffard, E. V. Bouhova-Thacker, D. Boumediene, C. Bourdarios, S. K. Boutle, A. Boveia, J. Boyd, I. R. Boyko, J. Bracinik, A. Brandt, G. Brandt, O. Brandt, U. Bratzler, B. Brau, J. E. Brau, W. D. Breaden Madden, K. Brendlinger, A. J. Brennan, L. Brenner, R. Brenner, S. Bressler, D. L. Briglin, T. M. Bristow, D. Britton, D. Britzger, F. M. Brochu, I. Brock, R. Brock, G. Brooijmans, T. Brooks, W. K. Brooks, J. Brosamer, E. Brost, J. H Broughton, P. A. Bruckman de Renstrom, D. Bruncko, A. Bruni, G. Bruni, L. S. Bruni, B H Brunt, M. Bruschi, N. Bruscino, P. Bryant, L. Bryngemark, T. Buanes, Q. Buat, P. Buchholz, A. G. Buckley, I. A. Budagov, F. Buehrer, M. K. Bugge, O. Bulekov, D. Bullock, H. Burckhart, S. Burdin, C. D. Burgard, A. M. Burger, B. Burghgrave, K. Burka, S. Burke, I. Burmeister, J. T. P. Burr, E. Busato, D. Büscher, V. Büscher, P. Bussey, J. M. Butler, C. M. Buttar, J. M. Butterworth, P. Butti, W. Buttinger, A. Buzatu, A. R. Buzykaev, S. Cabrera Urbán, D. Caforio, V. M. Cairo, O. Cakir, N. Calace, P. Calafiura, A. Calandri, G. Calderini, P. Calfayan, G. Callea, L. P. Caloba, S. Calvente Lopez, D. Calvet, S. Calvet, T. P. Calvet, R. Camacho Toro, S. Camarda, P. Camarri, D. Cameron, R. Caminal Armadans, C. Camincher, S. Campana, M. Campanelli, A. Camplani, A. Campoverde, V. Canale, M. Cano Bret, J. Cantero, T. Cao, M. D. M. Capeans Garrido, I. Caprini, M. Caprini, M. Capua, R. M. Carbone, R. Cardarelli, F. Cardillo, I. Carli, T. Carli, G. Carlino, B. T. Carlson, L. Carminati, R. M. D. Carney, S. Caron, E. Carquin, G. D. Carrillo-Montoya, J. Carvalho, D. Casadei, M. P. Casado, M. Casolino, D. W. Casper, R. Castelijn, A. Castelli, V. Castillo Gimenez, N. F. Castro, A. Catinaccio, J. R. Catmore, A. Cattai, J. Caudron, V. Cavaliere, E. Cavallaro, D. Cavalli, M. Cavalli-Sforza, V. Cavasinni, E. Celebi, F. Ceradini, L. Cerda Alberich, A. S. Cerqueira, A. Cerri, L. Cerrito, F. Cerutti, A. Cervelli, S. A. Cetin, A. Chafaq, D. Chakraborty, S. K. Chan, W. S. Chan, Y. L. Chan, P. Chang, J. D. Chapman, D. G. Charlton, A. Chatterjee, C. C. Chau, C. A. Chavez Barajas, S. Che, S. Cheatham, A. Chegwidden, S. Chekanov, S. V. Chekulaev, G. A. Chelkov, M. A. Chelstowska, C. Chen, H. Chen, S. Chen, S. Chen, X. Chen, Y. Chen, H. C. Cheng, H. J. Cheng, Y. Cheng, A. Cheplakov, E. Cheremushkina, R. Cherkaoui El Moursli, V. Chernyatin, E. Cheu, L. Chevalier, V. Chiarella, G. Chiarelli, G. Chiodini, A. S. Chisholm, A. Chitan, Y. H. Chiu, M. V. Chizhov, K. Choi, A. R. Chomont, S. Chouridou, B. K. B. Chow, V. Christodoulou, D. Chromek-Burckhart, M. C. Chu, J. Chudoba, A. J. Chuinard, J. J. Chwastowski, L. Chytka, A. K. Ciftci, D. Cinca, V. Cindro, I. A. Cioara, C. Ciocca, A. Ciocio, F. Cirotto, Z. H. Citron, M. Citterio, M. Ciubancan, A. Clark, B. L. Clark, M. R. Clark, P. J. Clark, R. N. Clarke, C. Clement, Y. Coadou, M. Cobal, A. Coccaro, J. Cochran, L. Colasurdo, B. Cole, A. P. Colijn, J. Collot, T. Colombo, P. Conde Muiño, E. Coniavitis, S. H. Connell, I. A. Connelly, V. Consorti, S. Constantinescu, G. Conti, F. Conventi, M. Cooke, B. D. Cooper, A. M. Cooper-Sarkar, F. Cormier, K. J. R. Cormier, T. Cornelissen, M. Corradi, F. Corriveau, A. Cortes-Gonzalez, G. Cortiana, G. Costa, M. J. Costa, D. Costanzo, G. Cottin, G. Cowan, B. E. Cox, K. Cranmer, S. J. Crawley, R. A. Creager, G. Cree, S. Crépé-Renaudin, F. Crescioli, W. A. Cribbs, M. Crispin Ortuzar, M. Cristinziani, V. Croft, G. Crosetti, A. Cueto, T. Cuhadar Donszelmann, J. Cummings, M. Curatolo, J. Cúth, H. Czirr, P. Czodrowski, G. D’amen, S. D’Auria, M. D’Onofrio, M. J. Da Cunha Sargedas De Sousa, C. Da Via, W. Dabrowski, T. Dado, T. Dai, O. Dale, F. Dallaire, C. Dallapiccola, M. Dam, J. R. Dandoy, N. P. Dang, A. C. Daniells, N. S. Dann, M. Danninger, M. Dano Hoffmann, V. Dao, G. Darbo, S. Darmora, J. Dassoulas, A. Dattagupta, T. Daubney, W. Davey, C. David, T. Davidek, M. Davies, P. Davison, E. Dawe, I. Dawson, K. De, R. de Asmundis, A. De Benedetti, S. De Castro, S. De Cecco, N. De Groot, P. de Jong, H. De la Torre, F. De Lorenzi, A. De Maria, D. De Pedis, A. De Salvo, U. De Sanctis, A. De Santo, K. De Vasconcelos Corga, J. B. De Vivie De Regie, W. J. Dearnaley, R. Debbe, C. Debenedetti, D. V. Dedovich, N. Dehghanian, I. Deigaard, M. Del Gaudio, J. Del Peso, T. Del Prete, D. Delgove, F. Deliot, C. M. Delitzsch, A. Dell’Acqua, L. Dell’Asta, M. Dell’Orso, M. Della Pietra, D. della Volpe, M. Delmastro, P. A. Delsart, D. A. DeMarco, S. Demers, M. Demichev, A. Demilly, S. P. Denisov, D. Denysiuk, D. Derendarz, J. E. Derkaoui, F. Derue, P. Dervan, K. Desch, C. Deterre, K. Dette, P. O. Deviveiros, A. Dewhurst, S. Dhaliwal, A. Di Ciaccio, L. Di Ciaccio, W. K. Di Clemente, C. Di Donato, A. Di Girolamo, B. Di Girolamo, B. Di Micco, R. Di Nardo, K. F. Di Petrillo, A. Di Simone, R. Di Sipio, D. Di Valentino, C. Diaconu, M. Diamond, F. A. Dias, M. A. Diaz, E. B. Diehl, J. Dietrich, S. Díez Cornell, A. Dimitrievska, J. Dingfelder, P. Dita, S. Dita, F. Dittus, F. Djama, T. Djobava, J. I. Djuvsland, M. A. B. do Vale, D. Dobos, M. Dobre, C. Doglioni, J. Dolejsi, Z. Dolezal, M. Donadelli, S. Donati, P. Dondero, J. Donini, J. Dopke, A. Doria, M. T. Dova, A. T. Doyle, E. Drechsler, M. Dris, Y. Du, J. Duarte-Campderros, E. Duchovni, G. Duckeck, O. A. Ducu, D. Duda, A. Dudarev, A. Chr. Dudder, E. M. Duffield, L. Duflot, M. Dührssen, M. Dumancic, A. E. Dumitriu, A. K. Duncan, M. Dunford, H. Duran Yildiz, M. Düren, A. Durglishvili, D. Duschinger, B. Dutta, M. Dyndal, C. Eckardt, K. M. Ecker, R. C. Edgar, T. Eifert, G. Eigen, K. Einsweiler, T. Ekelof, M. El Kacimi, V. Ellajosyula, M. Ellert, S. Elles, F. Ellinghaus, A. A. Elliot, N. Ellis, J. Elmsheuser, M. Elsing, D. Emeliyanov, Y. Enari, O. C. Endner, J. S. Ennis, J. Erdmann, A. Ereditato, G. Ernis, M. Ernst, S. Errede, E. Ertel, M. Escalier, H. Esch, C. Escobar, B. Esposito, A. I. Etienvre, E. Etzion, H. Evans, A. Ezhilov, F. Fabbri, L. Fabbri, G. Facini, R. M. Fakhrutdinov, S. Falciano, R. J. Falla, J. Faltova, Y. Fang, M. Fanti, A. Farbin, A. Farilla, C. Farina, E. M. Farina, T. Farooque, S. Farrell, S. M. Farrington, P. Farthouat, F. Fassi, P. Fassnacht, D. Fassouliotis, M. Faucci Giannelli, A. Favareto, W. J. Fawcett, L. Fayard, O. L. Fedin, W. Fedorko, S. Feigl, L. Feligioni, C. Feng, E. J. Feng, H. Feng, A. B. Fenyuk, L. Feremenga, P. Fernandez Martinez, S. Fernandez Perez, J. Ferrando, A. Ferrari, P. Ferrari, R. Ferrari, D. E. Ferreira de Lima, A. Ferrer, D. Ferrere, C. Ferretti, F. Fiedler, A. Filipčič, M. Filipuzzi, F. Filthaut, M. Fincke-Keeler, K. D. Finelli, M. C. N. Fiolhais, L. Fiorini, A. Fischer, C. Fischer, J. Fischer, W. C. Fisher, N. Flaschel, I. Fleck, P. Fleischmann, R. R. M. Fletcher, T. Flick, B. M. Flierl, L. R. Flores Castillo, M. J. Flowerdew, G. T. Forcolin, A. Formica, A. Forti, A. G. Foster, D. Fournier, H. Fox, S. Fracchia, P. Francavilla, M. Franchini, D. Francis, L. Franconi, M. Franklin, M. Frate, M. Fraternali, D. Freeborn, S. M. Fressard-Batraneanu, B. Freund, D. Froidevaux, J. A. Frost, C. Fukunaga, E. Fullana Torregrosa, T. Fusayasu, J. Fuster, C. Gabaldon, O. Gabizon, A. Gabrielli, A. Gabrielli, G. P. Gach, S. Gadatsch, S. Gadomski, G. Gagliardi, L. G. Gagnon, P. Gagnon, C. Galea, B. Galhardo, E. J. Gallas, B. J. Gallop, P. Gallus, G. Galster, K. K. Gan, S. Ganguly, J. Gao, Y. Gao, Y. S. Gao, F. M. Garay Walls, C. García, J. E. García Navarro, M. Garcia-Sciveres, R. W. Gardner, N. Garelli, V. Garonne, A. Gascon Bravo, K. Gasnikova, C. Gatti, A. Gaudiello, G. Gaudio, I. L. Gavrilenko, C. Gay, G. Gaycken, E. N. Gazis, C. N. P. Gee, M. Geisen, M. P. Geisler, K. Gellerstedt, C. Gemme, M. H. Genest, C. Geng, S. Gentile, C. Gentsos, S. George, D. Gerbaudo, A. Gershon, S. Ghasemi, M. Ghneimat, B. Giacobbe, S. Giagu, P. Giannetti, S. M. Gibson, M. Gignac, M. Gilchriese, D. Gillberg, G. Gilles, D. M. Gingrich, N. Giokaris, M. P. Giordani, F. M. Giorgi, P. F. Giraud, P. Giromini, D. Giugni, F. Giuli, C. Giuliani, M. Giulini, B. K. Gjelsten, S. Gkaitatzis, I. Gkialas, E. L. Gkougkousis, L. K. Gladilin, C. Glasman, J. Glatzer, P. C. F. Glaysher, A. Glazov, M. Goblirsch-Kolb, J. Godlewski, S. Goldfarb, T. Golling, D. Golubkov, A. Gomes, R. Gonçalo, R. Goncalves Gama, J. Goncalves Pinto Firmino Da Costa, G. Gonella, L. Gonella, A. Gongadze, S. González de la Hoz, S. Gonzalez-Sevilla, L. Goossens, P. A. Gorbounov, H. A. Gordon, I. Gorelov, B. Gorini, E. Gorini, A. Gorišek, A. T. Goshaw, C. Gössling, M. I. Gostkin, C. R. Goudet, D. Goujdami, A. G. Goussiou, N. Govender, E. Gozani, L. Graber, I. Grabowska-Bold, P. O. J. Gradin, J. Gramling, E. Gramstad, S. Grancagnolo, V. Gratchev, P. M. Gravila, H. M. Gray, Z. D. Greenwood, C. Grefe, K. Gregersen, I. M. Gregor, P. Grenier, K. Grevtsov, J. Griffiths, A. A. Grillo, K. Grimm, S. Grinstein, Ph. Gris, J.-F. Grivaz, S. Groh, E. Gross, J. Grosse-Knetter, G. C. Grossi, Z. J. Grout, L. Guan, W. Guan, J. Guenther, F. Guescini, D. Guest, O. Gueta, B. Gui, E. Guido, T. Guillemin, S. Guindon, U. Gul, C. Gumpert, J. Guo, W. Guo, Y. Guo, R. Gupta, S. Gupta, G. Gustavino, P. Gutierrez, N. G. Gutierrez Ortiz, C. Gutschow, C. Guyot, M. P. Guzik, C. Gwenlan, C. B. Gwilliam, A. Haas, C. Haber, H. K. Hadavand, A. Hadef, S. Hageböck, M. Hagihara, H. Hakobyan, M. Haleem, J. Haley, G. Halladjian, G. D. Hallewell, K. Hamacher, P. Hamal, K. Hamano, A. Hamilton, G. N. Hamity, P. G. Hamnett, L. Han, S. Han, K. Hanagaki, K. Hanawa, M. Hance, B. Haney, P. Hanke, R. Hanna, J. B. Hansen, J. D. Hansen, M. C. Hansen, P. H. Hansen, K. Hara, A. S. Hard, T. Harenberg, F. Hariri, S. Harkusha, R. D. Harrington, P. F. Harrison, F. Hartjes, N. M. Hartmann, M. Hasegawa, Y. Hasegawa, A. Hasib, S. Hassani, S. Haug, R. Hauser, L. Hauswald, L. B. Havener, M. Havranek, C. M. Hawkes, R. J. Hawkings, D. Hayakawa, D. Hayden, C. P. Hays, J. M. Hays, H. S. Hayward, S. J. Haywood, S. J. Head, T. Heck, V. Hedberg, L. Heelan, K. K. Heidegger, S. Heim, T. Heim, B. Heinemann, J. J. Heinrich, L. Heinrich, C. Heinz, J. Hejbal, L. Helary, A. Held, S. Hellman, C. Helsens, J. Henderson, R. C. W. Henderson, Y. Heng, S. Henkelmann, A. M. Henriques Correia, S. Henrot-Versille, G. H. Herbert, H. Herde, V. Herget, Y. Hernández Jiménez, G. Herten, R. Hertenberger, L. Hervas, T. C. Herwig, G. G. Hesketh, N. P. Hessey, J. W. Hetherly, S. Higashino, E. Higón-Rodriguez, E. Hill, J. C. Hill, K. H. Hiller, S. J. Hillier, I. Hinchliffe, M. Hirose, D. Hirschbuehl, B. Hiti, O. Hladik, X. Hoad, J. Hobbs, N. Hod, M. C. Hodgkinson, P. Hodgson, A. Hoecker, M. R. Hoeferkamp, F. Hoenig, D. Hohn, T. R. Holmes, M. Homann, S. Honda, T. Honda, T. M. Hong, B. H. Hooberman, W. H. Hopkins, Y. Horii, A. J. Horton, J-Y. Hostachy, S. Hou, A. Hoummada, J. Howarth, J. Hoya, M. Hrabovsky, I. Hristova, J. Hrivnac, T. Hryn’ova, A. Hrynevich, P. J. Hsu, S.-C. Hsu, Q. Hu, S. Hu, Y. Huang, Z. Hubacek, F. Hubaut, F. Huegging, T. B. Huffman, E. W. Hughes, G. Hughes, M. Huhtinen, P. Huo, N. Huseynov, J. Huston, J. Huth, G. Iacobucci, G. Iakovidis, I. Ibragimov, L. Iconomidou-Fayard, P. Iengo, O. Igonkina, T. Iizawa, Y. Ikegami, M. Ikeno, Y. Ilchenko, D. Iliadis, N. Ilic, G. Introzzi, P. Ioannou, M. Iodice, K. Iordanidou, V. Ippolito, N. Ishijima, M. Ishino, M. Ishitsuka, C. Issever, S. Istin, F. Ito, J. M. Iturbe Ponce, R. Iuppa, H. Iwasaki, J. M. Izen, V. Izzo, S. Jabbar, P. Jackson, V. Jain, K. B. Jakobi, K. Jakobs, S. Jakobsen, T. Jakoubek, D. O. Jamin, D. K. Jana, R. Jansky, J. Janssen, M. Janus, P. A. Janus, G. Jarlskog, N. Javadov, T. Javůrek, M. Javurkova, F. Jeanneau, L. Jeanty, J. Jejelava, A. Jelinskas, P. Jenni, C. Jeske, S. Jézéquel, H. Ji, J. Jia, H. Jiang, Y. Jiang, Z. Jiang, S. Jiggins, J. Jimenez Pena, S. Jin, A. Jinaru, O. Jinnouchi, H. Jivan, P. Johansson, K. A. Johns, C. A. Johnson, W. J. Johnson, K. Jon-And, R. W. L. Jones, S. Jones, T. J. Jones, J. Jongmanns, P. M. Jorge, J. Jovicevic, X. Ju, A. Juste Rozas, M. K. Köhler, A. Kaczmarska, M. Kado, H. Kagan, M. Kagan, S. J. Kahn, T. Kaji, E. Kajomovitz, C. W. Kalderon, A. Kaluza, S. Kama, A. Kamenshchikov, N. Kanaya, S. Kaneti, L. Kanjir, V. A. Kantserov, J. Kanzaki, B. Kaplan, L. S. Kaplan, D. Kar, K. Karakostas, N. Karastathis, M. J. Kareem, E. Karentzos, M. Karnevskiy, S. N. Karpov, Z. M. Karpova, K. Karthik, V. Kartvelishvili, A. N. Karyukhin, K. Kasahara, L. Kashif, R. D. Kass, A. Kastanas, Y. Kataoka, C. Kato, A. Katre, J. Katzy, K. Kawade, K. Kawagoe, T. Kawamoto, G. Kawamura, E. F. Kay, V. F. Kazanin, R. Keeler, R. Kehoe, J. S. Keller, J. J. Kempster, H. Keoshkerian, O. Kepka, B. P. Kerševan, S. Kersten, R. A. Keyes, M. Khader, F. Khalil-zada, A. Khanov, A. G. Kharlamov, T. Kharlamova, A. Khodinov, T. J. Khoo, V. Khovanskiy, E. Khramov, J. Khubua, S. Kido, C. R. Kilby, H. Y. Kim, S. H. Kim, Y. K. Kim, N. Kimura, O. M. Kind, B. T. King, D. Kirchmeier, J. Kirk, A. E. Kiryunin, T. Kishimoto, D. Kisielewska, K. Kiuchi, O. Kivernyk, E. Kladiva, T. Klapdor-Kleingrothaus, M. H. Klein, M. Klein, U. Klein, K. Kleinknecht, P. Klimek, A. Klimentov, R. Klingenberg, T. Klioutchnikova, E.-E. Kluge, P. Kluit, S. Kluth, J. Knapik, E. Kneringer, E. B. F. G. Knoops, A. Knue, A. Kobayashi, D. Kobayashi, T. Kobayashi, M. Kobel, M. Kocian, P. Kodys, T. Koffas, E. Koffeman, N. M. Köhler, T. Koi, M. Kolb, I. Koletsou, A. A. Komar, Y. Komori, T. Kondo, N. Kondrashova, K. Köneke, A. C. König, T. Kono, R. Konoplich, N. Konstantinidis, R. Kopeliansky, S. Koperny, A. K. Kopp, K. Korcyl, K. Kordas, A. Korn, A. A. Korol, I. Korolkov, E. V. Korolkova, O. Kortner, S. Kortner, T. Kosek, V. V. Kostyukhin, A. Kotwal, A. Koulouris, A. Kourkoumeli-Charalampidi, C. Kourkoumelis, V. Kouskoura, A. B. Kowalewska, R. Kowalewski, T. Z. Kowalski, C. Kozakai, W. Kozanecki, A. S. Kozhin, V. A. Kramarenko, G. Kramberger, D. Krasnopevtsev, M. W. Krasny, A. Krasznahorkay, D. Krauss, A. Kravchenko, J. A. Kremer, M. Kretz, J. Kretzschmar, K. Kreutzfeldt, P. Krieger, K. Krizka, K. Kroeninger, H. Kroha, J. Kroll, J. Kroseberg, J. Krstic, U. Kruchonak, H. Krüger, N. Krumnack, M. C. Kruse, M. Kruskal, T. Kubota, H. Kucuk, S. Kuday, J. T. Kuechler, S. Kuehn, A. Kugel, F. Kuger, T. Kuhl, V. Kukhtin, R. Kukla, Y. Kulchitsky, S. Kuleshov, Y. P. Kulinich, M. Kuna, T. Kunigo, A. Kupco, O. Kuprash, H. Kurashige, L. L. Kurchaninov, Y. A. Kurochkin, M. G. Kurth, V. Kus, E. S. Kuwertz, M. Kuze, J. Kvita, T. Kwan, D. Kyriazopoulos, A. La Rosa, J. L. La Rosa Navarro, L. La Rotonda, C. Lacasta, F. Lacava, J. Lacey, H. Lacker, D. Lacour, E. Ladygin, R. Lafaye, B. Laforge, T. Lagouri, S. Lai, S. Lammers, W. Lampl, E. Lançon, U. Landgraf, M. P. J. Landon, M. C. Lanfermann, V. S. Lang, J. C. Lange, A. J. Lankford, F. Lanni, K. Lantzsch, A. Lanza, A. Lapertosa, S. Laplace, J. F. Laporte, T. Lari, F. Lasagni Manghi, M. Lassnig, P. Laurelli, W. Lavrijsen, A. T. Law, P. Laycock, T. Lazovich, M. Lazzaroni, B. Le, O. Le Dortz, E. Le Guirriec, E. P. Le Quilleuc, M. LeBlanc, T. LeCompte, F. Ledroit-Guillon, C. A. Lee, S. C. Lee, L. Lee, B. Lefebvre, G. Lefebvre, M. Lefebvre, F. Legger, C. Leggett, A. Lehan, G. Lehmann Miotto, X. Lei, W. A. Leight, A. G. Leister, M. A. L. Leite, R. Leitner, D. Lellouch, B. Lemmer, K. J. C. Leney, T. Lenz, B. Lenzi, R. Leone, S. Leone, C. Leonidopoulos, G. Lerner, C. Leroy, A. A. J. Lesage, C. G. Lester, M. Levchenko, J. Levêque, D. Levin, L. J. Levinson, M. Levy, D. Lewis, M. Leyton, B. Li, C. Li, H. Li, L. Li, L. Li, Q. Li, S. Li, X. Li, Y. Li, Z. Liang, B. Liberti, A. Liblong, K. Lie, J. Liebal, W. Liebig, A. Limosani, S. C. Lin, T. H. Lin, B. E. Lindquist, A. E. Lionti, E. Lipeles, A. Lipniacka, M. Lisovyi, T. M. Liss, A. Lister, A. M. Litke, B. Liu, H. Liu, H. Liu, J. Liu, J. B. Liu, K. Liu, L. Liu, M. Liu, Y. L. Liu, Y. Liu, M. Livan, A. Lleres, J. Llorente Merino, S. L. Lloyd, C. Y. Lo, F. Lo Sterzo, E. M. Lobodzinska, P. Loch, F. K. Loebinger, K. M. Loew, A. Loginov, T. Lohse, K. Lohwasser, M. Lokajicek, B. A. Long, J. D. Long, R. E. Long, L. Longo, K. A. Looper, J. A. Lopez, D. Lopez Mateos, I. Lopez Paz, A. Lopez Solis, J. Lorenz, N. Lorenzo Martinez, M. Losada, P. J. Lösel, X. Lou, A. Lounis, J. Love, P. A. Love, H. Lu, N. Lu, Y. J. Lu, H. J. Lubatti, C. Luci, A. Lucotte, C. Luedtke, F. Luehring, W. Lukas, L. Luminari, O. Lundberg, B. Lund-Jensen, P. M. Luzi, D. Lynn, R. Lysak, E. Lytken, V. Lyubushkin, H. Ma, L. L. Ma, Y. Ma, G. Maccarrone, A. Macchiolo, C. M. Macdonald, B. Maček, J. Machado Miguens, D. Madaffari, R. Madar, H. J. Maddocks, W. F. Mader, A. Madsen, J. Maeda, S. Maeland, T. Maeno, A. Maevskiy, E. Magradze, J. Mahlstedt, C. Maiani, C. Maidantchik, A. A. Maier, T. Maier, A. Maio, S. Majewski, Y. Makida, N. Makovec, B. Malaescu, Pa. Malecki, V. P. Maleev, F. Malek, U. Mallik, D. Malon, C. Malone, S. Maltezos, S. Malyukov, J. Mamuzic, G. Mancini, L. Mandelli, I. Mandić, J. Maneira, L. Manhaes de Andrade Filho, J. Manjarres Ramos, A. Mann, A. Manousos, B. Mansoulie, J. D. Mansour, R. Mantifel, M. Mantoani, S. Manzoni, L. Mapelli, G. Marceca, L. March, G. Marchiori, M. Marcisovsky, M. Marjanovic, D. E. Marley, F. Marroquim, S. P. Marsden, Z. Marshall, M. U. F Martensson, S. Marti-Garcia, C. B. Martin, T. A. Martin, V. J. Martin, B. Martin dit Latour, M. Martinez, V. I. Martinez Outschoorn, S. Martin-Haugh, V. S. Martoiu, A. C. Martyniuk, A. Marzin, L. Masetti, T. Mashimo, R. Mashinistov, J. Masik, A. L. Maslennikov, L. Massa, P. Mastrandrea, A. Mastroberardino, T. Masubuchi, P. Mättig, J. Maurer, S. J. Maxfield, D. A. Maximov, R. Mazini, I. Maznas, S. M. Mazza, N. C. Mc Fadden, G. Mc Goldrick, S. P. Mc Kee, A. McCarn, R. L. McCarthy, T. G. McCarthy, L. I. McClymont, E. F. McDonald, J. A. Mcfayden, G. Mchedlidze, S. J. McMahon, P. C. McNamara, R. A. McPherson, S. Meehan, T. J. Megy, S. Mehlhase, A. Mehta, T. Meideck, K. Meier, C. Meineck, B. Meirose, D. Melini, B. R. Mellado Garcia, M. Melo, F. Meloni, S. B. Menary, L. Meng, X. T. Meng, A. Mengarelli, S. Menke, E. Meoni, S. Mergelmeyer, P. Mermod, L. Merola, C. Meroni, F. S. Merritt, A. Messina, J. Metcalfe, A. S. Mete, C. Meyer, J-P. Meyer, J. Meyer, H. Meyer Zu Theenhausen, F. Miano, R. P. Middleton, S. Miglioranzi, L. Mijović, G. Mikenberg, M. Mikestikova, M. Mikuž, M. Milesi, A. Milic, D. W. Miller, C. Mills, A. Milov, D. A. Milstead, A. A. Minaenko, Y. Minami, I. A. Minashvili, A. I. Mincer, B. Mindur, M. Mineev, Y. Minegishi, Y. Ming, L. M. Mir, K. P. Mistry, T. Mitani, J. Mitrevski, V. A. Mitsou, A. Miucci, P. S. Miyagawa, A. Mizukami, J. U. Mjörnmark, M. Mlynarikova, T. Moa, K. Mochizuki, P. Mogg, S. Mohapatra, S. Molander, R. Moles-Valls, R. Monden, M. C. Mondragon, K. Mönig, J. Monk, E. Monnier, A. Montalbano, J. Montejo Berlingen, F. Monticelli, S. Monzani, R. W. Moore, N. Morange, D. Moreno, M. Moreno Llácer, P. Morettini, S. Morgenstern, D. Mori, T. Mori, M. Morii, M. Morinaga, V. Morisbak, A. K. Morley, G. Mornacchi, J. D. Morris, L. Morvaj, P. Moschovakos, M. Mosidze, H. J. Moss, J. Moss, K. Motohashi, R. Mount, E. Mountricha, E. J. W. Moyse, S. Muanza, R. D. Mudd, F. Mueller, J. Mueller, R. S. P. Mueller, D. Muenstermann, P. Mullen, G. A. Mullier, F. J. Munoz Sanchez, W. J. Murray, H. Musheghyan, M. Muškinja, A. G. Myagkov, M. Myska, B. P. Nachman, O. Nackenhorst, K. Nagai, R. Nagai, K. Nagano, Y. Nagasaka, K. Nagata, M. Nagel, E. Nagy, A. M. Nairz, Y. Nakahama, K. Nakamura, T. Nakamura, I. Nakano, R. F. Naranjo Garcia, R. Narayan, D. I. Narrias Villar, I. Naryshkin, T. Naumann, G. Navarro, R. Nayyar, H. A. Neal, P. Yu. Nechaeva, T. J. Neep, A. Negri, M. Negrini, S. Nektarijevic, C. Nellist, A. Nelson, M. E. Nelson, S. Nemecek, P. Nemethy, A. A. Nepomuceno, M. Nessi, M. S. Neubauer, M. Neumann, R. M. Neves, P. Nevski, P. R. Newman, T. Y. Ng, T. Nguyen Manh, R. B. Nickerson, R. Nicolaidou, J. Nielsen, V. Nikolaenko, I. Nikolic-Audit, K. Nikolopoulos, J. K. Nilsen, P. Nilsson, Y. Ninomiya, A. Nisati, N. Nishu, R. Nisius, T. Nobe, Y. Noguchi, M. Nomachi, I. Nomidis, M. A. Nomura, T. Nooney, M. Nordberg, N. Norjoharuddeen, O. Novgorodova, S. Nowak, M. Nozaki, L. Nozka, K. Ntekas, E. Nurse, F. Nuti, D. C. O’Neil, A. A. O’Rourke, V. O’Shea, F. G. Oakham, H. Oberlack, T. Obermann, J. Ocariz, A. Ochi, I. Ochoa, J. P. Ochoa-Ricoux, S. Oda, S. Odaka, H. Ogren, A. Oh, S. H. Oh, C. C. Ohm, H. Ohman, H. Oide, H. Okawa, Y. Okumura, T. Okuyama, A. Olariu, L. F. Oleiro Seabra, S. A. Olivares Pino, D. Oliveira Damazio, A. Olszewski, J. Olszowska, A. Onofre, K. Onogi, P. U. E. Onyisi, M. J. Oreglia, Y. Oren, D. Orestano, N. Orlando, R. S. Orr, B. Osculati, R. Ospanov, G. Otero y Garzon, H. Otono, M. Ouchrif, F. Ould-Saada, A. Ouraou, K. P. Oussoren, Q. Ouyang, M. Owen, R. E. Owen, V. E. Ozcan, N. Ozturk, K. Pachal, A. Pacheco Pages, L. Pacheco Rodriguez, C. Padilla Aranda, S. Pagan Griso, M. Paganini, F. Paige, P. Pais, G. Palacino, S. Palazzo, S. Palestini, M. Palka, D. Pallin, E. St. Panagiotopoulou, I. Panagoulias, C. E. Pandini, J. G. Panduro Vazquez, P. Pani, S. Panitkin, D. Pantea, L. Paolozzi, Th. D. Papadopoulou, K. Papageorgiou, A. Paramonov, D. Paredes Hernandez, A. J. Parker, M. A. Parker, K. A. Parker, F. Parodi, J. A. Parsons, U. Parzefall, V. R. Pascuzzi, J. M. Pasner, E. Pasqualucci, S. Passaggio, Fr. Pastore, S. Pataraia, J. R. Pater, T. Pauly, J. Pearce, B. Pearson, L. E. Pedersen, S. Pedraza Lopez, R. Pedro, S. V. Peleganchuk, O. Penc, C. Peng, H. Peng, J. Penwell, B. S. Peralva, M. M. Perego, D. V. Perepelitsa, L. Perini, H. Pernegger, S. Perrella, R. Peschke, V. D. Peshekhonov, K. Peters, R. F. Y. Peters, B. A. Petersen, T. C. Petersen, E. Petit, A. Petridis, C. Petridou, P. Petroff, E. Petrolo, M. Petrov, F. Petrucci, N. E. Pettersson, A. Peyaud, R. Pezoa, P. W. Phillips, G. Piacquadio, E. Pianori, A. Picazio, E. Piccaro, M. A. Pickering, R. Piegaia, J. E. Pilcher, A. D. Pilkington, A. W. J. Pin, M. Pinamonti, J. L. Pinfold, H. Pirumov, M. Pitt, L. Plazak, M.-A. Pleier, V. Pleskot, E. Plotnikova, D. Pluth, P. Podberezko, R. Poettgen, L. Poggioli, D. Pohl, G. Polesello, A. Poley, A. Policicchio, R. Polifka, A. Polini, C. S. Pollard, V. Polychronakos, K. Pommès, L. Pontecorvo, B. G. Pope, G. A. Popeneciu, A. Poppleton, S. Pospisil, K. Potamianos, I. N. Potrap, C. J. Potter, C. T. Potter, G. Poulard, J. Poveda, M. E. Pozo Astigarraga, P. Pralavorio, A. Pranko, S. Prell, D. Price, L. E. Price, M. Primavera, S. Prince, K. Prokofiev, F. Prokoshin, S. Protopopescu, J. Proudfoot, M. Przybycien, D. Puddu, A. Puri, P. Puzo, J. Qian, G. Qin, Y. Qin, A. Quadt, W. B. Quayle, M. Queitsch-Maitland, D. Quilty, S. Raddum, V. Radeka, V. Radescu, S. K. Radhakrishnan, P. Radloff, P. Rados, F. Ragusa, G. Rahal, J. A. Raine, S. Rajagopalan, C. Rangel-Smith, M. G. Ratti, D. M. Rauch, F. Rauscher, S. Rave, T. Ravenscroft, I. Ravinovich, M. Raymond, A. L. Read, N. P. Readioff, M. Reale, D. M. Rebuzzi, A. Redelbach, G. Redlinger, R. Reece, R. G. Reed, K. Reeves, L. Rehnisch, J. Reichert, A. Reiss, C. Rembser, H. Ren, M. Rescigno, S. Resconi, E. D. Resseguie, S. Rettie, E. Reynolds, O. L. Rezanova, P. Reznicek, R. Rezvani, R. Richter, S. Richter, E. Richter-Was, O. Ricken, M. Ridel, P. Rieck, C. J. Riegel, J. Rieger, O. Rifki, M. Rijssenbeek, A. Rimoldi, M. Rimoldi, L. Rinaldi, B. Ristić, E. Ritsch, I. Riu, F. Rizatdinova, E. Rizvi, C. Rizzi, R. T. Roberts, S. H. Robertson, A. Robichaud-Veronneau, D. Robinson, J. E. M. Robinson, A. Robson, C. Roda, Y. Rodina, A. Rodriguez Perez, D. Rodriguez Rodriguez, S. Roe, C. S. Rogan, O. Røhne, J. Roloff, A. Romaniouk, M. Romano, S. M. Romano Saez, E. Romero Adam, N. Rompotis, M. Ronzani, L. Roos, S. Rosati, K. Rosbach, P. Rose, N.-A. Rosien, V. Rossetti, E. Rossi, L. P. Rossi, J. H. N. Rosten, R. Rosten, M. Rotaru, I. Roth, J. Rothberg, D. Rousseau, A. Rozanov, Y. Rozen, X. Ruan, F. Rubbo, F. Rühr, A. Ruiz-Martinez, Z. Rurikova, N. A. Rusakovich, A. Ruschke, H. L. Russell, J. P. Rutherfoord, N. Ruthmann, Y. F. Ryabov, M. Rybar, G. Rybkin, S. Ryu, A. Ryzhov, G. F. Rzehorz, A. F. Saavedra, G. Sabato, S. Sacerdoti, H. F-W. Sadrozinski, R. Sadykov, F. Safai Tehrani, P. Saha, M. Sahinsoy, M. Saimpert, M. Saito, T. Saito, H. Sakamoto, Y. Sakurai, G. Salamanna, J. E. Salazar Loyola, D. Salek, P. H. Sales De Bruin, D. Salihagic, A. Salnikov, J. Salt, D. Salvatore, F. Salvatore, A. Salvucci, A. Salzburger, D. Sammel, D. Sampsonidis, J. Sánchez, V. Sanchez Martinez, A. Sanchez Pineda, H. Sandaker, R. L. Sandbach, C. O. Sander, M. Sandhoff, C. Sandoval, D. P. C. Sankey, M. Sannino, A. Sansoni, C. Santoni, R. Santonico, H. Santos, I. Santoyo Castillo, K. Sapp, A. Sapronov, J. G. Saraiva, B. Sarrazin, O. Sasaki, K. Sato, E. Sauvan, G. Savage, P. Savard, N. Savic, C. Sawyer, L. Sawyer, J. Saxon, C. Sbarra, A. Sbrizzi, T. Scanlon, D. A. Scannicchio, M. Scarcella, V. Scarfone, J. Schaarschmidt, P. Schacht, B. M. Schachtner, D. Schaefer, L. Schaefer, R. Schaefer, J. Schaeffer, S. Schaepe, S. Schaetzel, U. Schäfer, A. C. Schaffer, D. Schaile, R. D. Schamberger, V. Scharf, V. A. Schegelsky, D. Scheirich, M. Schernau, C. Schiavi, S. Schier, C. Schillo, M. Schioppa, S. Schlenker, K. R. Schmidt-Sommerfeld, K. Schmieden, C. Schmitt, S. Schmitt, S. Schmitz, B. Schneider, U. Schnoor, L. Schoeffel, A. Schoening, B. D. Schoenrock, E. Schopf, M. Schott, J. F. P. Schouwenberg, J. Schovancova, S. Schramm, N. Schuh, A. Schulte, M. J. Schultens, H.-C. Schultz-Coulon, H. Schulz, M. Schumacher, B. A. Schumm, Ph. Schune, A. Schwartzman, T. A. Schwarz, H. Schweiger, Ph. Schwemling, R. Schwienhorst, J. Schwindling, T. Schwindt, G. Sciolla, F. Scuri, F. Scutti, J. Searcy, P. Seema, S. C. Seidel, A. Seiden, J. M. Seixas, G. Sekhniaidze, K. Sekhon, S. J. Sekula, N. Semprini-Cesari, C. Serfon, L. Serin, L. Serkin, M. Sessa, R. Seuster, H. Severini, T. Sfiligoj, F. Sforza, A. Sfyrla, E. Shabalina, N. W. Shaikh, L. Y. Shan, R. Shang, J. T. Shank, M. Shapiro, P. B. Shatalov, K. Shaw, S. M. Shaw, A. Shcherbakova, C. Y. Shehu, Y. Shen, P. Sherwood, L. Shi, S. Shimizu, C. O. Shimmin, M. Shimojima, S. Shirabe, M. Shiyakova, J. Shlomi, A. Shmeleva, D. Shoaleh Saadi, M. J. Shochet, S. Shojaii, D. R. Shope, S. Shrestha, E. Shulga, M. A. Shupe, P. Sicho, A. M. Sickles, P. E. Sidebo, E. Sideras Haddad, O. Sidiropoulou, D. Sidorov, A. Sidoti, F. Siegert, Dj. Sijacki, J. Silva, S. B. Silverstein, V. Simak, Lj. Simic, S. Simion, E. Simioni, B. Simmons, M. Simon, P. Sinervo, N. B. Sinev, M. Sioli, G. Siragusa, I. Siral, S. Yu. Sivoklokov, J. Sjölin, M. B. Skinner, P. Skubic, M. Slater, T. Slavicek, M. Slawinska, K. Sliwa, R. Slovak, V. Smakhtin, B. H. Smart, L. Smestad, J. Smiesko, S. Yu. Smirnov, Y. Smirnov, L. N. Smirnova, O. Smirnova, J. W. Smith, M. N. K. Smith, R. W. Smith, M. Smizanska, K. Smolek, A. A. Snesarev, I. M. Snyder, S. Snyder, R. Sobie, F. Socher, A. Soffer, D. A. Soh, G. Sokhrannyi, C. A. Solans Sanchez, M. Solar, E. Yu. Soldatov, U. Soldevila, A. A. Solodkov, A. Soloshenko, O. V. Solovyanov, V. Solovyev, P. Sommer, H. Son, H. Y. Song, A. Sopczak, V. Sorin, D. Sosa, C. L. Sotiropoulou, R. Soualah, A. M. Soukharev, D. South, B. C. Sowden, S. Spagnolo, M. Spalla, M. Spangenberg, F. Spanò, D. Sperlich, F. Spettel, T. M. Spieker, R. Spighi, G. Spigo, L. A. Spiller, M. Spousta, R. D. St. Denis, A. Stabile, R. Stamen, S. Stamm, E. Stanecka, R. W. Stanek, C. Stanescu, M. M. Stanitzki, S. Stapnes, E. A. Starchenko, G. H. Stark, J. Stark, S. H Stark, P. Staroba, P. Starovoitov, S. Stärz, R. Staszewski, P. Steinberg, B. Stelzer, H. J. Stelzer, O. Stelzer-Chilton, H. Stenzel, G. A. Stewart, J. A. Stillings, M. C. Stockton, M. Stoebe, G. Stoicea, P. Stolte, S. Stonjek, A. R. Stradling, A. Straessner, M. E. Stramaglia, J. Strandberg, S. Strandberg, A. Strandlie, M. Strauss, P. Strizenec, R. Ströhmer, D. M. Strom, R. Stroynowski, A. Strubig, S. A. Stucci, B. Stugu, N. A. Styles, D. Su, J. Su, S. Suchek, Y. Sugaya, M. Suk, V. V. Sulin, S. Sultansoy, T. Sumida, S. Sun, X. Sun, K. Suruliz, C. J. E. Suster, M. R. Sutton, S. Suzuki, M. Svatos, M. Swiatlowski, S. P. Swift, I. Sykora, T. Sykora, D. Ta, K. Tackmann, J. Taenzer, A. Taffard, R. Tafirout, N. Taiblum, H. Takai, R. Takashima, T. Takeshita, Y. Takubo, M. Talby, A. A. Talyshev, J. Tanaka, M. Tanaka, R. Tanaka, S. Tanaka, R. Tanioka, B. B. Tannenwald, S. Tapia Araya, S. Tapprogge, S. Tarem, G. F. Tartarelli, P. Tas, M. Tasevsky, T. Tashiro, E. Tassi, A. Tavares Delgado, Y. Tayalati, A. C. Taylor, G. N. Taylor, P. T. E. Taylor, W. Taylor, P. Teixeira-Dias, D. Temple, H. Ten Kate, P. K. Teng, J. J. Teoh, F. Tepel, S. Terada, K. Terashi, J. Terron, S. Terzo, M. Testa, R. J. Teuscher, T. Theveneaux-Pelzer, J. P. Thomas, J. Thomas-Wilsker, P. D. Thompson, A. S. Thompson, L. A. Thomsen, E. Thomson, M. J. Tibbetts, R. E. Ticse Torres, V. O. Tikhomirov, Yu. A. Tikhonov, S. Timoshenko, P. Tipton, S. Tisserant, K. Todome, S. Todorova-Nova, J. Tojo, S. Tokár, K. Tokushuku, E. Tolley, L. Tomlinson, M. Tomoto, L. Tompkins, K. Toms, B. Tong, P. Tornambe, E. Torrence, H. Torres, E. Torró Pastor, J. Toth, F. Touchard, D. R. Tovey, C. J. Treado, T. Trefzger, A. Tricoli, I. M. Trigger, S. Trincaz-Duvoid, M. F. Tripiana, W. Trischuk, B. Trocmé, A. Trofymov, C. Troncon, M. Trottier-McDonald, M. Trovatelli, L. Truong, M. Trzebinski, A. Trzupek, K. W. Tsang, J. C-L. Tseng, P. V. Tsiareshka, G. Tsipolitis, N. Tsirintanis, S. Tsiskaridze, V. Tsiskaridze, E. G. Tskhadadze, K. M. Tsui, I. I. Tsukerman, V. Tsulaia, S. Tsuno, D. Tsybychev, Y. Tu, A. Tudorache, V. Tudorache, T. T. Tulbure, A. N. Tuna, S. A. Tupputi, S. Turchikhin, D. Turgeman, I. Turk Cakir, R. Turra, P. M. Tuts, G. Ucchielli, I. Ueda, M. Ughetto, F. Ukegawa, G. Unal, A. Undrus, G. Unel, F. C. Ungaro, Y. Unno, C. Unverdorben, J. Urban, P. Urquijo, P. Urrejola, G. Usai, J. Usui, L. Vacavant, V. Vacek, B. Vachon, C. Valderanis, E. Valdes Santurio, N. Valencic, S. Valentinetti, A. Valero, L. Valéry, S. Valkar, A. Vallier, J. A. Valls Ferrer, W. Van Den Wollenberg, H. van der Graaf, N. van Eldik, P. van Gemmeren, J. Van Nieuwkoop, I. van Vulpen, M. C. van Woerden, M. Vanadia, W. Vandelli, R. Vanguri, A. Vaniachine, P. Vankov, G. Vardanyan, R. Vari, E. W. Varnes, C. Varni, T. Varol, D. Varouchas, A. Vartapetian, K. E. Varvell, J. G. Vasquez, G. A. Vasquez, F. Vazeille, T. Vazquez Schroeder, J. Veatch, V. Veeraraghavan, L. M. Veloce, F. Veloso, S. Veneziano, A. Ventura, M. Venturi, N. Venturi, A. Venturini, V. Vercesi, M. Verducci, W. Verkerke, J. C. Vermeulen, M. C. Vetterli, N. Viaux Maira, O. Viazlo, I. Vichou, T. Vickey, O. E. Vickey Boeriu, G. H. A. Viehhauser, S. Viel, L. Vigani, M. Villa, M. Villaplana Perez, E. Vilucchi, M. G. Vincter, V. B. Vinogradov, A. Vishwakarma, C. Vittori, I. Vivarelli, S. Vlachos, M. Vlasak, M. Vogel, P. Vokac, G. Volpi, M. Volpi, H. von der Schmitt, E. von Toerne, V. Vorobel, K. Vorobev, M. Vos, R. Voss, J. H. Vossebeld, N. Vranjes, M. Vranjes Milosavljevic, V. Vrba, M. Vreeswijk, R. Vuillermet, I. Vukotic, P. Wagner, W. Wagner, H. Wahlberg, S. Wahrmund, J. Wakabayashi, J. Walder, R. Walker, W. Walkowiak, V. Wallangen, C. Wang, C. Wang, F. Wang, H. Wang, H. Wang, J. Wang, J. Wang, Q. Wang, R. Wang, S. M. Wang, T. Wang, W. Wang, W. Wang, C. Wanotayaroj, A. Warburton, C. P. Ward, D. R. Wardrope, A. Washbrook, P. M. Watkins, A. T. Watson, M. F. Watson, G. Watts, S. Watts, B. M. Waugh, A. F. Webb, S. Webb, M. S. Weber, S. W. Weber, S. A. Weber, J. S. Webster, A. R. Weidberg, B. Weinert, J. Weingarten, C. Weiser, H. Weits, P. S. Wells, T. Wenaus, T. Wengler, S. Wenig, N. Wermes, M. D. Werner, P. Werner, M. Wessels, K. Whalen, N. L. Whallon, A. M. Wharton, A. White, M. J. White, R. White, D. Whiteson, F. J. Wickens, W. Wiedenmann, M. Wielers, C. Wiglesworth, L. A. M. Wiik-Fuchs, A. Wildauer, F. Wilk, H. G. Wilkens, H. H. Williams, S. Williams, C. Willis, S. Willocq, J. A. Wilson, I. Wingerter-Seez, F. Winklmeier, O. J. Winston, B. T. Winter, M. Wittgen, M. Wobisch, T. M. H. Wolf, R. Wolff, M. W. Wolter, H. Wolters, S. D. Worm, B. K. Wosiek, J. Wotschack, M. J. Woudstra, K. W. Wozniak, M. Wu, S. L. Wu, X. Wu, Y. Wu, T. R. Wyatt, B. M. Wynne, S. Xella, Z. Xi, L. Xia, D. Xu, L. Xu, B. Yabsley, S. Yacoob, D. Yamaguchi, Y. Yamaguchi, A. Yamamoto, S. Yamamoto, T. Yamanaka, K. Yamauchi, Y. Yamazaki, Z. Yan, H. Yang, H. Yang, Y. Yang, Z. Yang, W-M. Yao, Y. C. Yap, Y. Yasu, E. Yatsenko, K. H. Yau Wong, J. Ye, S. Ye, I. Yeletskikh, E. Yildirim, K. Yorita, K. Yoshihara, C. Young, C. J. S. Young, S. Youssef, D. R. Yu, J. Yu, J. Yu, L. Yuan, S. P. Y. Yuen, I. Yusuff, B. Zabinski, G. Zacharis, R. Zaidan, A. M. Zaitsev, N. Zakharchuk, J. Zalieckas, A. Zaman, S. Zambito, D. Zanzi, C. Zeitnitz, M. Zeman, A. Zemla, J. C. Zeng, Q. Zeng, O. Zenin, T. Ženiš, D. Zerwas, D. Zhang, F. Zhang, G. Zhang, H. Zhang, J. Zhang, L. Zhang, L. Zhang, M. Zhang, R. Zhang, R. Zhang, X. Zhang, Y. Zhang, Z. Zhang, X. Zhao, Y. Zhao, Z. Zhao, A. Zhemchugov, J. Zhong, B. Zhou, C. Zhou, L. Zhou, M. Zhou, M. Zhou, N. Zhou, C. G. Zhu, H. Zhu, J. Zhu, Y. Zhu, X. Zhuang, K. Zhukov, A. Zibell, D. Zieminska, N. I. Zimine, C. Zimmermann, S. Zimmermann, Z. Zinonos, M. Zinser, M. Ziolkowski, L. Živković, G. Zobernig, A. Zoccoli, R. Zou, M. zur Nedden, L. Zwalinski, ATLAS Collaboration

**Affiliations:** 1 Department of Physics, University of Adelaide, Adelaide, Australia; 2 Physics Department, SUNY Albany, Albany, NY USA; 3 Department of Physics, University of Alberta, Edmonton, AB Canada; 4 Department of Physics, Ankara University, Ankara, Turkey; 5 Istanbul Aydin University, Istanbul, Turkey; 6 Division of Physics, TOBB University of Economics and Technology, Ankara, Turkey; 7 LAPP, CNRS/IN2P3 and Université Savoie Mont Blanc, Annecy-le-Vieux, France; 8 High Energy Physics Division, Argonne National Laboratory, Argonne, IL USA; 9 Department of Physics, University of Arizona, Tucson, AZ USA; 10 Department of Physics, The University of Texas at Arlington, Arlington, TX USA; 11 Physics Department, National and Kapodistrian University of Athens, Athens, Greece; 12 Physics Department, National Technical University of Athens, Zografou, Greece; 13 Department of Physics, The University of Texas at Austin, Austin, TX USA; 14 Institute of Physics, Azerbaijan Academy of Sciences, Baku, Azerbaijan; 15 Institut de Física d’Altes Energies (IFAE), The Barcelona Institute of Science and Technology, Barcelona, Spain; 16 Institute of Physics, University of Belgrade, Belgrade, Serbia; 17 Department for Physics and Technology, University of Bergen, Bergen, Norway; 18 Physics Division, Lawrence Berkeley National Laboratory, University of California, Berkeley, CA USA; 19 Department of Physics, Humboldt University, Berlin, Germany; 20 Albert Einstein Center for Fundamental Physics, Laboratory for High Energy Physics, University of Bern, Bern, Switzerland; 21 School of Physics and Astronomy, University of Birmingham, Birmingham, UK; 22 Department of Physics, Bogazici University, Istanbul, Turkey; 23 Department of Physics Engineering, Gaziantep University, Gaziantep, Turkey; 24 Faculty of Engineering and Natural Sciences, Istanbul Bilgi University, Istanbul, Turkey; 25 Faculty of Engineering and Natural Sciences, Bahcesehir University, Istanbul, Turkey; 26 Centro de Investigaciones, Universidad Antonio Narino, Bogotá, Colombia; 27 INFN Sezione di Bologna, Bologna, Italy; 28 Dipartimento di Fisica e Astronomia, Università di Bologna, Bologna, Italy; 29 Physikalisches Institut, University of Bonn, Bonn, Germany; 30 Department of Physics, Boston University, Boston, MA USA; 31 Department of Physics, Brandeis University, Waltham, MA USA; 32 Universidade Federal do Rio De Janeiro COPPE/EE/IF, Rio de Janeiro, Brazil; 33 Electrical Circuits Department, Federal University of Juiz de Fora (UFJF), Juiz de Fora, Brazil; 34 Federal University of Sao Joao del Rei (UFSJ), Sao Joao del Rei, Brazil; 35 Instituto de Fisica, Universidade de Sao Paulo, São Paulo, Brazil; 36 Physics Department, Brookhaven National Laboratory, Upton, NY USA; 37 Transilvania University of Brasov, Brasov, Romania; 38 Horia Hulubei National Institute of Physics and Nuclear Engineering, Bucharest, Romania; 39 Department of Physics, Alexandru Ioan Cuza University of Iasi, Iasi, Romania; 40 Physics Department, National Institute for Research and Development of Isotopic and Molecular Technologies, Cluj-Napoca, Romania; 41 University Politehnica Bucharest, Bucharest, Romania; 42 West University in Timisoara, Timisoara, Romania; 43 Departamento de Física, Universidad de Buenos Aires, Buenos Aires, Argentina; 44 Cavendish Laboratory, University of Cambridge, Cambridge, UK; 45 Department of Physics, Carleton University, Ottawa, ON Canada; 46 CERN, Geneva, Switzerland; 47 Enrico Fermi Institute, University of Chicago, Chicago, IL USA; 48 Departamento de Física, Pontificia Universidad Católica de Chile, Santiago, Chile; 49 Departamento de Física, Universidad Técnica Federico Santa María, Valparaiso, Chile; 50 Institute of High Energy Physics, Chinese Academy of Sciences, Beijing, China; 51 Department of Physics, Nanjing University, Nanjing, Jiangsu China; 52 Physics Department, Tsinghua University, Beijing, 100084 China; 53 Department of Modern Physics, University of Science and Technology of China, Hefei, Anhui China; 54 School of Physics, Shandong University, Jinan, Shandong China; 55 Department of Physics and Astronomy, Key Laboratory for Particle Physics, Astrophysics and Cosmology, Ministry of Education, Shanghai Key Laboratory for Particle Physics and Cosmology, Shanghai Jiao Tong University, Shanghai (also at PKU-CHEP), Shanghai, China; 56 Université Clermont Auvergne, CNRS/IN2P3, LPC, Clermont-Ferrand, France; 57 Nevis Laboratory, Columbia University, Irvington, NY USA; 58 Niels Bohr Institute, University of Copenhagen, Copenhagen, Denmark; 59 INFN Gruppo Collegato di Cosenza, Laboratori Nazionali di Frascati, Frascati, Italy; 60 Dipartimento di Fisica, Università della Calabria, Rende, Italy; 61 Faculty of Physics and Applied Computer Science, AGH University of Science and Technology, Kraków, Poland; 62 Marian Smoluchowski Institute of Physics, Jagiellonian University, Kraków, Poland; 63 Institute of Nuclear Physics, Polish Academy of Sciences, Kraków, Poland; 64 Physics Department, Southern Methodist University, Dallas, TX USA; 65 Physics Department, University of Texas at Dallas, c, TX USA; 66 DESY, Hamburg and Zeuthen, Germany; 67 Lehrstuhl für Experimentelle Physik IV, Technische Universität Dortmund, Dortmund, Germany; 68 Institut für Kern- und Teilchenphysik, Technische Universität Dresden, Dresden, Germany; 69 Department of Physics, Duke University, Durham, NC USA; 70 SUPA-School of Physics and Astronomy, University of Edinburgh, Edinburgh, UK; 71 INFN Laboratori Nazionali di Frascati, Frascati, Italy; 72 Fakultät für Mathematik und Physik, Albert-Ludwigs-Universität, Freiburg, Germany; 73 Departement de Physique Nucleaire et Corpusculaire, Université de Genève, Geneva, Switzerland; 74 INFN Sezione di Genova, Genoa, Italy; 75 Dipartimento di Fisica, Università di Genova, Genoa, Italy; 76 E. Andronikashvili Institute of Physics, Iv. Javakhishvili Tbilisi State University, Tbilisi, Georgia; 77 High Energy Physics Institute, Tbilisi State University, Tbilisi, Georgia; 78 II Physikalisches Institut, Justus-Liebig-Universität Giessen, Giessen, Germany; 79 SUPA-School of Physics and Astronomy, University of Glasgow, Glasgow, UK; 80 II Physikalisches Institut, Georg-August-Universität, Göttingen, Germany; 81 Laboratoire de Physique Subatomique et de Cosmologie, Université Grenoble-Alpes, CNRS/IN2P3, Grenoble, France; 82 Laboratory for Particle Physics and Cosmology, Harvard University, Cambridge, MA USA; 83 Kirchhoff-Institut für Physik, Ruprecht-Karls-Universität Heidelberg, Heidelberg, Germany; 84 Physikalisches Institut, Ruprecht-Karls-Universität Heidelberg, Heidelberg, Germany; 85 ZITI Institut für technische Informatik, Ruprecht-Karls-Universität Heidelberg, Mannheim, Germany; 86 Faculty of Applied Information Science, Hiroshima Institute of Technology, Hiroshima, Japan; 87 Department of Physics, The Chinese University of Hong Kong, Shatin, NT Hong Kong; 88 Department of Physics, The University of Hong Kong, Hong Kong, China; 89 Department of Physics, Institute for Advanced Study, The Hong Kong University of Science and Technology, Clear Water Bay, Kowloon, Hong Kong, China; 90 Department of Physics, National Tsing Hua University, Taiwan, Taiwan; 91 Department of Physics, Indiana University, Bloomington, IN USA; 92 Institut für Astro- und Teilchenphysik, Leopold-Franzens-Universität, Innsbruck, Austria; 93 University of Iowa, Iowa City, IA USA; 94 Department of Physics and Astronomy, Iowa State University, Ames, IA USA; 95 Joint Institute for Nuclear Research, JINR Dubna, Dubna, Russia; 96 KEK, High Energy Accelerator Research Organization, Tsukuba, Japan; 97 Graduate School of Science, Kobe University, Kobe, Japan; 98 Faculty of Science, Kyoto University, Kyoto, Japan; 99 Kyoto University of Education, Kyoto, Japan; 100 Department of Physics, Kyushu University, Fukuoka, Japan; 101 Instituto de Física La Plata, Universidad Nacional de La Plata and CONICET, La Plata, Argentina; 102 Physics Department, Lancaster University, Lancaster, UK; 103 INFN Sezione di Lecce, Lecce, Italy; 104 Dipartimento di Matematica e Fisica, Università del Salento, Lecce, Italy; 105 Oliver Lodge Laboratory, University of Liverpool, Liverpool, UK; 106 Department of Experimental Particle Physics, Jožef Stefan Institute and Department of Physics, University of Ljubljana, Ljubljana, Slovenia; 107 School of Physics and Astronomy, Queen Mary University of London, London, UK; 108 Department of Physics, Royal Holloway University of London, Surrey, UK; 109 Department of Physics and Astronomy, University College London, London, UK; 110 Louisiana Tech University, Ruston, LA USA; 111 Laboratoire de Physique Nucléaire et de Hautes Energies, UPMC and Université Paris-Diderot and CNRS/IN2P3, Paris, France; 112 Fysiska institutionen, Lunds universitet, Lund, Sweden; 113 Departamento de Fisica Teorica C-15, Universidad Autonoma de Madrid, Madrid, Spain; 114 Institut für Physik, Universität Mainz, Mainz, Germany; 115 School of Physics and Astronomy, University of Manchester, Manchester, UK; 116 CPPM, Aix-Marseille Université and CNRS/IN2P3, Marseille, France; 117 Department of Physics, University of Massachusetts, Amherst, MA USA; 118 Department of Physics, McGill University, Montreal, QC Canada; 119 School of Physics, University of Melbourne, Victoria, Australia; 120 Department of Physics, The University of Michigan, Ann Arbor, MI USA; 121 Department of Physics and Astronomy, Michigan State University, East Lansing, MI USA; 122 INFN Sezione di Milano, Milan, Italy; 123 Dipartimento di Fisica, Università di Milano, Milan, Italy; 124 B.I. Stepanov Institute of Physics, National Academy of Sciences of Belarus, Minsk, Republic of Belarus; 125 Research Institute for Nuclear Problems of Byelorussian State University, Minsk, Republic of Belarus; 126 Group of Particle Physics, University of Montreal, Montreal, QC Canada; 127 P.N. Lebedev Physical Institute of the Russian Academy of Sciences, Moscow, Russia; 128 Institute for Theoretical and Experimental Physics (ITEP), Moscow, Russia; 129 National Research Nuclear University MEPhI, Moscow, Russia; 130 D.V. Skobeltsyn Institute of Nuclear Physics, M.V. Lomonosov Moscow State University, Moscow, Russia; 131 Fakultät für Physik, Ludwig-Maximilians-Universität München, Munich, Germany; 132 Max-Planck-Institut für Physik (Werner-Heisenberg-Institut), Munich, Germany; 133 Nagasaki Institute of Applied Science, Nagasaki, Japan; 134 Graduate School of Science and Kobayashi-Maskawa Institute, Nagoya University, Nagoya, Japan; 135 INFN Sezione di Napoli, Naples, Italy; 136 Dipartimento di Fisica, Università di Napoli, Naples, Italy; 137 Department of Physics and Astronomy, University of New Mexico, Albuquerque, NM USA; 138 Institute for Mathematics, Astrophysics and Particle Physics, Radboud University Nijmegen/Nikhef, Nijmegen, The Netherlands; 139 Nikhef National Institute for Subatomic Physics, University of Amsterdam, Amsterdam, The Netherlands; 140 Department of Physics, Northern Illinois University, DeKalb, IL USA; 141 Budker Institute of Nuclear Physics, SB RAS, Novosibirsk, Russia; 142 Department of Physics, New York University, New York, NY USA; 143 Ohio State University, Columbus, OH USA; 144 Faculty of Science, Okayama University, Okayama, Japan; 145 Homer L. Dodge Department of Physics and Astronomy, University of Oklahoma, Norman, OK USA; 146 Department of Physics, Oklahoma State University, Stillwater, OK USA; 147 Palacký University, RCPTM, Olomouc, Czech Republic; 148 Center for High Energy Physics, University of Oregon, Eugene, OR USA; 149 LAL, Univ. Paris-Sud, CNRS/IN2P3, Université Paris-Saclay, Orsay, France; 150 Graduate School of Science, Osaka University, Osaka, Japan; 151 Department of Physics, University of Oslo, Oslo, Norway; 152 Department of Physics, Oxford University, Oxford, UK; 153 INFN Sezione di Pavia, Pavia, Italy; 154 Dipartimento di Fisica, Università di Pavia, Pavia, Italy; 155 Department of Physics, University of Pennsylvania, Philadelphia, PA USA; 156 National Research Centre “Kurchatov Institute” B.P. Konstantinov Petersburg Nuclear Physics Institute, St. Petersburg, Russia; 157 INFN Sezione di Pisa, Pisa, Italy; 158 Dipartimento di Fisica E. Fermi, Università di Pisa, Pisa, Italy; 159 Department of Physics and Astronomy, University of Pittsburgh, Pittsburgh, PA USA; 160 Laboratório de Instrumentação e Física Experimental de Partículas-LIP, Lisbon, Portugal; 161 Faculdade de Ciências, Universidade de Lisboa, Lisbon, Portugal; 162 Department of Physics, University of Coimbra, Coimbra, Portugal; 163 Centro de Física Nuclear da Universidade de Lisboa, Lisbon, Portugal; 164 Departamento de Fisica, Universidade do Minho, Braga, Portugal; 165 Departamento de Fisica Teorica y del Cosmos and CAFPE, Universidad de Granada, Granada, Spain; 166 Dep Fisica and CEFITEC of Faculdade de Ciencias e Tecnologia, Universidade Nova de Lisboa, Caparica, Portugal; 167 Institute of Physics, Academy of Sciences of the Czech Republic, Prague, Czech Republic; 168 Czech Technical University in Prague, Prague, Czech Republic; 169 Faculty of Mathematics and Physics, Charles University, Prague, Czech Republic; 170 State Research Center Institute for High Energy Physics (Protvino), NRC KI, Protvino, Russia; 171 Particle Physics Department, Rutherford Appleton Laboratory, Didcot, UK; 172 INFN Sezione di Roma, Rome, Italy; 173 Dipartimento di Fisica, Sapienza Università di Roma, Rome, Italy; 174 INFN Sezione di Roma Tor Vergata, Rome, Italy; 175 Dipartimento di Fisica, Università di Roma Tor Vergata, Rome, Italy; 176 INFN Sezione di Roma Tre, Rome, Italy; 177 Dipartimento di Matematica e Fisica, Università Roma Tre, Rome, Italy; 178 Faculté des Sciences Ain Chock, Réseau Universitaire de Physique des Hautes Energies-Université Hassan II, Casablanca, Morocco; 179 Centre National de l’Energie des Sciences Techniques Nucleaires, Rabat, Morocco; 180 Faculté des Sciences Semlalia, Université Cadi Ayyad, LPHEA-Marrakech, Marrakech, Morocco; 181 Faculté des Sciences, Université Mohamed Premier and LPTPM, Oujda, Morocco; 182 Faculté des Sciences, Université Mohammed V, Rabat, Morocco; 183 DSM/IRFU (Institut de Recherches sur les Lois Fondamentales de l’Univers), CEA Saclay (Commissariat à l’Energie Atomique et aux Energies Alternatives), Gif-sur-Yvette, France; 184 Santa Cruz Institute for Particle Physics, University of California Santa Cruz, Santa Cruz, CA USA; 185 Department of Physics, University of Washington, Seattle, WA USA; 186 Department of Physics and Astronomy, University of Sheffield, Sheffield, UK; 187 Department of Physics, Shinshu University, Nagano, Japan; 188 Department Physik, Universität Siegen, Siegen, Germany; 189 Department of Physics, Simon Fraser University, Burnaby, BC Canada; 190 SLAC National Accelerator Laboratory, Stanford, CA USA; 191 Faculty of Mathematics, Physics and Informatics, Comenius University, Bratislava, Slovak Republic; 192 Department of Subnuclear Physics, Institute of Experimental Physics of the Slovak Academy of Sciences, Kosice, Slovak Republic; 193 Department of Physics, University of Cape Town, Cape Town, South Africa; 194 Department of Physics, University of Johannesburg, Johannesburg, South Africa; 195 School of Physics, University of the Witwatersrand, Johannesburg, South Africa; 196 Department of Physics, Stockholm University, Stockholm, Sweden; 197 The Oskar Klein Centre, Stockholm, Sweden; 198 Physics Department, Royal Institute of Technology, Stockholm, Sweden; 199 Departments of Physics and Astronomy and Chemistry, Stony Brook University, Stony Brook, NY USA; 200 Department of Physics and Astronomy, University of Sussex, Brighton, UK; 201 School of Physics, University of Sydney, Sydney, Australia; 202 Institute of Physics, Academia Sinica, Taipei, Taiwan; 203 Department of Physics, Technion: Israel Institute of Technology, Haifa, Israel; 204 Raymond and Beverly Sackler School of Physics and Astronomy, Tel Aviv University, Tel Aviv, Israel; 205 Department of Physics, Aristotle University of Thessaloniki, Thessaloníki, Greece; 206 International Center for Elementary Particle Physics and Department of Physics, The University of Tokyo, Tokyo, Japan; 207 Graduate School of Science and Technology, Tokyo Metropolitan University, Tokyo, Japan; 208 Department of Physics, Tokyo Institute of Technology, Tokyo, Japan; 209 Tomsk State University, Tomsk, Russia; 210 Department of Physics, University of Toronto, Toronto, ON Canada; 211 INFN-TIFPA, Trento, Italy; 212 University of Trento, Trento, Italy; 213 TRIUMF, Vancouver, BC Canada; 214 Department of Physics and Astronomy, York University, Toronto, ON Canada; 215 Faculty of Pure and Applied Sciences, and Center for Integrated Research in Fundamental Science and Engineering, University of Tsukuba, Tsukuba, Japan; 216 Department of Physics and Astronomy, Tufts University, Medford, MA USA; 217 Department of Physics and Astronomy, University of California Irvine, Irvine, CA USA; 218 INFN Gruppo Collegato di Udine, Sezione di Trieste, Udine, Italy; 219 ICTP, Trieste, Italy; 220 Dipartimento di Chimica, Fisica e Ambiente, Università di Udine, Udine, Italy; 221 Department of Physics and Astronomy, University of Uppsala, Uppsala, Sweden; 222 Department of Physics, University of Illinois, Urbana, IL USA; 223 Instituto de Fisica Corpuscular (IFIC) and Departamento de Fisica Atomica, Molecular y Nuclear and Departamento de Ingeniería Electrónica and Instituto de Microelectrónica de Barcelona (IMB-CNM), University of Valencia and CSIC, Valencia, Spain; 224 Department of Physics, University of British Columbia, Vancouver, BC Canada; 225 Department of Physics and Astronomy, University of Victoria, Victoria, BC Canada; 226 Department of Physics, University of Warwick, Coventry, UK; 227 Waseda University, Tokyo, Japan; 228 Department of Particle Physics, The Weizmann Institute of Science, Rehovot, Israel; 229 Department of Physics, University of Wisconsin, Madison, WI USA; 230 Fakultät für Physik und Astronomie, Julius-Maximilians-Universität, Würzburg, Germany; 231 Fakultät für Mathematik und Naturwissenschaften, Fachgruppe Physik, Bergische Universität Wuppertal, Wuppertal, Germany; 232 Department of Physics, Yale University, New Haven, CT USA; 233 Yerevan Physics Institute, Yerevan, Armenia; 234 Centre de Calcul de l’Institut National de Physique Nucléaire et de Physique des Particules (IN2P3), Villeurbanne, France; 235 CERN, 1211 Geneva 23, Switzerland

## Abstract

A measurement of the mass of the *W* boson is presented based on proton–proton collision data recorded in 2011 at a centre-of-mass energy of 7 TeV with the ATLAS detector at the LHC, and corresponding to $4.6~\hbox {fb}^{-1}$ of integrated luminosity. The selected data sample consists of $7.8\times 10^6$ candidates in the $W\rightarrow \mu \nu$ channel and $5.9\times 10^6$ candidates in the $W\rightarrow e \nu$ channel. The *W*-boson mass is obtained from template fits to the reconstructed distributions of the charged lepton transverse momentum and of the *W* boson transverse mass in the electron and muon decay channels, yielding

$$\begin{aligned} \nonumber m_W = 80370&\pm 7~(\text {stat.}) \pm 11 (\text {exp. syst.}) \\&\pm 14~(\text {mod. syst.})~\text {MeV} \\ = 80370&\pm 19\,\text {MeV}, \end{aligned}$$

where the first uncertainty is statistical, the second corresponds to the experimental systematic uncertainty, and the third to the physics-modelling systematic uncertainty. A measurement of the mass difference between the $W^+$ and $W^-$ bosons yields $m_{W^+}-m_{W^-} = -\,29 \pm 28$ MeV.

## Introduction

The Standard Model (SM) of particle physics describes the electroweak interactions as being mediated by the *W* boson, the *Z* boson, and the photon, in a gauge theory based on the ${\mathrm {SU}}(2)_{\mathrm {L}} \times {\mathrm {U}}(1)_{\mathrm {Y}}$ symmetry [1–3]. The theory incorporates the observed masses of the *W* and *Z* bosons through a symmetry-breaking mechanism. In the SM, this mechanism relies on the interaction of the gauge bosons with a scalar doublet field and implies the existence of an additional physical state known as the Higgs boson [4–7]. The existence of the *W* and *Z* bosons was first established at the CERN SPS in 1983 [8–11], and the LHC collaborations ATLAS and CMS reported the discovery of the Higgs boson in 2012 [12, 13].

At lowest order in the electroweak theory, the *W*-boson mass, $m_W$, can be expressed solely as a function of the *Z*-boson mass, $m_Z$, the fine-structure constant, $\alpha$, and the Fermi constant, $G_{\mu }$. Higher-order corrections introduce an additional dependence of the *W*-boson mass on the gauge couplings and the masses of the heavy particles of the SM. The mass of the *W* boson can be expressed in terms of the other SM parameters as follows:

$$\begin{aligned} \nonumber m_W^2 \left( 1 - \frac{m^2_W}{m^2_Z}\right) = \frac{\pi \alpha }{\sqrt{2}G_{\mu }} (1+\Delta r), \end{aligned}$$

where $\Delta r$ incorporates the effect of higher-order corrections [14, 15]. In the SM, $\Delta r$ is in particular sensitive to the top-quark and Higgs-boson masses; in extended theories, $\Delta r$ receives contributions from additional particles and interactions. These effects can be probed by comparing the measured and predicted values of $m_W$. In the context of global fits to the SM parameters, constraints on physics beyond the SM are currently limited by the *W*-boson mass measurement precision [16]. Improving the precision of the measurement of $m_W$ is therefore of high importance for testing the overall consistency of the SM.

Previous measurements of the mass of the *W* boson were performed at the CERN SPS proton–antiproton ($p\bar{p}$ ) collider with the UA1 and UA2 experiments [17, 18] at centre-of-mass energies of $\sqrt{s}=546\,\text {GeV}$ and $\sqrt{s}=630\,\text {GeV}$, at the Tevatron $p\bar{p}$ collider with the CDF and D0 detectors at $\sqrt{s}=1.8\,\text {TeV}$ [19–21] and $\sqrt{s}=1.96\,\text {TeV}$ [22–24], and at the LEP electron–positron collider by the ALEPH, DELPHI, L3, and OPAL collaborations at $\sqrt{s}=161$–$209\,\text {GeV}$ [25–28]. The current Particle Data Group world average value of $m_W = 80385 \pm 15$ $\,\text {MeV}$ [29] is dominated by the CDF and D0 measurements performed at $\sqrt{s}=1.96\,\text {TeV}$. Given the precisely measured values of $\alpha$, $G_{\mu }$ and $m_Z$, and taking recent top-quark and Higgs-boson mass measurements, the SM prediction of $m_W$ is $m_W=80358\pm 8$ MeV in Ref. [16] and $m_W=80362\pm 8$ $\,\text {MeV}$ in Ref. [30]. The SM prediction uncertainty of 8 $\,\text {MeV}$ represents a target for the precision of future measurements of $m_W$.

At hadron colliders, the *W*-boson mass can be determined in Drell–Yan production [31] from $W\rightarrow \ell \nu$ decays, where $\ell$ is an electron or muon. The mass of the *W* boson is extracted from the Jacobian edges of the final-state kinematic distributions, measured in the plane perpendicular to the beam direction. Sensitive observables include the transverse momenta of the charged lepton and neutrino and the *W*-boson transverse mass.

The ATLAS and CMS experiments benefit from large signal and calibration samples. The numbers of selected *W*- and *Z*-boson events, collected in a sample corresponding to approximately 4.6 fb$^{-1}$ of integrated luminosity at a centre-of-mass energy of $7\,\text {TeV}$, are of the order of $10^7$ for the $W\rightarrow \ell \nu$, and of the order of $10^6$ for the $Z\rightarrow \ell \ell$ processes. The available data sample is therefore larger by an order of magnitude compared to the corresponding samples used for the CDF and D0 measurements. Given the precisely measured value of the *Z*-boson mass [32] and the clean leptonic final state, the $Z\rightarrow \ell \ell$ processes provide the primary constraints for detector calibration, physics modelling, and validation of the analysis strategy. The sizes of these samples correspond to a statistical uncertainty smaller than 10 $\,\text {MeV}$ in the measurement of the *W*-boson mass.

Measurements of $m_W$ at the LHC are affected by significant complications related to the strong interaction. In particular, in proton–proton (*pp*) collisions at $\sqrt{s}=7$ $\text {TeV}$, approximately 25% of the inclusive *W*-boson production rate is induced by at least one second-generation quark, *s* or *c*, in the initial state. The amount of heavy-quark-initiated production has implications for the *W*-boson rapidity and transverse-momentum distributions [33]. As a consequence, the measurement of the *W*-boson mass is sensitive to the strange-quark and charm-quark parton distribution functions (PDFs) of the proton. In contrast, second-generation quarks contribute only to approximately 5% of the overall *W*-boson production rate at the Tevatron. Other important aspects of the measurement of the *W*-boson mass are the theoretical description of electroweak corrections, in particular the modelling of photon radiation from the *W*- and *Z*-boson decay leptons, and the modelling of the relative fractions of helicity cross sections in the Drell–Yan processes [34].

This paper is structured as follows. Section 2 presents an overview of the measurement strategy. Section 3 describes the ATLAS detector. Section 4 describes the data and simulation samples used for the measurement. Section 5 describes the object reconstruction and the event selection. Section 6 summarises the modelling of vector-boson production and decay, with emphasis on the QCD effects outlined above. Sections 7 and 8 are dedicated to the electron, muon, and recoil calibration procedures. Section 9 presents a set of validation tests of the measurement procedure, performed using the *Z*-boson event sample. Section 10 describes the analysis of the *W*-boson sample. Section 11 presents the extraction of $m_W$. The results are summarised in Sect. 12.

## Measurement overview 

This section provides the definition of the observables used in the analysis, an overview of the measurement strategy for the determination of the mass of the *W* boson, and a description of the methodology used to estimate the systematic uncertainties.

### Observable definitions 

ATLAS uses a right-handed coordinate system with its origin at the nominal interaction point (IP) in the centre of the detector and the *z*-axis along the beam pipe. The *x*-axis points from the IP to the centre of the LHC ring, and the *y*-axis points upward. Cylindrical coordinates $(r,\phi )$ are used in the transverse plane, $\phi$ being the azimuth around the *z*-axis. The pseudorapidity is defined in terms of the polar angle $\theta$ as $\eta =-\ln \tan (\theta /2)$.

The kinematic properties of charged leptons from *W*- and *Z*-boson decays are characterised by the measured transverse momentum, $p_{\text {T}} ^{\ell }$, pseudorapidity, $\eta _{\ell }$, and azimuth, $\phi _{\ell }$. The mass of the lepton, $m_{\ell }$, completes the four-vector. For *Z*-boson events, the invariant mass, $m_{\ell \ell }$, the rapidity, $y_{\ell \ell }$, and the transverse momentum, $p_{\text {T}} ^{\ell \ell }$, are obtained by combining the four-momenta of the decay-lepton pair.

The recoil in the transverse plane, $\vec {u}_{\mathrm {T}}$, is reconstructed from the vector sum of the transverse energy of all clusters reconstructed in the calorimeters (Sect. 3), excluding energy deposits associated with the decay leptons. It is defined as:

$$\begin{aligned} \vec {u}_{\mathrm {T}}= \sum _i \vec {E}_{\mathrm {T},i}, \end{aligned}$$

where $\vec {E}_{\mathrm {T},i}$ is the vector of the transverse energy of cluster *i*. The transverse-energy vector of a cluster has magnitude $E_{\mathrm {T}} = E / \cosh \eta$, with the energy deposit of the cluster *E* and its pseudorapidity $\eta$. The azimuth $\phi$ of the transverse-energy vector is defined from the coordinates of the cluster in the transverse plane. In *W*- and *Z*-boson events, $-\vec {u}_{\mathrm {T}}$ provides an estimate of the boson transverse momentum. The related quantities $u_x$ and $u_y$ are the projections of the recoil onto the axes of the transverse plane in the ATLAS coordinate system. In *Z*-boson events, $u_{\parallel }^Z$ and $u_{\perp }^Z$ represent the projections of the recoil onto the axes parallel and perpendicular to the *Z*-boson transverse momentum reconstructed from the decay-lepton pair. Whereas $u_{\parallel }^Z$ can be compared to $-p_{\mathrm {T}}^{\ell \ell }$ and probes the detector response to the recoil in terms of linearity and resolution, the $u_{\perp }^Z$ distribution satisfies $\left\langle u_{\perp }^Z \right\rangle =0$ and its width provides an estimate of the recoil resolution. In *W*-boson events, $u_{\parallel }^\ell$ and $u_{\perp }^\ell$ are the projections of the recoil onto the axes parallel and perpendicular to the reconstructed charged-lepton transverse momentum.

The resolution of the recoil is affected by additional event properties, namely the per-event number of *pp* interactions per bunch crossing (pile-up) ${\mu }$, the average number of *pp* interactions per bunch crossing $\left\langle \mu \right\rangle$, the total reconstructed transverse energy, defined as the scalar sum of the transverse energy of all calorimeter clusters, $\Sigma E_{\mathrm {T}} \equiv \sum _{i} E_{{\mathrm {T}},i}$, and the quantity $\Sigma E^{*}_{\mathrm {T}} \equiv \Sigma E_{\mathrm {T}} - |\vec u_{\mathrm {T}}|$. The latter is less correlated with the recoil than $\Sigma E_{\mathrm {T}}$, and better represents the event activity related to the pile-up and to the underlying event.

The magnitude and direction of the transverse-momentum vector of the decay neutrino, $\vec {p}_\text {T}^{\,\nu }$, are inferred from the vector of the missing transverse momentum, $\vec {p}_{\text {T}}^{\,\text {miss}}$, which corresponds to the momentum imbalance in the transverse plane and is defined as:

$$\begin{aligned} \vec {p}_{\text {T}}^{\,\text {miss}} = -\left( \vec {p}_{\mathrm {T}}^{\,\ell } + \vec {u}_{\mathrm {T}}\right) . \end{aligned}$$

The *W*-boson transverse mass, $m_{\mathrm {T}}$, is derived from $p_{\text {T}}^{\text {miss}}$ and from the transverse momentum of the charged lepton as follows:

$$\begin{aligned} m_{\mathrm {T}}= \sqrt{2 p_{\text {T}} ^\ell p_{\text {T}}^{\text {miss}} (1-\cos {\Delta \phi })}, \end{aligned}$$

where $\Delta \phi$ is the azimuthal opening angle between the charged lepton and the missing transverse momentum.

All vector-boson masses and widths are defined in the running-width scheme. Resonances are expressed by the relativistic Breit–Wigner mass distribution:

1 $$\begin{aligned} \frac{\text {d}\sigma }{\text {d}m} \propto \frac{m^2}{(m^2-m_V^2)^2+m^4\Gamma _V^2/m_V^2}, \end{aligned}$$

where *m* is the invariant mass of the vector-boson decay products, and $m_V$ and $\Gamma _V$, with $V = W,Z$, are the vector-boson masses and widths, respectively. This scheme was introduced in Ref. [35], and is consistent with earlier measurements of the *W*- and *Z*-boson resonance parameters [24, 32].

### Analysis strategy

The mass of the *W* boson is determined from fits to the transverse momentum of the charged lepton, $p_{\text {T}} ^\ell$, and to the transverse mass of the *W* boson, $m_{\mathrm {T}}$. For *W* bosons at rest, the transverse-momentum distributions of the *W* decay leptons have a Jacobian edge at a value of *m* / 2, whereas the distribution of the transverse mass has an endpoint at the value of *m* [36], where *m* is the invariant mass of the charged-lepton and neutrino system, which is related to $m_W$ through the Breit–Wigner distribution of Eq. (1).

The expected final-state distributions, referred to as templates, are simulated for several values of $m_W$ and include signal and background contributions. The templates are compared to the observed distribution by means of a $\chi ^2$ compatibility test. The $\chi ^2$ as a function of $m_W$ is interpolated, and the measured value is determined by analytical minimisation of the $\chi ^2$ function. Predictions for different values of $m_W$ are obtained from a single simulated reference sample, by reweighting the *W*-boson invariant mass distribution according to the Breit–Wigner parameterisation of Eq. (1). The *W*-boson width is scaled accordingly, following the SM relation $\Gamma _W \propto m_W^3$.

Experimentally, the $p_{\text {T}} ^\ell$ and $p_{\text {T}}^{\text {miss}}$ distributions are affected by the lepton energy calibration. The latter is also affected by the calibration of the recoil. The $p_{\text {T}} ^\ell$ and $p_{\text {T}}^{\text {miss}}$ distributions are broadened by the *W*-boson transverse-momentum distribution, and are sensitive to the *W*-boson helicity states, which are influenced by the proton PDFs [37]. Compared to $p_{\text {T}} ^\ell$, the $m_{\mathrm {T}}$ distribution has larger uncertainties due to the recoil, but smaller sensitivity to such physics-modelling effects. Imperfect modelling of these effects can distort the template distributions, and constitutes a significant source of uncertainties for the determination of $m_W$.

The calibration procedures described in this paper rely mainly on methods and results published earlier by ATLAS [38–40], and based on *W* and *Z* samples at $\sqrt{s}=7$ $\text {TeV}$ and $\sqrt{s}=8\,\text {TeV}$. The $Z\rightarrow \ell \ell$ event samples are used to calibrate the detector response. Lepton momentum corrections are derived exploiting the precisely measured value of the *Z*-boson mass, $m_Z$ [32], and the recoil response is calibrated using the expected momentum balance with $p_{\mathrm {T}}^{\ell \ell }$. Identification and reconstruction efficiency corrections are determined from *W*- and *Z*-boson events using the tag-and-probe method [38, 40]. The dependence of these corrections on $p_{\text {T}} ^\ell$ is important for the measurement of $m_W$, as it affects the shape of the template distributions.

The detector response corrections and the physics modelling are verified in *Z*-boson events by performing measurements of the *Z*-boson mass with the same method used to determine the *W*-boson mass, and comparing the results to the LEP combined value of $m_Z$, which is used as input for the lepton calibration. The determination of $m_Z$ from the lepton-pair invariant mass provides a first closure test of the lepton energy calibration. In addition, the extraction of $m_Z$ from the $p_{\text {T}} ^\ell$ distribution tests the $p_{\text {T}} ^\ell$-dependence of the efficiency corrections, and the modelling of the *Z*-boson transverse-momentum distribution and of the relative fractions of *Z*-boson helicity states. The $p_{\text {T}}^{\text {miss}}$ and $m_{\mathrm {T}}$ variables are defined in *Z*-boson events by treating one of the reconstructed decay leptons as a neutrino. The extraction of $m_Z$ from the $m_{\mathrm {T}}$ distribution provides a test of the recoil calibration. The combination of the extraction of $m_Z$ from the $m_{\ell \ell }$, $p_{\text {T}} ^\ell$ and $m_{\mathrm {T}}$ distributions provides a closure test of the measurement procedure. The precision of this validation procedure is limited by the finite size of the *Z*-boson sample, which is approximately ten times smaller than the *W*-boson sample.

**Table 1:** Summary of categories and kinematic distributions used in the $m_W$ measurement analysis for the electron and muon decay channels

Decay channel	$W\rightarrow e\nu$	$W\rightarrow \mu \nu$
Kinematic distributions	$p_{\text {T}} ^\ell$, $m_{\mathrm {T}}$	$p_{\text {T}} ^\ell$, $m_{\mathrm {T}}$
Charge categories	$W^+$, $W^-$	$W^+$, $W^-$
$|\eta _\ell |$ categories	[0, 0.6], [0.6, 1.2], [1.8, 2.4]	[0, 0.8], [0.8, 1.4], [1.4, 2.0], [2.0, 2.4]

The analysis of the *Z*-boson sample does not probe differences in the modelling of *W*- and *Z*-boson production processes. Whereas *W*-boson production at the Tevatron is charge symmetric and dominated by interactions with at least one valence quark, the sea-quark PDFs play a larger role at the LHC, and contributions from processes with heavy quarks in the initial state have to be modelled properly. The $W^+$-boson production rate exceeds that of $W^-$ bosons by about 40%, with a broader rapidity distribution and a softer transverse-momentum distribution. Uncertainties in the modelling of these distributions and in the relative fractions of the *W*-boson helicity states are constrained using measurements of *W*- and *Z*-boson production performed with the ATLAS experiment at $\sqrt{s}=7$ $\text {TeV}$ and $\sqrt{s}=8$ $\text {TeV}$ [41–45].

The final measured value of the *W*-boson mass is obtained from the combination of various measurements performed in the electron and muon decay channels, and in charge- and $|\eta _\ell |$-dependent categories, as defined in Table 1. The boundaries of the $|\eta _\ell |$ categories are driven mainly by experimental and statistical constraints. The measurements of $m_W$ used in the combination are based on the observed distributions of $p_{\text {T}} ^\ell$ and $m_{\mathrm {T}}$, which are only partially correlated. Measurements of $m_W$ based on the $p_{\text {T}}^{\text {miss}}$ distributions are performed as consistency tests, but they are not used in the combination due to their significantly lower precision. The consistency of the results in the electron and muon channels provide a further test of the experimental calibrations, whereas the consistency of the results for the different charge and $|\eta _\ell |$ categories tests the *W*-boson production model.

Further consistency tests are performed by repeating the measurement in three intervals of $\left\langle \mu \right\rangle$, in two intervals of $u_{\mathrm {T}}$ and $u_{\parallel }^\ell$, and by removing the $p_{\text {T}}^{\text {miss}}$ selection requirement, which is applied in the nominal signal selection. The consistency of the values of $m_W$ in these additional categories probes the modelling of the recoil response, and the modelling of the transverse-momentum spectrum of the *W* boson. Finally, the stability of the result with respect to the charged-lepton azimuth, and upon variations of the fitting ranges is verified.

Systematic uncertainties in the determination of $m_W$ are evaluated using pseudodata samples produced from the nominal simulated event samples by varying the parameters corresponding to each source of uncertainty in turn. The differences between the values of $m_W$ extracted from the pseudodata and nominal samples are used to estimate the uncertainty. When relevant, these variations are applied simultaneously in the *W*-boson signal samples and in the background contributions. The systematic uncertainties are estimated separately for each source and for fit ranges of $32<p_{\text {T}} ^\ell <45\,\text {GeV}$ and $66<m_{\mathrm {T}}<99\,\text {GeV}$. These fit ranges minimise the total expected measurement uncertainty, and are used for the final result as discussed in Sect. 11.

In Sects. 6, 7, 8, and 10, which discuss the systematic uncertainties of the $m_W$ measurement, the uncertainties are also given for combinations of measurement categories. This provides information showing the reduction of the systematic uncertainty obtained from the measurement categorisation. For these cases, the combined uncertainties are evaluated including only the expected statistical uncertainty in addition to the systematic uncertainty being considered. However, the total measurement uncertainty is estimated by adding all uncertainty contributions in quadrature for each measurement category, and combining the results accounting for correlations across categories.

During the analysis, an unknown offset was added to the value of $m_W$ used to produce the templates. The offset was randomly selected from a uniform distribution in the range $[-100,100]$ $\,\text {MeV}$, and the same value was used for the $W^{+}$ and $W^{-}$ templates. The offset was removed after the $m_W$ measurements performed in all categories were found to be compatible and the analysis procedure was finalised.

## The ATLAS detector 

The ATLAS experiment [46] is a multipurpose particle detector with a forward-backward symmetric cylindrical geometry. It consists of an inner tracking detector surrounded by a thin superconducting solenoid, electromagnetic and hadronic calorimeters, and a muon spectrometer incorporating three large superconducting toroid magnets.

The inner-detector system (ID) is immersed in a 2 T axial magnetic field and provides charged-particle tracking in the range $|\eta | < 2.5$. At small radii, a high-granularity silicon pixel detector covers the vertex region and typically provides three measurements per track. It is followed by the silicon microstrip tracker, which usually provides eight measurement points per track. These silicon detectors are complemented by a gas-filled straw-tube transition radiation tracker, which enables radially extended track reconstruction up to $|\eta | = 2.0$. The transition radiation tracker also provides electron identification information based on the fraction of hits (typically 35 in total) above a higher energy-deposit threshold corresponding to transition radiation.

The calorimeter system covers the pseudorapidity range $|\eta | < 4.9$. Within the region $|\eta |< 3.2$, electromagnetic (EM) calorimetry is provided by high-granularity lead/liquid-argon (LAr) calorimeters, with an additional thin LAr presampler covering $|\eta |<1.8$ to correct for upstream energy-loss fluctuations. The EM calorimeter is divided into a barrel section covering $|\eta |<1.475$ and two endcap sections covering $1.375<|\eta |<3.2$. For $|\eta |<2.5$ it is divided into three layers in depth, which are finely segmented in $\eta$ and $\phi$. Hadronic calorimetry is provided by a steel/scintillator-tile calorimeter, segmented into three barrel structures within $|\eta | < 1.7$ and two copper/LAr hadronic endcap calorimeters covering $1.5<|\eta |<3.2$. The solid-angle coverage is completed with forward copper/LAr and tungsten/LAr calorimeter modules in $3.1<|\eta |<4.9$, optimised for electromagnetic and hadronic measurements, respectively.

The muon spectrometer (MS) comprises separate trigger and high-precision tracking chambers measuring the deflection of muons in a magnetic field generated by superconducting air-core toroids. The precision chamber system covers the region $|\eta | < 2.7$ with three layers of monitored drift tubes, complemented by cathode strip chambers in the forward region. The muon trigger system covers the range $|\eta | < 2.4$ with resistive plate chambers in the barrel, and thin gap chambers in the endcap regions.

A three-level trigger system is used to select events for offline analysis [47]. The level-1 trigger is implemented in hardware and uses a subset of detector information to reduce the event rate to a design value of at most 75 kHz. This is followed by two software-based trigger levels which together reduce the event rate to about 300 Hz.

## Data samples and event simulation 

The data sample used in this analysis consists of *W*- and *Z*-boson candidate events, collected in 2011 with the ATLAS detector in proton–proton collisions at the LHC, at a centre-of-mass energy of $\sqrt{s}=7$ $\text {TeV}$. The sample for the electron channel, with all relevant detector systems operational, corresponds to approximately 4.6 fb$^{-1}$ of integrated luminosity. A smaller integrated luminosity of approximately 4.1 fb$^{-1}$ is used in the muon channel, as part of the data was discarded due to a timing problem in the resistive plate chambers, which affected the muon trigger efficiency. The relative uncertainty of the integrated luminosity is 1.8% [48]. This data set provides approximately 1.4 $\times 10^7$ reconstructed *W*-boson events and 1.8 $\times 10^6$ *Z*-boson events, after all selection criteria have been applied.

The Powheg MC generator [49–51] (v1/r1556) is used for the simulation of the hard-scattering processes of *W*- and *Z*-boson production and decay in the electron, muon, and tau channels, and is interfaced to Pythia 8 (v8.170) for the modelling of the parton shower, hadronisation, and underlying event [52, 53], with parameters set according to the AZNLO tune [44]. The CT10 PDF set [54] is used for the hard-scattering processes, whereas the CTEQ6L1 PDF set [55] is used for the parton shower. In the *Z*-boson samples, the effect of virtual photon production ($\gamma ^*$) and $Z/\gamma ^*$ interference is included. The effect of QED final-state radiation (FSR) is simulated with Photos (v2.154) [56]. Tau lepton decays are handled by Pythia 8, taking into account polarisation effects. An alternative set of samples for *W*- and *Z*-boson production is generated with Powheg interfaced to Herwig (v6.520) for the modelling of the parton shower [57], and to Jimmy (v4.31) for the underlying event [58]. The *W*- and *Z*-boson masses are set to $m_W=80.399\,\text {GeV}$ and $m_Z=91.1875\,\text {GeV}$, respectively. During the analysis, the value of the *W*-boson mass in the $W\rightarrow \ell \nu$ and $W\rightarrow \tau \nu$ samples was blinded using the reweighting procedure described in Sect. 2.

Top-quark pair production and the single-top-quark processes are modelled using the MC@NLO MC generator (v4.01) [59–61], interfaced to Herwig and Jimmy. Gauge-boson pair production (*WW*, *WZ*, *ZZ*) is simulated with Herwig (v6.520). In all the samples, the CT10 PDF set is used. Samples of heavy-flavour multijet events ($pp\rightarrow b\bar{b} +X$ and $pp\rightarrow c \bar{c} +X$) are simulated with Pythia 8 to validate the data-driven methods used to estimate backgrounds with non-prompt leptons in the final state.

Whereas the extraction of $m_W$ is based on the shape of distributions, and is not sensitive to the overall normalisation of the predicted distributions, it is affected by theoretical uncertainties in the relative fractions of background and signal. The *W*- and *Z*-boson event yields are normalised according to their measured cross sections, and uncertainties of 1.8% and 2.3% are assigned to the $W^{+}/Z$ and $W^{-}/Z$ production cross-section ratios, respectively [41]. The $t\bar{t}$ sample is normalised according to its measured cross section [62] with an uncertainty of 3.9%, whereas the cross-section predictions for the single-top production processes of Refs. [63–65] are used for the normalisation of the corresponding sample, with an uncertainty of 7%. The samples of events with massive gauge-boson pair production are normalised to the NLO predictions calculated with MCFM [66], with an uncertainty of 10% to cover the differences to the NNLO predictions [67].

The response of the ATLAS detector is simulated using a program [68] based on Geant 4 [69]. The ID and the MS were simulated assuming an ideal detector geometry; alignment corrections are applied to the data during event reconstruction. The description of the detector material incorporates the results of extensive studies of the electron and photon calibration [39]. The simulated hard-scattering process is overlaid with additional proton–proton interactions, simulated with Pythia 8 (v8.165) using the A2 tune [70]. The distribution of the average number of interactions per bunch crossing $\left\langle \mu \right\rangle$ spans the range 2.5–16.0, with a mean value of approximately 9.0.

Simulation inaccuracies affecting the distributions of the signal, the response of the detector, and the underlying-event modelling, are corrected as described in the following sections. Physics-modelling corrections, such as those affecting the *W*-boson transverse-momentum distribution and the angular decay coefficients, are discussed in Sect. 6. Calibration and detector response corrections are presented in Sects. 7 and 8.

## Particle reconstruction and event selection 

This section describes the reconstruction and identification of electrons and muons, the reconstruction of the recoil, and the requirements used to select *W*- and *Z*-boson candidate events. The recoil provides an event-by-event estimate of the *W*-boson transverse momentum. The reconstructed kinematic properties of the leptons and of the recoil are used to infer the transverse momentum of the neutrino and the transverse-mass kinematic variables.

### Reconstruction of electrons, muons and the recoil

Electron candidates are reconstructed from clusters of energy deposited in the electromagnetic calorimeter and associated with at least one track in the ID [38, 39]. Quality requirements are applied to the associated tracks in order to reject poorly reconstructed charged-particle trajectories. The energy of the electron is reconstructed from the energy collected in calorimeter cells within an area of size $\Delta \eta \times \Delta \phi = 0.075\times 0.175$ in the barrel, and $0.125\times 0.125$ in the endcaps. A multivariate regression algorithm, developed and optimised on simulated events, is used to calibrate the energy reconstruction. The reconstructed electron energy is corrected to account for the energy deposited in front of the calorimeter and outside the cluster, as well as for variations of the energy response as a function of the impact point of the electron in the calorimeter. The energy calibration algorithm takes as inputs the energy collected by each calorimeter layer, including the presampler, the pseudorapidity of the cluster, and the local position of the shower within the cell of the second layer, which corresponds to the cluster centroid. The kinematic properties of the reconstructed electron are inferred from the energy measured in the EM calorimeter, and from the pseudorapidity and azimuth of the associated track. Electron candidates are required to have $p_{\text {T}} > 15\,\text {GeV}$ and $|\eta |<2.4$ and to fulfil a set of tight identification requirements [38]. The pseudorapidity range $1.2<|\eta |<1.82$ is excluded from the measurement, as the amount of passive material in front of the calorimeter and its uncertainty are largest in this region [39], preventing a sufficiently accurate description of non-Gaussian tails in the electron energy response. Additional isolation requirements on the nearby activity in the ID and calorimeter are applied to improve the background rejection. These isolation requirements are implemented by requiring the scalar sum of the $p_{\text {T}}$ of tracks in a cone of size $\Delta R \equiv \sqrt{(\Delta \eta )^2+(\Delta \phi )^2} < 0.4$ around the electron, $p_{\text {T}} ^{e,\text {cone}}$, and the transverse energy deposited in the calorimeter within a cone of size $\Delta R <0.2$ around the electron, $E_\text {T}^\text {cone}$, to be small. The contribution from the electron candidate itself is excluded. The specific criteria are optimised as a function of electron $\eta$ and $p_{\text {T}}$ to have a combined efficiency of about 95% in the simulation for isolated electrons from the decay of a *W* or *Z* boson.

The muon reconstruction is performed independently in the ID and in the MS, and a combined muon candidate is formed from the combination of a MS track with an ID track, based on the statistical combination of the track parameters [40]. The kinematic properties of the reconstructed muon are defined using the ID track parameters alone, which allows a simpler calibration procedure. The loss of resolution is small (10–15%) in the transverse-momentum range relevant for the measurement of the *W*-boson mass. The ID tracks associated with the muons must satisfy quality requirements on the number of hits recorded by each subdetector [40]. In order to reject muons from cosmic rays, the longitudinal coordinate of the point of closest approach of the track to the beamline is required to be within 10 mm of the collision vertex. Muon candidates are required to have $p_{\text {T}} >20\,\text {GeV}$ and $|\eta |<2.4$. Similarly to the electrons, the rejection of multijet background is increased by applying an isolation requirement : the scalar sum of the $p_{\text {T}}$ of tracks in a cone of size $\Delta R < 0.2$ around the muon candidate, $p_{\text {T}} ^{\mu ,\text {cone}}$, is required to be less than 10% of the muon $p_{\text {T}}$.

The recoil, $\vec {u}_{\mathrm {T}}$, is reconstructed from the vector sum of the transverse energy of all clusters measured in the calorimeters, as defined in Sect. 2.1. The ATLAS calorimeters measure energy depositions in the range $|\eta |<4.9$ with a topological clustering algorithm [71], which starts from cells with an energy of at least four times the expected noise from electronics and pile-up. The momentum vector of each cluster is determined by the magnitude and coordinates of the energy deposition. Cluster energies are initially measured assuming that the energy deposition occurs only through electromagnetic interactions, and are then corrected for the different calorimeter responses to hadrons and electromagnetic particles, for losses due to dead material, and for energy which is not captured by the clustering process. The definition of $\vec {u}_{\mathrm {T}}$ and the inferred quantities $p_{\text {T}}^{\text {miss}}$ and $m_{\mathrm {T}}$ do not involve the explicit reconstruction of particle jets, to avoid possible threshold effects.

Clusters located a distance $\Delta R < 0.2$ from the reconstructed electron or muon candidates are not used for the reconstruction of $\vec {u}_{\mathrm {T}}$. This ensures that energy deposits originating from the lepton itself or from accompanying photons (from FSR or Bremsstrahlung) do not contribute to the recoil measurement. The energy of any soft particles removed along with the lepton is compensated for using the total transverse energy measured in a cone of the same size $\Delta R =0.2$, placed at the same absolute pseudorapidity as the lepton with randomly chosen sign, and at different $\phi$. The total transverse momentum measured in this cone is rotated to the position of the lepton and added to $\vec {u}_{\mathrm {T}}$.

### Event selection 

The *W*-boson sample is collected during data-taking with triggers requiring at least one muon candidate with transverse momentum larger than $18\,\text {GeV}$ or at least one electron candidate with transverse momentum larger than $20\,\text {GeV}$. The transverse-momentum requirement for the electron candidate was raised to $22\,\text {GeV}$ in later data-taking periods to cope with the increased instantaneous luminosity delivered by the LHC. Selected events are required to have a reconstructed primary vertex with at least three associated tracks.

*W*-boson candidate events are selected by requiring exactly one reconstructed electron or muon with $p_{\text {T}} ^\ell > 30\,\text {GeV}$. The leptons are required to match the corresponding trigger object. In addition, the reconstructed recoil is required to be $u_{\mathrm {T}}< 30\,\text {GeV}$, the missing transverse momentum $p_{\text {T}}^{\text {miss}} > 30\,\text {GeV}$ and the transverse mass $m_{\mathrm {T}}> 60\,\text {GeV}$. These selection requirements are optimised to reduce the multijet background contribution, and to minimise model uncertainties from *W* bosons produced at high transverse momentum. A total of 5.89 $\times 10^6$ *W*-boson candidate events are selected in the $W\rightarrow e\nu$ channel, and 7.84 $\times 10^6$ events in the $W\rightarrow \mu \nu$ channel.

As mentioned in Sect. 2, *Z*-boson events are extensively used to calibrate the response of the detector to electrons and muons, and to derive recoil corrections. In addition, *Z*-boson events are used to test several aspects of the modelling of vector-boson production. *Z*-boson candidate events are collected with the same trigger selection used for the *W*-boson sample. The analysis selection requires exactly two reconstructed leptons with $p_{\text {T}} ^\ell > 25\,\text {GeV}$, having the same flavour and opposite charges. The events are required to have an invariant mass of the dilepton system in the range $80<m_{\ell \ell }<100\,\text {GeV}$. In both channels, selected leptons are required to be isolated in the same way as in the *W*-boson event selection. In total, 0.58 $\times 10^6$ and 1.23 $\times 10^6$ *Z*-boson candidate events are selected in the electron and muon decay channels, respectively.

## Vector-boson production and decay

Samples of inclusive vector-boson production are produced using the Powheg MC generator interfaced to Pythia 8, henceforth referred to as Powheg+Pythia 8. The *W*- and *Z*-boson samples are reweighted to include the effects of higher-order QCD and electroweak (EW) corrections, as well as the results of fits to measured distributions which improve the agreement of the simulated lepton kinematic distributions with the data. The effect of virtual photon production and $Z/\gamma ^*$ interference is included in both the predictions and the Powheg+Pythia 8 simulated *Z*-boson samples. The reweighting procedure used to include the corrections in the simulated event samples is detailed in Sect. 6.4.

The correction procedure is based on the factorisation of the fully differential leptonic Drell–Yan cross section [31] into four terms:

2 $$\begin{aligned} \frac{\text {d}\sigma }{\text {d}p_1 \, \text {d}p_2}= & {} \left[ \frac{\text {d}\sigma (m)}{\text {d}m}\right] \left[ \frac{\text {d}\sigma (y)}{\text {d}y}\right] \left[ \frac{\text {d}\sigma (p_{\text {T}}, y)}{\text {d}p_{\text {T}} \,\text {d}y} \left( \frac{\text {d}\sigma (y)}{\text {d}y}\right) ^{-1} \right] \nonumber \\&\times \left[ (1{+}\cos ^2\theta ){+}\sum _{i=0}^{7} A_i(p_{\text {T}},y) P_i(\cos \theta , \phi ) \right] ,\nonumber \\ \end{aligned}$$

where $p_1$ and $p_2$ are the lepton and anti-lepton four-momenta; *m*, $p_{\text {T}}$, and *y* are the invariant mass, transverse momentum, and rapidity of the dilepton system; $\theta$ and $\phi$ are the polar angle and azimuth of the lepton^1^ in any given rest frame of the dilepton system; $A_i$ are numerical coefficients, and $P_i$ are spherical harmonics of order zero, one and two.

The differential cross section as a function of the invariant mass, $\text {d}\sigma (m)/\text {d}m$, is modelled with a Breit–Wigner parameterisation according to Eq. (1). In the case of the *Z*-boson samples, the photon propagator is included using the running electromagnetic coupling constant; further electroweak corrections are discussed in Sect. 6.1. The differential cross section as a function of boson rapidity, $\text {d}\sigma (y)/\text {d}y$, and the coefficients $A_i$ are modelled with perturbative QCD fixed-order predictions, as described in Sect. 6.2. The transverse-momentum spectrum at a given rapidity, $\text {d}\sigma (p_{\text {T}},y)/(\text {d}p_{\text {T}} \,\text {d}y) \cdot (\text {d}\sigma (y)/\text {d}y)^{-1}$, is modelled with predictions based on the Pythia  8 MC generator, as discussed in Sect. 6.3. An exhaustive review of available predictions for *W*- and *Z*-boson production at the LHC is given in Ref. [72].

**Table 2:** Impact on the $m_W$ measurement of systematic uncertainties from higher-order electroweak corrections, for the $p_{\text {T}} ^\ell$ and $m_{\mathrm {T}}$ distributions in the electron and muon decay channels

Decay channel	$W \rightarrow e \nu$		$W \rightarrow \mu \nu$	
Kinematic distribution	$p_{\text {T}} ^\ell$	$m_{\mathrm {T}}$	$p_{\text {T}} ^\ell$	$m_{\mathrm {T}}$
$$\delta m_W$$ [MeV]				
FSR (real)	$<0.1$	$<0.1$	$<0.1$	$<0.1$
Pure weak and IFI corrections	3.3	2.5	3.5	2.5
FSR (pair production)	3.6	0.8	4.4	0.8
Total	4.9	2.6	5.6	2.6

Measurements of $W$- and $Z$-boson production are used to validate and constrain the modelling of the fully differential leptonic Drell–Yan cross section. The PDF central values and uncertainties, as well as the modelling of the differential cross section as a function of boson rapidity, are validated by comparing to the 7 $\text {TeV}$ $W$- and $Z$-boson rapidity measurements [41], based on the same data sample. The QCD parameters of the parton shower model were determined by fits to the transverse-momentum distribution of the *Z* boson measured at 7 $\text {TeV}$ [44]. The modelling of the $A_i$ coefficients is validated by comparing the theoretical predictions to the 8 $\text {TeV}$ measurement of the angular coefficients in *Z*-boson decays [42].

### Electroweak corrections and uncertainties 

The dominant source of electroweak corrections to $W$- and $Z$-boson production originates from QED final-state radiation, and is simulated with Photos. The effect of QED initial-state radiation (ISR) is also included through the Pythia 8 parton shower. The uncertainty in the modelling of QED FSR is evaluated by comparing distributions obtained using the default leading-order photon emission matrix elements with predictions obtained using NLO matrix elements, as well as by comparing Photos with an alternative implementation based on the Yennie–Frautschi–Suura formalism [73], which is available in Winhac [74]. The differences are small in both cases, and the associated uncertainty is considered negligible.

Other sources of electroweak corrections are not included in the simulated event samples, and their full effects are considered as systematic uncertainties. They include the interference between ISR and FSR QED corrections (IFI), pure weak corrections due to virtual-loop and box diagrams, and final-state emission of lepton pairs. Complete $O(\alpha )$ electroweak corrections to the $pp\rightarrow W+X$, $W\rightarrow \ell \nu$ process were initially calculated in Refs. [75, 76]. Combined QCD and EW corrections are however necessary to evaluate the effect of the latter in presence of a realistic $p_{\text {T}} ^W$ distribution. Approximate $O(\alpha _{\mathrm s}\alpha )$ corrections including parton shower effects are available from Winhac, Sanc [77] and in the Powheg framework [78–80]. A complete, fixed-order calculation of $O(\alpha _{\mathrm s}\alpha )$ corrections in the resonance region appeared in Ref. [81].

In the present work the effect of the NLO EW corrections are estimated using Winhac, which employs the Pythia 6 MC generator for the simulation of QCD and QED ISR. The corresponding uncertainties are evaluated comparing the final state distributions obtained including QED FSR only with predictions using the complete NLO EW corrections in the $\alpha (0)$ and $G_\mu$ renormalisation schemes [82]. The latter predicts the larger correction and is used to assign the systematic uncertainty.

Final-state lepton pair production, through $\gamma ^*\rightarrow \ell \ell$ radiation, is formally a higher-order correction but constitutes an significant additional source of energy loss for the *W*-boson decay products. This process is not included in the event simulation, and the impact on the determination of $m_W$ is evaluated using Photos and Sanc.

Table 2 summarises the effect of the uncertainties associated with the electroweak corrections on the $m_W$ measurements. All comparisons described above were performed at particle level. The impact is larger for the $p_{\text {T}} ^\ell$ distribution than for the $m_{\mathrm {T}}$ distribution, and similar between the electron and muon decay channels. A detailed evaluation of these uncertainties was performed in Ref. [83] using Powheg [78], and the results are in fair agreement with Table 2. The study of Ref. [83] also compares, at fixed order, the effect of the approximate $O(\alpha _{\mathrm s}\alpha )$ corrections with the full calculation of Ref. [81], and good agreement is found. The same sources of uncertainty affect the lepton momentum calibration through their impact on the $m_{\ell \ell }$ distribution in *Z*-boson events, as discussed in Sect. 7.

### Rapidity distribution and angular coefficients 

At leading order, *W* and *Z* bosons are produced with zero transverse momentum, and the angular distribution of the decay leptons depends solely on the polar angle of the lepton in the boson rest frame. Higher-order corrections give rise to sizeable boson transverse momentum, and to azimuthal asymmetries in the angular distribution of the decay leptons. The angular distribution of the *W*- and *Z*-boson decay leptons is determined by the relative fractions of helicity cross sections for the vector-boson production. The fully differential leptonic Drell–Yan cross section can be decomposed as a weighted sum of nine harmonic polynomials, with weights given by the helicity cross sections. The harmonic polynomials depend on the polar angle, $\theta$, and the azimuth, $\phi$, of the lepton in a given rest frame of the boson. The helicity cross sections depend, in their most general expression, on the transverse momentum, $p_{\text {T}}$, rapidity, *y*, and invariant mass, *m*, of the boson. It is customary to factorise the unpolarised, or angular-integrated, cross section, $\text {d}\sigma /(\text {d}p_{\text {T}}^{2} \, \text {d}y \, \text {d}m)$, and express the decomposition in terms of dimensionless angular coefficients, $A_{i}$, which represent the ratios of the helicity cross sections with respect to the unpolarised cross section [34], leading to the following expression for the fully differential Drell–Yan cross section:

3 $$\begin{aligned} \frac{\text {d}\sigma }{\text {d}p_{\text {T}} ^{2}\, \text {d}y\, \text {d}m\, \text {d}\cos \theta \, \text {d}\phi }= & {} \frac{3}{16\pi }\frac{\text {d}\sigma }{\text {d}p_{\text {T}} ^{2}\, \text {d}y\, \text {d}m} \nonumber \\&\times \left[ (1+\cos ^{2} \theta ) + A_{0} \, \frac{1}{2}(1-3\cos ^{2}\theta ) \right. \nonumber \\&+ A_{1} \, \sin 2\theta \cos \phi + A_{2} \, \frac{1}{2}\sin ^{2}\theta \cos 2\phi \nonumber \\&+ A_{3}\, \sin \theta \cos \phi + A_{4}\, \cos \theta \nonumber \\&+ A_{5}\, \sin ^{2}\theta \sin 2\phi + A_{6}\, \sin 2\theta \sin \phi \nonumber \\&\left. + A_{7}\, \sin \theta \sin \phi \right] . \end{aligned}$$

The angular coefficients depend in general on $p_{\text {T}}$, *y* and *m*. The $A_{5}$–$A_{7}$ coefficients are non-zero only at order $O(\alpha _{\mathrm s}^2)$ and above. They are small in the $p_{\text {T}}$ region relevant for the present analysis, and are not considered further. The angles $\theta$ and $\phi$ are defined in the Collins–Soper (CS) frame [84].

The differential cross section as a function of boson rapidity, $\text {d}\sigma (y)/\text {d}y$, and the angular coefficients, $A_i$, are modelled with fixed-order perturbative QCD predictions, at $O(\alpha _{\mathrm s}^2)$ in the perturbative expansion of the strong coupling constant and using the CT10nnlo PDF set [85]. The dependence of the angular coefficients on *m* is neglected; the effect of this approximation on the measurement of $m_W$ is discussed in Sect. 6.4. For the calculation of the predictions, an optimised version of DYNNLO [86] is used, which explicitly decomposes the calculation of the cross section into the different pieces of the $q_{\mathrm T}$-subtraction formalism, and allows the computation of statistically correlated PDF variations. In this optimised version of DYNNLO, the Cuba library [87] is used for the numerical integration.

**Fig. 1:**
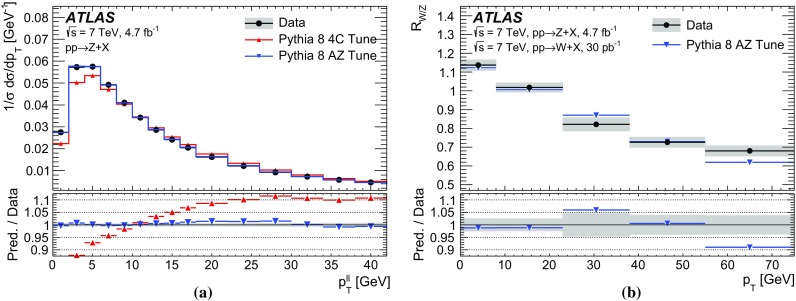
**a** Normalised differential cross section as a function of $p_{\text {T}} ^{\ell \ell }$ in *Z*-boson events [44] and **b** differential cross-section ratio $R_{W/Z}(p_{\text {T}})$ as a function of the boson $p_{\text {T}}$ [44, 45]. The measured cross sections are compared to the predictions of the Pythia 8 AZ tune and, in **a**, of the Pythia 8 4C tune. The shaded bands show the total experimental uncertainties

**Fig. 2:**
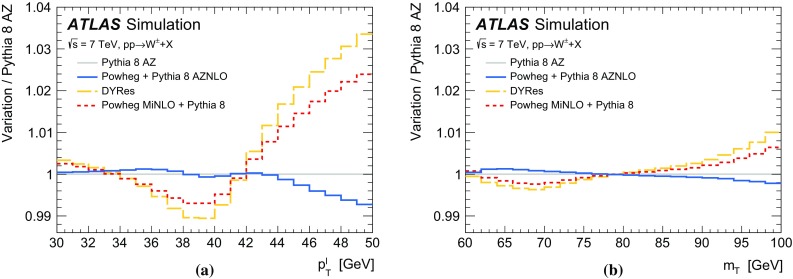
Ratios of the reconstruction-level **a** $p_{\text {T}} ^\ell$ and **b** $m_{\mathrm {T}}$ normalised distributions obtained using Powheg+Pythia 8 AZNLO, DYRes and Powheg MiNLO+Pythia 8 to the baseline normalised distributions obtained using Pythia 8 AZ

The values of the angular coefficients predicted by the Powheg+Pythia 8 samples differ significantly from the corresponding NNLO predictions. In particular, large differences are observed in the predictions of $A_0$ at low values of $p_{\text {T}} ^{W,Z}$. Other coefficients, such as $A_1$ and $A_2$, are affected by significant NNLO corrections at high $p_{\text {T}} ^{W,Z}$. In *Z*-boson production, $A_3$ and $A_4$ are sensitive to the vector couplings between the *Z* boson and the fermions, and are predicted assuming the measured value of the effective weak mixing angle $\sin ^2\theta ^\ell _{\text {eff}}$ [32].

### Transverse-momentum distribution 

Predictions of the vector-boson transverse-momentum spectrum cannot rely solely on fixed-order perturbative QCD. Most $W$-boson events used for the analysis have a low transverse-momentum value, in the kinematic region $p_{\text {T}} ^W < 30\,\text {GeV}$, where large logarithmic terms of the type $\log (m_W/p_{\text {T}} ^W)$ need to be resummed, and non-perturbative effects must be included, either with parton showers or with predictions based on analytic resummation [88–92]. The modelling of the transverse-momentum spectrum of vector bosons at a given rapidity, expressed by the term $\text {d}\sigma (p_{\text {T}},y)/(\text {d}p_{\text {T}} \,\text {d}y) \cdot (\text {d}\sigma (y)/\text {d}y)^{-1}$ in Eq. (2), is based on the Pythia 8 parton shower MC generator. The predictions of vector-boson production in the Pythia 8 MC generator employ leading-order matrix elements for the $q\bar{q}'\rightarrow W, Z$ processes and include a reweighting of the first parton shower emission to the leading-order *V*+jet cross section [93]. The resulting prediction of the boson $p_{\text {T}}$ spectrum is comparable in accuracy to those of an NLO plus parton shower generator setup such as Powheg+Pythia 8, and of resummed predictions at next-to-leading logarithmic order [94].

The values of the QCD parameters used in Pythia 8 were determined from fits to the *Z*-boson transverse momentum distribution measured with the ATLAS detector at a centre-of-mass energy of $\sqrt{s} = 7\,\,\text {TeV}$ [44]. Three QCD parameters were considered in the fit: the intrinsic transverse momentum of the incoming partons, the value of $\alpha _{\mathrm s}(m_Z)$ used for the QCD ISR, and the value of the ISR infrared cut-off. The resulting values of the Pythia  8 parameters constitute the AZ tune. The Pythia 8 AZ prediction was found to provide a satisfactory description of the $p_{\text {T}} ^Z$ distribution as a function of rapidity, contrarily to Powheg+Pythia 8  AZNLO; hence the former is chosen to predict the $p_{\text {T}} ^W$ distribution. The good consistency of the $m_W$ measurement results in $|\eta _\ell |$ categories, presented in Sect. 11, is also a consequence of this choice.

To illustrate the results of the parameters optimisation, the Pythia 8 AZ and 4C [95] predictions of the $p_{\text {T}} ^Z$ distribution are compared in Fig. 1a to the measurement used to determine the AZ tune. Kinematic requirements on the decay leptons are applied according to the experimental acceptance. For further validation, the predicted differential cross-section ratio,

$$\begin{aligned} R_{W/Z}(p_{\text {T}}) = \left( \frac{1}{\sigma _W} \cdot \frac{\text {d}\sigma _W(p_{\text {T}})}{\text {d}p_{\text {T}}}\right) \left( \frac{1}{\sigma _Z} \cdot \frac{\text {d}\sigma _Z(p_{\text {T}})}{\text {d}p_{\text {T}}}\right) ^{-1}, \end{aligned}$$

is compared to the corresponding ratio of ATLAS measurements of vector-boson transverse momentum [44, 45]. The comparison is shown in Fig. 1b, where kinematic requirements on the decay leptons are applied according to the experimental acceptance. The measured $Z$-boson $p_{\text {T}}$ distribution is rebinned to match the coarser bins of the $W$-boson $p_{\text {T}}$ distribution, which was measured using only 30 pb$^{-1}$ of data. The theoretical prediction is in agreement with the experimental measurements for the region with $p_{\text {T}} <30\,\text {GeV}$, which is relevant for the measurement of the *W*-boson mass.

**Fig. 3:**
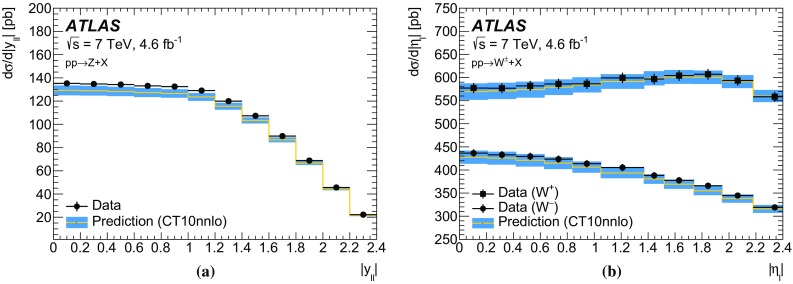
**a** Differential *Z*-boson cross section as a function of boson rapidity, and **b** differential $W^+$ and $W^-$ cross sections as a function of charged decay-lepton pseudorapidity at $\sqrt{s}=7$ $\text {TeV}$ [41]. The measured cross sections are compared to the Powheg+Pythia 8 predictions, corrected to NNLO using DYNNLO with the CT10nnlo PDF set. The error bars show the total experimental uncertainties, including luminosity uncertainty, and the bands show the PDF uncertainties of the predictions

The predictions of RESBOS [89, 90], DYRes [91] and Powheg MiNLO+Pythia 8 [96, 97] are also considered. All predict a harder $p_{\text {T}} ^W$ distribution for a given $p_{\text {T}} ^Z$ distribution, compared to Pythia  8 AZ. Assuming the latter can be adjusted to match the measurement of Ref. [44], the corresponding $p_{\text {T}} ^W$ distribution induces a discrepancy with the detector-level $u_{\text {T}}$ and $u_{\parallel }^\ell$ distributions observed in the *W*-boson data, as discussed in Sect. 11.2. This behaviour is observed using default values for the non-perturbative parameters of these programs, but is not expected to change significantly under variations of these parameters. These predictions are therefore not used in the determination of $m_W$ or its uncertainty.

Figure 2 compares the reconstruction-level $p_{\text {T}} ^\ell$ and $m_{\mathrm {T}}$ distributions obtained with Powheg+Pythia 8 AZNLO, DYRes and Powheg MiNLO+Pythia 8 to those of Pythia  8 AZ.^2^ The effect of varying the $p_{\text {T}} ^W$ distribution is largest at high $p_{\text {T}} ^\ell$, which explains why the uncertainty due to the $p_{\text {T}} ^W$ modelling is reduced when limiting the $p_{\text {T}} ^\ell$ fitting range as described in Sect. 11.3.

### Reweighting procedure

The *W* and *Z* production and decay model described above is applied to the Powheg+Pythia 8 samples through an event-by-event reweighting. Equation (3) expresses the factorisation of the cross section into the three-dimensional boson production phase space, defined by the variables *m*, $p_{\text {T}}$, and *y*, and the two-dimensional boson decay phase space, defined by the variables $\theta$ and $\phi$. Accordingly, a prediction of the kinematic distributions of vector bosons and their decay products can be transformed into another prediction by applying separate reweighting of the three-dimensional boson production phase-space distributions, followed by a reweighting of the angular decay distributions.

**Fig. 4:**
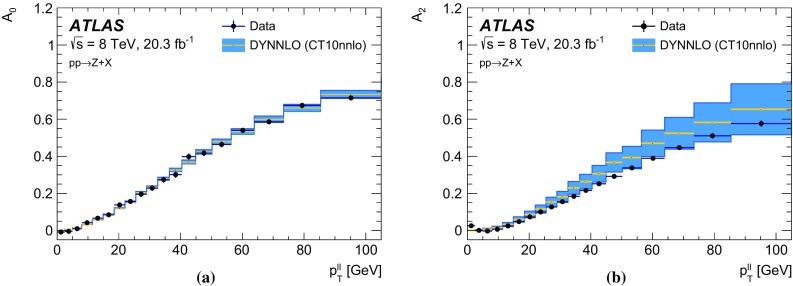
The **a** $A_0$ and **b** $A_2$ angular coefficients in *Z*-boson events as a function of $p_{\text {T}} ^{\ell \ell }$ [42]. The measured coefficients are compared to the DYNNLO predictions using the CT10nnlo PDF set. The error bars show the total experimental uncertainties, and the bands show the uncertainties assigned to the DYNNLO predictions

The reweighting is performed in several steps. First, the inclusive rapidity distribution is reweighted according to the NNLO QCD predictions evaluated with DYNNLO. Then, at a given rapidity, the vector-boson transverse-momentum shape is reweighted to the Pythia 8 prediction with the AZ tune. This procedure provides the transverse-momentum distribution of vector bosons predicted by Pythia 8, preserving the rapidity distribution at NNLO. Finally, at given rapidity and transverse momentum, the angular variables are reweighted according to:

$$\begin{aligned}&w(\cos \theta ,\phi , p_{\text {T}},y) = \frac{1+\cos ^{2}\theta +\sum _i \, A'_i(p_{\text {T}},y) \, P_i(\cos \theta ,\phi )}{1+\cos ^{2}\theta +\sum _i \, A_i(p_{\text {T}},y) \, P_i(\cos \theta ,\phi )}, \end{aligned}$$

where $A'_i$ are the angular coefficients evaluated at $O(\alpha _{\mathrm s}^2)$, and $A_i$ are the angular coefficients of the Powheg+Pythia 8 samples. This reweighting procedure neglects the small dependence of the two-dimensional ($p_{\text {T}}$,*y*) distribution and of the angular coefficients on the final state invariant mass. The procedure is used to include the corrections described in Sects. 6.2 and 6.3, as well as to estimate the impact of the QCD modelling uncertainties described in Sect. 6.5.

**Table 3:** Systematic uncertainties in the $m_W$ measurement due to QCD modelling, for the different kinematic distributions and *W*-boson charges. Except for the case of PDFs, the same uncertainties apply to $W^+$ and $W^-$. The fixed-order PDF uncertainty given for the separate $W^+$ and $W^-$ final states corresponds to the quadrature sum of the CT10nnlo uncertainty variations; the charge-combined uncertainty also contains a $3.8\,\text {MeV}$ contribution from comparing CT10nnlo to CT14 and MMHT2014

*W*-boson charge	$W^+$		$W^-$		Combined	
Kinematic distribution	$p_{\text {T}} ^\ell$	$m_{\mathrm {T}}$	$p_{\text {T}} ^\ell$	$m_{\mathrm {T}}$	$p_{\text {T}} ^\ell$	$m_{\mathrm {T}}$
$$\delta m_W$$ [MeV]						
Fixed-order PDF uncertainty	13.1	14.9	12.0	14.2	8.0	8.7
AZ tune	3.0	3.4	3.0	3.4	3.0	3.4
Charm-quark mass	1.2	1.5	1.2	1.5	1.2	1.5
Parton shower $\mu _\text {F}$ with heavy-flavour decorrelation	5.0	6.9	5.0	6.9	5.0	6.9
Parton shower PDF uncertainty	3.6	4.0	2.6	2.4	1.0	1.6
Angular coefficients	5.8	5.3	5.8	5.3	5.8	5.3
Total	15.9	18.1	14.8	17.2	11.6	12.9

The validity of the reweighting procedure is tested at particle level by generating independent *W*-boson samples using the CT10nnlo and NNPDF3.0 [98] NNLO PDF sets, and the same value of $m_W$. The relevant kinematic distributions are calculated for both samples and used to reweight the CT10nnlo sample to the NNPDF3.0 one. The procedure described in Sect. 2.2 is then used to determine the value of $m_W$ by fitting the NNPDF3.0 sample using templates from the reweighted CT10nnlo sample. The fitted value agrees with the input value within $1.5 \pm 2.0\,\,\text {MeV}$. The statistical precision of this test is used to assign the associated systematic uncertainty.

The resulting model is tested by comparing the predicted *Z*-boson differential cross section as a function of rapidity, the *W*-boson differential cross section as a function of lepton pseudorapidity, and the angular coefficients in *Z*-boson events, to the corresponding ATLAS measurements [41, 42]. The comparison with the measured *W* and *Z* cross sections is shown in Fig. 3. Satisfactory agreement between the measurements and the theoretical predictions is observed. A $\chi ^2$ compatibility test is performed for the three distributions simultaneously, including the correlations between the uncertainties. The compatibility test yields a $\chi ^2/$dof value of 45 / 34. Other NNLO PDF sets such as NNPDF3.0, CT14 [99], MMHT2014 [100], and ABM12 [101] are in worse agreement with these distributions. Based on the quantitative comparisons performed in Ref. [41], only CT10nnlo, CT14 and MMHT2014 are considered further. The better agreement obtained with CT10nnlo can be ascribed to the weaker suppression of the strange quark density compared to the *u*- and *d*-quark sea densities in this PDF set.

The predictions of the angular coefficients in *Z*-boson events are compared to the ATLAS measurement at $\sqrt{s}=8\,\text {TeV}$ [42]. Good agreement between the measurements and DYNNLO is observed for the relevant coefficients, except for $A_2$, where the measurement is significantly below the prediction. As an example, Fig. 4 shows the comparison for $A_0$ and $A_2$ as a function of $p_{\text {T}} ^Z$. For $A_2$, an additional source of uncertainty in the theoretical prediction is considered to account for the observed disagreement with data, as discussed in Sect. 6.5.3.

### Uncertainties in the QCD modelling 

Several sources of uncertainty related to the perturbative and non-perturbative modelling of the strong interaction affect the dynamics of the vector-boson production and decay [33, 102–104]. Their impact on the measurement of $m_W$ is assessed through variations of the model parameters of the predictions for the differential cross sections as functions of the boson rapidity, transverse-momentum spectrum at a given rapidity, and angular coefficients, which correspond to the second, third, and fourth terms of the decomposition of Eq. (2), respectively. The parameter variations used to estimate the uncertainties are propagated to the simulated event samples by means of the reweighting procedure described in Sect. 6.4. Table 3 shows an overview of the uncertainties due to the QCD modelling which are discussed below.

#### Uncertainties in the fixed-order predictions

The imperfect knowledge of the PDFs affects the differential cross section as a function of boson rapidity, the angular coefficients, and the $p_{\text {T}} ^W$ distribution. The PDF contribution to the prediction uncertainty is estimated with the CT10nnlo PDF set by using the Hessian method [105]. There are 25 error eigenvectors, and a pair of PDF variations associated with each eigenvector. Each pair corresponds to positive and negative 90% CL excursions along the corresponding eigenvector. Symmetric PDF uncertainties are defined as the mean value of the absolute positive and negative excursions corresponding to each pair of PDF variations. The overall uncertainty of the CT10nnlo PDF set is scaled to 68% CL by applying a multiplicative factor of 1/1.645.

The effect of PDF variations on the rapidity distributions and angular coefficients are evaluated with DYNNLO, while their impact on the *W*-boson $p_{\text {T}}$ distribution is evaluated using Pythia 8 and by reweighting event-by-event the PDFs of the hard-scattering process, which are convolved with the LO matrix elements. Similarly to other uncertainties which affect the $p_{\text {T}} ^W$ distribution (Sect. 6.5.2), only relative variations of the $p_{\text {T}} ^W$ and $p_{\text {T}} ^Z$ distributions induced by the PDFs are considered. The PDF variations are applied simultaneously to the boson rapidity, angular coefficients, and transverse-momentum distributions, and the overall PDF uncertainty is evaluated with the Hessian method as described above.

Uncertainties in the PDFs are the dominant source of physics-modelling uncertainty, contributing about 14 and $13\,\text {MeV}$ when averaging $p_{\text {T}} ^\ell$ and $m_{\mathrm {T}}$ fits for $W^+$ and $W^-$, respectively. The PDF uncertainties are very similar when using $p_{\text {T}} ^\ell$ or $m_{\mathrm {T}}$ for the measurement. They are strongly anti-correlated between positively and negatively charged *W* bosons, and the uncertainty is reduced to $7.4\,\text {MeV}$ on average for $p_{\text {T}} ^\ell$ and $m_{\mathrm {T}}$ fits, when combining opposite-charge categories. The anti-correlation of the PDF uncertainties is due to the fact that the total light-quark sea PDF is well constrained by deep inelastic scattering data, whereas the *u*-, *d*-, and *s*-quark decomposition of the sea is less precisely known [106]. An increase in the $\bar{u}$ PDF is at the expense of the $\bar{d}$ PDF, which produces opposite effects in the longitudinal polarisation of positively and negatively charged *W* bosons [37].

Other PDF sets are considered as alternative choices. The envelope of values of $m_W$ extracted with the MMHT2014 and CT14 NNLO PDF sets is considered as an additional PDF uncertainty of $3.8\,\text {MeV}$, which is added in quadrature after combining the $W^+$ and $W^-$ categories, leading to overall PDF uncertainties of $8.0\,\text {MeV}$ and $8.7\,\text {MeV}$ for $p_{\text {T}} ^\ell$ and $m_{\mathrm {T}}$ fits, respectively.

The effect of missing higher-order corrections on the NNLO predictions of the rapidity distributions of *Z* bosons, and the pseudorapidity distributions of the decay leptons of *W* bosons, is estimated by varying the renormalisation and factorisation scales by factors of 0.5 and 2.0 with respect to their nominal value $\mu _\text {R} = \mu _\text {F} = m_V$ in the DYNNLO predictions. The corresponding relative uncertainty in the normalised distributions is of the order of 0.1–0.3%, and significantly smaller than the PDF uncertainties. These uncertainties are expected to have a negligible impact on the measurement of $m_W$, and are not considered further.

The effect of the LHC beam-energy uncertainty of 0.65% [107] on the fixed-order predictions is studied. Relative variations of 0.65% around the nominal value of $3.5\,\text {TeV}$ are considered, yielding variations of the inclusive $W^+$ and $W^-$ cross sections of 0.6 and 0.5%, respectively. No significant dependence as a function of lepton pseudorapidity is observed in the kinematic region used for the measurement, and the dependence as a function of $p_{\text {T}} ^\ell$ and $m_{\mathrm {T}}$ is expected to be even smaller. This uncertainty is not considered further.

#### Uncertainties in the parton shower predictions

Several sources of uncertainty affect the Pythia 8 parton shower model used to predict the transverse momentum of the *W* boson. The values of the AZ tune parameters, determined by fits to the measurement of the *Z*-boson transverse momentum, are affected by the experimental uncertainty of the measurement. The corresponding uncertainties are propagated to the $p_{\text {T}} ^W$ predictions through variations of the orthogonal eigenvector components of the parameters error matrix [44]. The resulting uncertainty in $m_W$ is $3.0\,\text {MeV}$ for the $p_{\text {T}} ^\ell$ distribution, and $3.4\,\text {MeV}$ for the $m_{\mathrm {T}}$ distribution. In the present analysis, the impact of $p_{\text {T}} ^W$ distribution uncertainties is in general smaller when using $p_{\text {T}} ^\ell$ than when using $m_{\mathrm {T}}$, as a result of the comparatively narrow range used for the $p_{\text {T}} ^\ell$ distribution fits.

Other uncertainties affecting predictions of the transverse-momentum spectrum of the *W* boson at a given rapidity, are propagated by considering relative variations of the $p_{\text {T}} ^W$ and $p_{\text {T}} ^Z$ distributions. The procedure is based on the assumption that model variations, when applied to $p_{\text {T}} ^Z$, can be largely reabsorbed into new values of the AZ tune parameters fitted to the $p_{\text {T}} ^Z$ data. Variations that cannot be reabsorbed by the fit are excluded, since they would lead to a significant disagreement of the prediction with the measurement of $p_{\text {T}} ^Z$. The uncertainties due to model variations which are largely correlated between $p_{\text {T}} ^W$ and $p_{\text {T}} ^Z$ cancel in this procedure. In contrast, the procedure allows a correct estimation of the uncertainties due to model variations which are uncorrelated between $p_{\text {T}} ^W$ and $p_{\text {T}} ^Z$, and which represent the only relevant sources of theoretical uncertainties in the propagation of the QCD modelling from $p_{\text {T}} ^Z$ to $p_{\text {T}} ^W$.

Uncertainties due to variations of parton shower parameters that are not fitted to the $p_{\text {T}} ^Z$ measurement include variations of the masses of the charm and bottom quarks, and variations of the factorisation scale used for the QCD ISR. The mass of the charm quark is varied in Pythia 8, conservatively, by $\pm \,\, 0.5\,\text {GeV}$ around its nominal value of $1.5\,\text {GeV}$. The resulting uncertainty contributes $1.2\,\text {MeV}$ for the $p_{\text {T}} ^\ell$ fits, and $1.5\,\text {MeV}$ for the $m_{\mathrm {T}}$ fits. The mass of the bottom quark is varied in Pythia 8, conservatively, by $\pm \,\,0.8\,\text {GeV}$ around its nominal value of $4.8\,\text {GeV}$. The resulting variations have a negligible impact on the transverse-momentum distributions of *Z* and *W* bosons, and are not considered further.

The uncertainty due to higher-order QCD corrections to the parton shower is estimated through variations of the factorisation scale, $\mu _\text {F}$, in the QCD ISR by factors of 0.5 and 2.0 with respect to the central choice $\mu _\text {F}^2 = p_{\text {T},0}^2 + p_{\text {T}} ^2$, where $p_{\text {T},0}$ is an infrared cut-off, and $p_{\text {T}}$ is the evolution variable of the parton shower [108]. Variations of the renormalisation scale in the QCD ISR are equivalent to a redefinition of $\alpha _{\mathrm s}(m_Z)$ used for the QCD ISR, which is fixed from the fits to the $p_{\text {T}} ^Z$ data. As a consequence, variations of the ISR renormalisation scale do not apply when estimating the uncertainty in the predicted $p_{\text {T}} ^W$ distribution.

Higher-order QCD corrections are expected to be largely correlated between *W*-boson and *Z*-boson production induced by the light quarks, *u*, *d*, and *s*, in the initial state. However, a certain degree of decorrelation between *W*- and *Z*-boson transverse-momentum distributions is expected, due to the different amounts of heavy-quark-initiated production, where heavy refers to charm and bottom flavours. The physical origin of this decorrelation can be ascribed to the presence of independent QCD scales corresponding to the three-to-four flavours and four-to-five flavours matching scales $\mu _c$ and $\mu _b$ in the variable-flavour-number scheme PDF evolution [109], which are of the order of the charm- and bottom-quark masses, respectively. To assess this effect, the variations of $\mu _\text {F}$ in the QCD ISR are performed simultaneously for all light-quark $q\bar{q} \rightarrow W,Z$ processes, with $q = u,d,s$, but independently for each of the $c\bar{c} \rightarrow Z$, $b\bar{b} \rightarrow Z$, and $c\bar{q} \rightarrow W$ processes, where $q = d,s$. The effect of the $c\bar{q} \rightarrow W$ variations on the determination of $m_W$ is reduced by a factor of two, to account for the presence of only one heavy-flavour quark in the initial state. The resulting uncertainty in $m_W$ is $5.0\,\text {MeV}$ for the $p_{\text {T}} ^\ell$ distribution, and $6.9\,\text {MeV}$ for the $m_{\mathrm {T}}$ distribution. Since the $\mu _\text {F}$ variations affect all the branchings of the shower evolution and not only vertices involving heavy quarks, this procedure is expected to yield a sufficient estimate of the $\mu _{c,b}$-induced decorrelation between the *W*- and *Z*-boson $p_{\text {T}}$ distributions. Treating the $\mu _\text {F}$ variations as correlated between all quark flavours, but uncorrelated between *W*- and *Z*-boson production, would yield a systematic uncertainty in $m_W$ of approximately 30$\,\text {MeV}$.

The predictions of the Pythia 8 MC generator include a reweighting of the first parton shower emission to the leading-order *W*+jet cross section, and do not include matching corrections to the higher-order *W*+jet cross section. As discussed in Sect. 11.2, predictions matched to the NLO *W*+jet cross section, such as Powheg MiNLO+Pythia 8 and DYRes, are in disagreement with the observed $u^\ell _\parallel$ distribution and cannot be used to provide a reliable estimate of the associated uncertainty. The $u^\ell _\parallel$ distribution, on the other hand, validates the Pythia 8 AZ prediction and its uncertainty, which gives confidence that missing higher-order corrections to the *W*-boson $p_{\text {T}}$ distribution are small in comparison to the uncertainties that are already included, and can be neglected at the present level of precision.

The sum in quadrature of the experimental uncertainties of the AZ tune parameters, the variations of the mass of the charm quark, and the factorisation scale variations, leads to uncertainties on $m_W$ of 6.0 and $7.8\,\text {MeV}$ when using the $p_{\text {T}} ^\ell$ distribution and the $m_{\mathrm {T}}$ distribution, respectively. These sources of uncertainty are taken as fully correlated between the electron and muon channels, the positively and negatively charged *W*-boson production, and the $|\eta _\ell |$ bins.

The Pythia 8 parton shower simulation employs the CTEQ6L1 leading-order PDF set. An additional independent source of PDF-induced uncertainty in the $p_{\text {T}} ^W$ distribution is estimated by comparing several choices of the leading-order PDF used in the parton shower, corresponding to the CT14lo, MMHT2014lo and NNPDF2.3lo [110] PDF sets. The PDFs which give the largest deviation from the nominal ratio of the $p_{\text {T}} ^W$ and $p_{\text {T}} ^Z$ distributions are used to estimate the uncertainty. This procedure yields an uncertainty of about $4\,\text {MeV}$ for $W^+$, and of about $2.5\,\text {MeV}$ for $W^-$. Similarly to the case of fixed-order PDF uncertainties, there is a strong anti-correlation between positively and negatively charged *W* bosons, and the uncertainty is reduced to about $1.5\,\text {MeV}$ when combining positive- and negative-charge categories.

The prediction of the $p_{\text {T}} ^W$ distribution relies on the $p_{\text {T}}$-ordered parton shower model of the Pythia 8 MC generator. In order to assess the impact of the choice of parton shower model on the determination of $m_W$, the Pythia 8 prediction of the ratio of the $p_{\text {T}} ^W$ and $p_{\text {T}} ^Z$ distributions is compared to the corresponding prediction of the Herwig 7 MC generator [111, 112], which implements an angular-ordered parton shower model. Differences between the Pythia 8 and Herwig 7 predictions are smaller than the uncertainties in the Pythia 8 prediction, and no additional uncertainty is considered.

#### Uncertainties in the angular coefficients

The full set of angular coefficients can only be measured precisely for the production of *Z* bosons. The accuracy of the NNLO predictions of the angular coefficients is validated by comparison to the *Z*-boson measurement, and extrapolated to *W*-boson production assuming that NNLO predictions have similar accuracy for the *W*- and *Z*-boson processes. The ATLAS measurement of the angular coefficients in *Z*-boson production at a centre-of-mass energy of $\sqrt{s} = 8\,\text {TeV}$ [42] is used for this validation. The $O(\alpha _{\mathrm s}^2)$ predictions, evaluated with DYNNLO, are in agreement with the measurements of the angular coefficients within the experimental uncertainties, except for the measurement of $A_2$ as a function of *Z*-boson $p_{\text {T}}$.

Two sources of uncertainty affecting the modelling of the angular coefficients are considered, and propagated to the *W*-boson predictions. One source is defined from the experimental uncertainty of the *Z*-boson measurement of the angular coefficients which is used to validate the NNLO predictions. The uncertainty in the corresponding *W*-boson predictions is estimated by propagating the experimental uncertainty of the *Z*-boson measurement as follows. A set of pseudodata distributions are obtained by fluctuating the angular coefficients within the experimental uncertainties, preserving the correlations between the different measurement bins for the different coefficients. For each pseudoexperiment, the differences in the $A_i$ coefficients between fluctuated and nominal *Z*-boson measurement results are propagated to the corresponding coefficient in *W*-boson production. The corresponding uncertainty is defined from the standard deviation of the $m_W$ values as estimated from the pseudodata distributions.

The other source of uncertainty is considered to account for the disagreement between the measurement and the NNLO QCD predictions observed for the $A_2$ angular coefficient as a function of the *Z*-boson $p_{\text {T}}$ (Fig. 4). The corresponding uncertainty in $m_W$ is estimated by propagating the difference in $A_2$ between the *Z*-boson measurement and the theoretical prediction to the corresponding coefficient in *W*-boson production. The corresponding uncertainty in the measurement of $m_W$ is $1.6\,\text {MeV}$ for the extraction from the $p_{\text {T}} ^\ell$ distribution. Including this contribution, total uncertainties of 5.8 and $5.3\,\text {MeV}$ due to the modelling of the angular coefficients are estimated in the determination of the *W*-boson mass from the $p_{\text {T}} ^\ell$ and $m_{\mathrm {T}}$ distributions, respectively. The uncertainty is dominated by the experimental uncertainty of the *Z*-boson measurement used to validate the theoretical predictions.

## Calibration of electrons and muons 

Any imperfect calibration of the detector response to electrons and muons impacts the measurement of the *W*-boson mass, as it affects the position and shape of the Jacobian edges reflecting the value of $m_W$. In addition, the $p_{\text {T}} ^\ell$ and $m_{\mathrm {T}}$ distributions are broadened by the electron-energy and muon-momentum resolutions. Finally, the lepton-selection efficiencies depend on the lepton pseudorapidity and transverse momentum, further modifying these distributions. Corrections to the detector response are derived from the data, and presented below. In most cases, the corrections are applied to the simulation, with the exception of the muon sagitta bias corrections and electron energy response corrections, which are applied to the data. Backgrounds to the selected $Z\rightarrow \ell \ell$ samples are taken into account using the same procedures as discussed in Sect. 9. Since the *Z* samples are used separately for momentum calibration and efficiency measurements, as well as for the recoil response corrections discussed in Sect. 8, correlations among the corresponding uncertainties can appear. These correlations were investigated and found to be negligible.

### Muon momentum calibration 

As described in Sect. 5.1, the kinematic parameters of selected muons are determined from the associated inner-detector tracks. The accuracy of the momentum measurement is limited by imperfect knowledge of the detector alignment and resolution, of the magnetic field, and of the amount of passive material in the detector.

Biases in the reconstructed muon track momenta are classified as radial or sagitta biases. The former originate from detector movements along the particle trajectory and can be corrected by an $\eta$-dependent, charge-independent momentum-scale correction. The latter typically originate from curl distortions or linear twists of the detector around the *z*-axis [113], and can be corrected with $\eta$-dependent correction factors proportional to $q\times p_{\text {T}} ^\ell$, where *q* is the charge of the muon. The momentum scale and resolution corrections are applied to the simulation, while the sagitta bias correction is applied to the data:

$$\begin{aligned}&p_{\text {T}} ^{\text {MC,corr}} = p_{\text {T}} ^{\text {MC}} \times \left[ 1 + \alpha (\eta ,\phi )\right] \\&\qquad \qquad \qquad \qquad \times \left[ 1 + \beta _{\text {curv}}(\eta ) \cdot G(0,1) \cdot p_{\text {T}}^{\text {MC}}\right] ,\\ \nonumber&p_{\text {T}} ^{\text {data,corr}} = \frac{p_{\text {T}} ^{\text {data}}}{1 + q \cdot \delta (\eta ,\phi ) \cdot p_{\text {T}} ^{\text {data}}}, \end{aligned}$$

where $p_{\text {T}} ^{\text {data,MC}}$ is the uncorrected muon transverse momentum in data and simulation, *G*(0, 1) are normally distributed random variables with mean zero and unit width, and $\alpha$, $\beta _{\text {curv}}$, and $\delta$ represent the momentum scale, intrinsic resolution and sagitta bias corrections, respectively. Multiple-scattering contributions to the resolution are relevant at low $p_{\text {T}}$, and the corresponding corrections are neglected.

Momentum scale and resolution corrections are derived using $Z\rightarrow \mu \mu$ decays, following the method described in Ref. [40]. Template histograms of the dimuon invariant mass are constructed from the simulated event samples, including momentum scale and resolution corrections in narrow steps within a range covering the expected uncertainty. The optimal values of $\alpha$ and $\beta _{\mathrm {curv}}$ are determined by means of a $\chi ^2$ minimisation, comparing data and simulation in the range of twice the standard deviation on each side of the mean value of the invariant mass distribution. In the first step, the corrections are derived by averaging over $\phi$, and for 24 pseudorapidity bins in the range $-\,2.4< \eta _\ell < 2.4$. In the second iteration, $\phi$-dependent correction factors are evaluated in coarser bins of $\eta _\ell$. The typical size of $\alpha$ varies from − 0.0005 to − 0.0015 depending on $\eta _\ell$, while $\beta _{\text {curv}}$ values increase from $0.2\, \text {TeV}^{-1}$ in the barrel to $0.6\, \text {TeV}^{-1}$ in the high $\eta _\ell$ region. Before the correction, the $\phi$-dependence has an amplitude at the level of 0.1%.

The $\alpha$ and $\beta _{\mathrm {curv}}$ corrections are sensitive to the following aspects of the calibration procedure, which are considered for the systematic uncertainty: the choice of the fitting range, methodological biases, background contributions, theoretical modelling of *Z*-boson production, non-linearity of the corrections, and material distribution in the ID. The uncertainty due to the choice of fitting range is estimated by varying the range by ${\pm }\,10\%$, and repeating the procedure. The uncertainty due to the fit methodology is estimated by comparing the template fit results with an alternative approach, based on an iterative $\chi ^2$ minimisation. Background contributions from gauge-boson pair and top-quark pair production are estimated using the simulation. The uncertainty in these background contributions is evaluated by varying their normalisation within the theoretical uncertainties on the production cross sections. The uncertainty in the theoretical modelling of *Z*-boson production is evaluated by propagating the effect of electroweak corrections to QED FSR, QED radiation of fermion pairs, and other NLO electroweak corrections described in Sect. 6.1. The experimental uncertainty in the value of the *Z*-boson mass used as input is also accounted for. These sources of uncertainty are summed in quadrature, yielding an uncertainty $\delta \alpha$ in the muon momentum scale correction of approximately $0.5 \times 10^{-4}$; these sources are considered fully correlated across muon pseudorapidity.

**Fig. 5:**
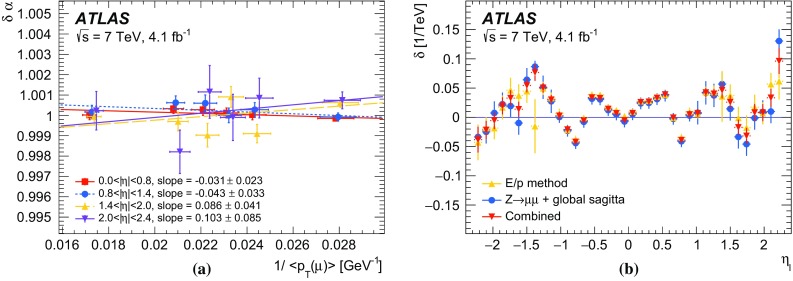
**a** Residual muon momentum scale corrections as a function of muon $1/p_{\text {T}}$ in four pseudorapidity regions, obtained with $Z\rightarrow \mu \mu$ events. The points are fitted using a linear function which parameterises the extrapolation of the muon momentum scale correction from *Z* to *W* events, as explained in the text. The error bars on the points show statistical uncertainties only. **b** Sagitta bias, $\delta$, as a function of $\eta _\ell$ averaged over $\phi _\ell$. The results are obtained with the $Z\rightarrow \mu \mu$ and *E* / *p* methods and the combination of the two. The results obtained with the $Z\rightarrow \mu \mu$ method are corrected for the global sagitta bias. The *E* / *p* method uses electrons from $W\rightarrow e\nu$ decays. The two measurements are combined assuming they are uncorrelated. The error bars on the points show statistical uncertainties only

The systematic uncertainty in the muon momentum scale due to the extrapolation from the $Z\rightarrow \mu \mu$ momentum range to the $W\rightarrow \mu \nu$ momentum range is estimated by evaluating momentum-scale corrections as a function of $1/p_{\text {T}}$ for muons in various $|\eta |$ ranges. The extrapolation uncertainty $\delta \alpha$ is parameterised as follows:where  is the average $p_{\text {T}}$ of muons in *W*-boson events, and $p_0$ and $p_1$ are free parameters. If the momentum-scale corrections are independent of $1/p_{\text {T}}$, the fitting parameters are expected to be $p_0=1$ and $p_1=0$. Deviations of $p_1$ from zero indicate a possible momentum dependence. The fitted values of $\delta \alpha$ are shown in Fig. 5a, and are consistent with one, within two standard deviations of the statistical error. The corresponding systematic uncertainty in $m_W$ is defined assuming, in each bin of $|\eta |$, a momentum non-linearity given by the larger of the fitted value of $p_1$ and its uncertainty. This source of uncertainty is considered uncorrelated across muon pseudorapidity given that $p_1$ is dominated by statistical fluctuations. The effect of the imperfect knowledge of the material in the ID is studied using simulated event samples including an increase of the ID material by 10%, according to the uncertainty estimated in Ref. [114]. The impact of this variation is found to be negligible in comparison with the uncertainties discussed above.

Two methods are used for the determination of the sagitta bias $\delta$. The first method exploits $Z \rightarrow \mu \mu$ events. Muons are categorised according to their charge and pseudorapidity, and for each of these categories, the position of the peak in the dimuon invariant mass distribution is determined for data and simulation. The procedure allows the determination of the charge dependence of the momentum scale for $p_{\mathrm T}$ values of approximately $42\,\text {GeV}$, which corresponds to the average transverse momentum of muons from *Z*-boson decays. The second method exploits identified electrons in a sample of $W\rightarrow e\nu$ decays. It is based on the ratio of the measured electron energy deposited in the calorimeter, *E*, to the electron momentum, *p*, measured in the ID. A clean sample of $W\rightarrow e\nu$ events with tightly identified electrons [38] is selected. Assuming that the response of the electromagnetic calorimeter is independent of the charge of the incoming particle, charge-dependent ID track momentum biases are extracted from the average differences in *E* / *p* for electrons and positrons [113]. This method benefits from a larger event sample compared to the first method, and allows the determination of charge-dependent corrections for $p_{\mathrm T}$ values of approximately $38\,\text {GeV}$, which corresponds to the average transverse momentum of muons in *W*-boson decays. The sagitta bias correction factors are derived using both methods separately in 40 $\eta$ bins and 40 $\phi$ bins. The results are found to agree within uncertainties and are combined, as illustrated in Fig. 5b. The combined correction uncertainty is dominated by the finite size of the event samples.

**Fig. 6:**
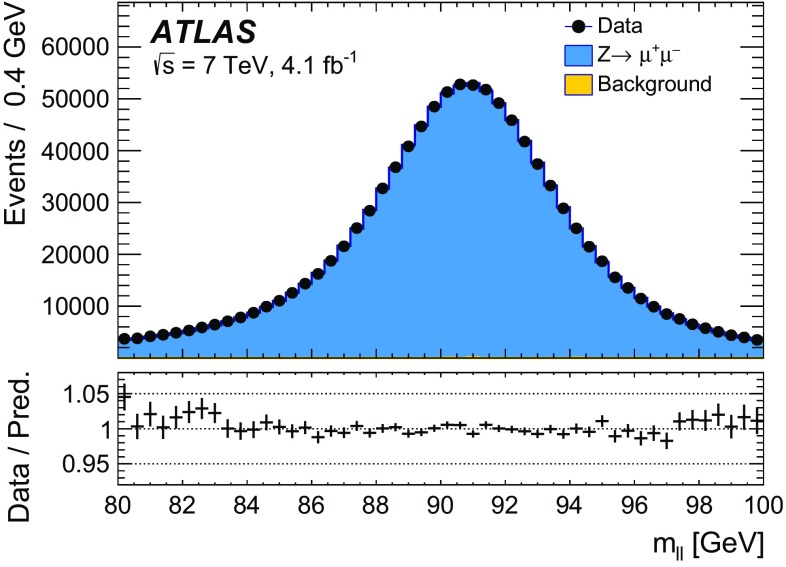
Dimuon invariant mass distribution in $Z\rightarrow \mu \mu$ events. The data are compared to the simulation including signal and background contributions. Corrections for momentum scale and resolution, and for reconstruction, isolation, and trigger efficiencies are applied to the muons in the simulated events. Background events contribute less than 0.2% of the observed distribution. The lower panel shows the data-to-prediction ratio, with the error bars showing the statistical uncertainty

Figure 6 shows the dimuon invariant mass distribution of $Z \rightarrow \mu \mu$ decays in data and simulation, after applying all corrections. Table 4 summarises the effect of the muon momentum scale and resolution uncertainties on the determination of $m_W$. The dominant systematic uncertainty in the momentum scale is due to the extrapolation of the correction from the *Z*-boson momentum range to the *W*-boson momentum range. The extrapolation uncertainty $\delta \alpha$ is (2–$5)\times 10^{-5}$ for $|\eta _\ell |<2.0$, and (4–$7)\times 10^{-4}$ for $|\eta _\ell |>2.0$. Systematic uncertainties from other sources are relatively small. The systematic uncertainty of the resolution corrections is dominated by the statistical uncertainty of the *Z*-boson event sample, and includes a contribution from the imperfect closure of the method. The latter is defined from the residual difference between the standard deviations of the dimuon invariant mass in data and simulation, after applying resolution corrections.

### Muon selection efficiency

The selection of muon candidates in $W\rightarrow \mu \nu$ and $Z\rightarrow \mu \mu$ events requires an isolated track reconstructed in the inner detector and in the muon spectrometer. In addition, the events are required to pass the muon trigger selection. Differences in the efficiency of the reconstruction and selection requirements between data and simulation can introduce a systematic shift in the measurement of the *W*-boson mass, and have to be corrected. In particular, the extraction of $m_W$ is sensitive to the dependence of the trigger, reconstruction and isolation efficiencies on the muon $p_{\text {T}}$ and on the projection of the recoil on the lepton transverse momentum, $u^\ell _\parallel$.

For muons with $p_{\text {T}}$ larger than approximately $15\,\text {GeV}$ the detector simulation predicts constant efficiency as a function of $p_{\text {T}} ^\ell$, both for the muon trigger selection and the track reconstruction. In contrast, the efficiency of the isolation requirement is expected to vary as a function of $p_{\text {T}} ^\ell$ and $u^\ell _\parallel$. The efficiency corrections also affect the muon selection inefficiency, and hence the estimation of the $Z\rightarrow \mu \mu$ background, which contributes to the $W\rightarrow \mu \nu$ selection when one of the decay muons fails the muon reconstruction or kinematic selection requirements.

**Table 4:** Systematic uncertainties in the $m_W$ measurement from muon calibration and efficiency corrections, for the different kinematic distributions and $|\eta _\ell |$ categories, averaged over lepton charge. The momentum-scale uncertainties include the effects of both the momentum scale and linearity corrections. Combined uncertainties are evaluated as described in Sect. 2.2

$|\eta _\ell |$ range	[0.0, 0.8]		[0.8, 1.4]		[1.4, 2.0]		[2.0, 2.4]		Combined	
Kinematic distribution	$p_{\text {T}} ^\ell$	$m_{\mathrm {T}}$	$p_{\text {T}} ^\ell$	$m_{\mathrm {T}}$	$p_{\text {T}} ^\ell$	$m_{\mathrm {T}}$	$p_{\text {T}} ^\ell$	$m_{\mathrm {T}}$	$p_{\text {T}} ^\ell$	$m_{\mathrm {T}}$
$$\delta m_W$$ [MeV]										
Momentum scale	8.9	9.3	14.2	15.6	27.4	29.2	111.0	115.4	8.4	8.8
Momentum resolution	1.8	2.0	1.9	1.7	1.5	2.2	3.4	3.8	1.0	1.2
Sagitta bias	0.7	0.8	1.7	1.7	3.1	3.1	4.5	4.3	0.6	0.6
Reconstruction and isolation efficiencies	4.0	3.6	5.1	3.7	4.7	3.5	6.4	5.5	2.7	2.2
Trigger efficiency	5.6	5.0	7.1	5.0	11.8	9.1	12.1	9.9	4.1	3.2
Total	11.4	11.4	16.9	17.0	30.4	31.0	112.0	116.1	9.8	9.7

**Fig. 7:**
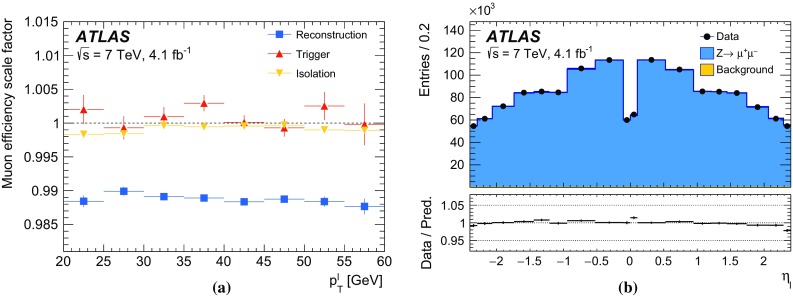
**a** Scale factors for the muon reconstruction, trigger and isolation efficiency obtained with the tag and probe method as a function of the muon $p_{\mathrm T}$. Scale factors for the trigger efficiency are averaged over two data-taking periods as explained in the text. The error bars on the points show statistical uncertainties only. **b** Distribution of the reconstructed muons $\eta$ in $Z \rightarrow \mu \mu$ events. The data are compared to the simulation including signal and background contributions. Corrections for momentum scale and resolution, and for reconstruction, isolation, and trigger efficiencies are applied to the muons in the simulated events. Background events contribute less than 0.2% of the observed distribution. The lower panel shows the data-to-prediction ratio, with the error bars showing the statistical uncertainty

Corrections to the muon reconstruction, trigger and isolation efficiencies are estimated by applying the tag-and-probe method [40] to $Z\rightarrow \mu \mu$ events in data and simulation. Efficiency corrections are defined as the ratio of efficiencies evaluated in data to efficiencies evaluated in simulated events. The corrections are evaluated as functions of two variables, $p_{\text {T}} ^\ell$ and $u^\ell _\parallel$, and in various regions of the detector. The detector is segmented into regions corresponding to the $\eta$ and $\phi$ coverage of the muon spectrometer. The subdivision accounts for the geometrical characteristics of the detector, such as the presence of uninstrumented or transition regions. The dependence of the efficiencies on $u^\ell _\parallel$ agree in data and simulation. Therefore, the muon efficiency corrections are evaluated only as a function of $p_{\text {T}} ^\ell$ and $\eta _\ell$, separately for positive and negative muon charges. The final efficiency correction factors are linearly interpolated as a function of muon $p_{\text {T}}$. No significant $p_{\text {T}}$-dependence of the corrections is observed in any of the detector regions.

The selection of tag-and-probe pairs from $Z\rightarrow \mu \mu$ events is based on the kinematic requirements described in Sect. 5.2. The tag muon is required to be a combined and energy-isolated muon candidate (see Sect. 5.1) which fulfils the muon trigger requirements. The selection requirements applied to the probe muon candidate differ for each efficiency determination: the selection requirement for which the efficiency is determined is removed from the set of requirements applied to the probe muon. All the efficiency corrections are derived inclusively for the full data set, with the exception of the trigger, for which they are derived separately for two different data-taking periods. The resulting scale factors are shown as a function of $p_{\text {T}} ^\ell$ and averaged over $\eta _\ell$ in Fig. 7a. The trigger and isolation efficiency corrections are typically below 0.3%, while the reconstruction efficiency correction is on average about 1.1%. The corresponding impact on muon selection inefficiency reaches up to about 20%.

The quality of the efficiency corrections is evaluated by applying the corrections to the $Z\rightarrow \mu \mu$ simulated sample, and comparing the simulated kinematic distributions to the corresponding distributions in data. Figure 7b illustrates this procedure for the $\eta _\ell$ distribution. Further distributions are shown in Sect. 9.

The dominant source of uncertainty in the determination of the muon efficiency corrections is the statistical uncertainty of the *Z*-boson data sample. The largest sources of systematic uncertainty are the multijet background contribution and the momentum-scale uncertainty. The corresponding uncertainty in the measurement of $m_W$ is approximately 5 $\,\text {MeV}$. The ID tracking efficiencies for muon candidates are above 99.5% without any significant $p_{\mathrm T}$ dependence, and the associated uncertainties are not considered further. An overview of the uncertainties associated with the muon efficiency corrections is shown in Table 4.

### Electron energy response

The electron-energy corrections and uncertainties are largely based on the ATLAS Run 1 electron and photon calibration results [39]. The correction procedure starts with the intercalibration of the first and second layers of the EM calorimeter for minimum-ionising particles, using the energy deposits of muons in $Z\rightarrow \mu \mu$ decays. After the intercalibration of the calorimeter layers, the longitudinal shower-energy profiles of electrons and photons are used to determine the presampler energy scale and probe the passive material in front of the EM calorimeter, leading to an improved description of the detector material distribution and providing estimates of the residual passive material uncertainty. Finally, a dependence of the cell-level energy measurement on the read-out gain is observed in the second layer and corrected for. After these preliminary corrections, an overall energy-scale correction is determined as a function of $\eta _\ell$ from $Z\rightarrow ee$ decays, by comparing the reconstructed mass distributions in data and simulation. Simultaneously, an effective constant term for the calorimeter energy resolution is extracted by adjusting the width of the reconstructed dielectron invariant mass distribution in simulation to match the distribution in data.

Uncertainties in the energy-response corrections arise from the limited size of the $Z\rightarrow ee$ sample, from the physics modelling of the resonance and from the calibration algorithm itself. Physics-modelling uncertainties include uncertainties from missing higher-order electroweak corrections (dominated by the absence of lepton-pair emissions in the simulation) and from the experimental uncertainty in $m_Z$; these effects are taken fully correlated with the muon channel. Background contributions are small and the associated uncertainty is considered to be negligible. Uncertainties related to the calibration procedure are estimated by varying the invariant mass range used for the calibration, and with a closure test. For the closure test, a pseudodata sample of $Z\rightarrow ee$ events is obtained from the nominal sample by rescaling the electron energies by known $\eta$-dependent factors; the calibration algorithm is then applied, and the measured energy corrections are compared with the input rescaling factors.

These sources of uncertainty constitute a subset of those listed in Ref. [39], where additional variations were considered in order to generalise the applicability of the *Z*-boson calibration results to electrons and photons spanning a wide energy range. The effect of these uncertainties is averaged within the different $\eta _\ell$ categories. The overall relative energy-scale uncertainty, averaged over $\eta _\ell$, is $9.4\,\times \,10^{-5}$ for electrons from *Z*-boson decays.

In addition to the uncertainties in the energy-scale corrections arising from the *Z*-boson calibration procedure, possible differences in the energy response between electrons from *Z*-boson and *W*-boson decays constitute a significant source of uncertainty. The linearity of the response is affected by uncertainties in the intercalibration of the layers and in the passive material and calorimeter read-out corrections mentioned above. Additional uncertainties are assigned to cover imperfect electronics pedestal subtraction affecting the energy measurement in the cells of the calorimeter, and to the modelling of the interactions between the electrons and the detector material in Geant4. The contribution from these sources to the relative energy-scale uncertainty is (3–$12)\times 10^{-5}$ in each $\eta$ bin, and $5.4\times 10^{-5}$ when averaged over the full $\eta$ range after taking into account the correlation between the $\eta$ bins.

**Fig. 8:**
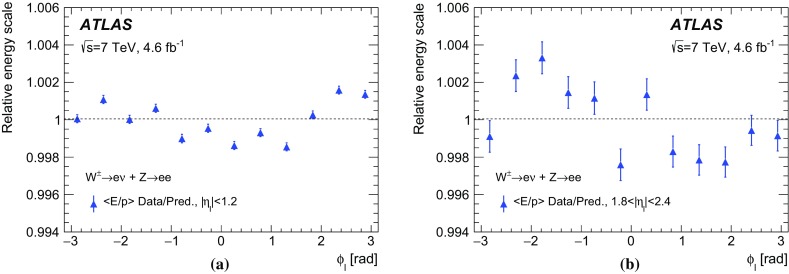
Azimuthal variation of the data-to-prediction ratio of $\left\langle E/p \right\rangle$ in *W* and *Z* events, for electrons in **a** $|\eta _\ell | < 1.2$ and (b) $1.8< |\eta _\ell | < 2.4$. The electron energy calibration based on $Z\rightarrow ee$ events is applied, and the track *p* is corrected for the momentum scale, resolution and sagitta bias. The mean for the *E* / *p* distribution integrated in $\phi$ is normalised to unity. The error bars are statistical only

Azimuthal variations of the electron-energy response are expected from gravity-induced mechanical deformations of the EM calorimeter, and are observed especially in the endcaps, as illustrated in Fig. 8. As the *Z*-boson calibration averages over $\phi _\ell$ and the azimuthal distributions of the selected electrons differ in the two processes, a small residual effect from this modulation is expected when applying the calibration results to the $W\rightarrow e\nu$ sample. Related effects are discussed in Sect. 8. A dedicated correction is derived using the azimuthal dependence of the mean of the electron energy/momentum ratio, $\left\langle E/p \right\rangle$, after correcting *p* for the momentum scale and curvature bias discussed in Sect. 7.1. The effect of this correction is a relative change of the average energy response of $3.8\times 10^{-5}$ in *W*-boson events, with negligible uncertainty.

The *E* / *p* distribution is also used to test the modelling of non-Gaussian tails in the energy response. An excess of events is observed in data at low values of *E* / *p*, and interpreted as the result of the mismodelling of the lateral development of EM showers in the calorimeter. Its impact is evaluated by removing the electrons with *E* / *p* values in the region where the discrepancy is observed. The effect of this removal is compatible for electrons from *W*- and *Z*-boson decays within $4.9\times 10^{-5}$, which corresponds to the statistical uncertainty of the test and is considered as an additional systematic uncertainty.

**Fig. 9:**
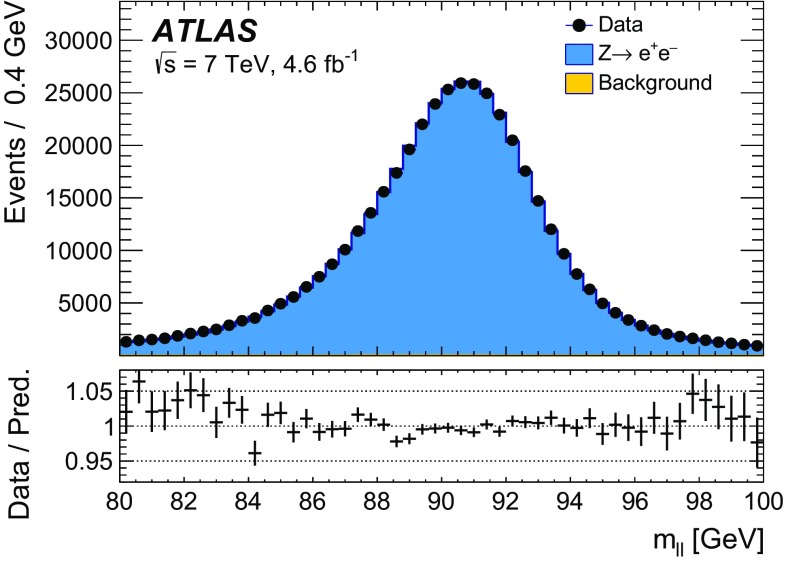
Dielectron invariant mass distribution in $Z \rightarrow ee$ events. The data are compared to the simulation including signal and backgrounds. Corrections for energy resolution, and for reconstruction, identification, isolation and trigger efficiencies are applied to the simulation; energy-scale corrections are applied to the data. Background events contribute less than 0.2% of the observed distribution. The lower panel shows the data-to-prediction ratio, with the error bars showing the statistical uncertainty

The result of the complete calibration procedure is illustrated in Fig. 9, which shows the comparison of the dielectron invariant mass distribution for $Z\rightarrow ee$ events in data and simulation. The impact of the electron-energy calibration uncertainties on the $m_W$ measurement is summarised in Table 5.

### Electron selection efficiency

Electron efficiency corrections are determined using samples of $W\rightarrow e\nu$, $Z\rightarrow ee$, and $J/\psi \rightarrow ee$ events, and measured separately for electron reconstruction, identification and trigger efficiencies [38], as a function of electron $\eta$ and $p_{\text {T}}$. In the $p_{\text {T}}$ range relevant for the measurement of the *W*-boson mass, the reconstruction and identification efficiency corrections have a typical uncertainty of 0.1–0.2% in the barrel, and 0.3% in the endcap. The trigger efficiency corrections have an uncertainty smaller than 0.1%, and are weakly dependent on $p_{\text {T}} ^\ell$.

**Table 5:** Systematic uncertainties in the $m_W$ measurement due to electron energy calibration, efficiency corrections and charge mismeasurement, for the different kinematic distributions and $|\eta _\ell |$ regions, averaged over lepton charge. Combined uncertainties are evaluated as described in Sect. 2.2

$|\eta _\ell |$ range	[0.0, 0.6]		[0.6, 1.2]		[1.8, 2.4]		Combined	
Kinematic distribution	$p_{\text {T}} ^\ell$	$m_{\mathrm {T}}$	$p_{\text {T}} ^\ell$	$m_{\mathrm {T}}$	$p_{\text {T}} ^\ell$	$m_{\mathrm {T}}$	$p_{\text {T}} ^\ell$	$m_{\mathrm {T}}$
$$\delta m_W$$ [MeV]								
Energy scale	10.4	10.3	10.8	10.1	16.1	17.1	8.1	8.0
Energy resolution	5.0	6.0	7.3	6.7	10.4	15.5	3.5	5.5
Energy linearity	2.2	4.2	5.8	8.9	8.6	10.6	3.4	5.5
Energy tails	2.3	3.3	2.3	3.3	2.3	3.3	2.3	3.3
Reconstruction efficiency	10.5	8.8	9.9	7.8	14.5	11.0	7.2	6.0
Identification efficiency	10.4	7.7	11.7	8.8	16.7	12.1	7.3	5.6
Trigger and isolation efficiencies	0.2	0.5	0.3	0.5	2.0	2.2	0.8	0.9
Charge mismeasurement	0.2	0.2	0.2	0.2	1.5	1.5	0.1	0.1
Total	19.0	17.5	21.1	19.4	30.7	30.5	14.2	14.3

For a data-taking period corresponding to approximately 20% of the integrated luminosity, the LAr calorimeter suffered from six front-end board failures. During this period, electrons could not be reconstructed in the region of $0<\eta <1.475$ and $-\,0.9<\phi <-\,0.5$. The data-taking conditions are reflected in the simulation for the corresponding fraction of events. However, the trigger acceptance loss is not perfectly simulated, and dedicated efficiency corrections are derived as a function of $\eta$ and $\phi$ to correct the mismodelling, and applied in addition to the initial corrections.

As described in Sect. 5, isolation requirements are applied to the identified electrons. Their efficiency is approximately 95% in the simulated event samples, and energy-isolation efficiency corrections are derived as for the reconstruction, identification, and trigger efficiencies. The energy-isolation efficiency corrections deviate from unity by less than 0.5%, with an uncertainty smaller than 0.2% on average.

Finally, as positively and negatively charged *W*-boson events have different final-state distributions, the $W^+$ contamination in the $W^-$ sample, and vice versa, constitutes an additional source of uncertainty. The rate of electron charge mismeasurement in simulated events rises from about 0.2% in the barrel to 4% in the endcap. Estimates of charge mismeasurement in data confirm these predictions within better than 0.1%, apart from the high $|\eta |$ region where differences up to 1% are observed. The electron charge mismeasurement induces a systematic uncertainty in $m_W$ of approximately 0.5 $\,\text {MeV}$ in the regions of $|\eta _\ell |<0.6$ and $0.6<|\eta _\ell |<1.2$, and of 5 $\,\text {MeV}$ in the region of $1.8<|\eta _\ell |<2.4$, separately for $W^+$ and $W^-$. Since the $W^+$ and $W^-$ samples contaminate each other, the effect is anti-correlated for the $m_W$ measurements in the two different charge categories, and cancels in their combination, up to the asymmetry in the $W^+/W^-$ production rate. After combination, the residual uncertainty in $m_W$ is 0.2 $\,\text {MeV}$ for $|\eta _\ell |<1.2$, and 1.5$\,\text {MeV}$ for $1.8<|\eta _\ell |<2.4$, for both the $p_{\text {T}} ^\ell$ and $m_{\mathrm {T}}$ distributions. The uncertainties are considered as uncorrelated across pseudorapidity bins.

**Fig. 10:**
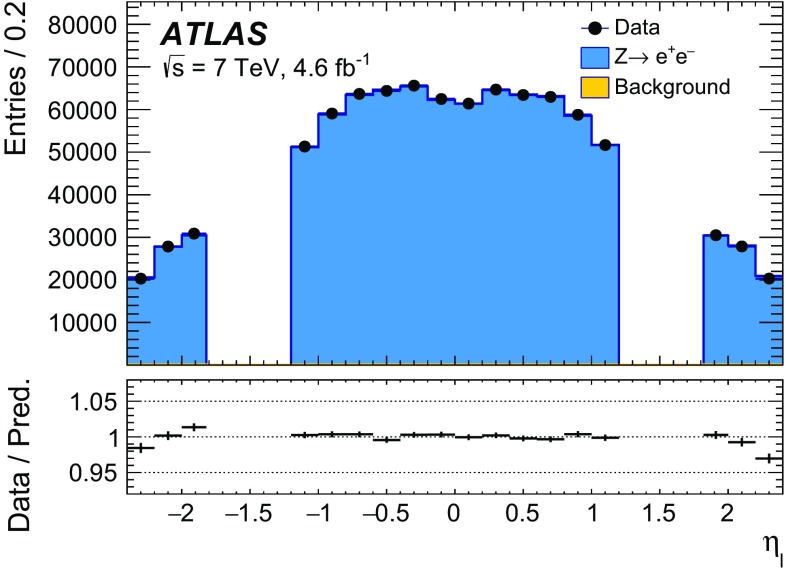
Distribution of reconstructed electrons $\eta$ in $Z \rightarrow ee$ events. The data are compared to the simulation including signal and background contributions. Corrections for energy resolution, and for reconstruction, identification, isolation and trigger efficiencies are applied to the simulation; energy-scale corrections are applied to the data. Background events contribute less than 0.2% of the observed distribution. The lower panel shows the data-to-prediction ratio, with the error bars showing the statistical uncertainty

Figure 10 compares the $\eta _\ell$ distribution in data and simulation for $Z\rightarrow ee$ events, after applying the efficiency corrections discussed above. The corresponding uncertainties in $m_W$ due to the electron efficiency corrections are shown in Table 5.

## Calibration of the recoil 

The calibration of the recoil, $u_{\mathrm {T}}$, affects the measurement of the *W*-boson mass through its impact on the $m_{\mathrm {T}}$ distribution, which is used to extract $m_W$. In addition, the recoil calibration affects the $p_{\text {T}} ^\ell$ and $m_{\mathrm {T}}$ distributions through the $p_{\text {T}}^{\text {miss}}$, $m_{\mathrm {T}}$, and $u_{\mathrm {T}}$ event-selection requirements. The calibration procedure proceeds in two steps. First, the dominant part of the $u_{\mathrm {T}}$ resolution mismodelling is addressed by correcting the modelling of the overall event activity in simulation. These corrections are derived separately in the *W*- and *Z*-boson samples. Second, corrections for residual differences in the recoil response and resolution are derived using *Z*-boson events in data, and transferred to the *W*-boson sample.

**Fig. 11:**
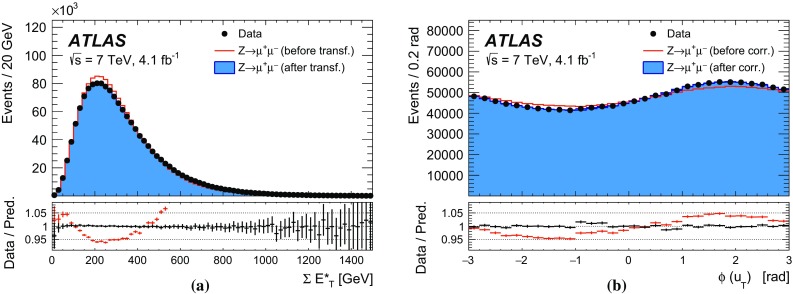
Distributions of **a** $\Sigma E^{*}_{\text {T}}$ and **b** azimuth $\phi$ of the recoil in data and simulation for $Z\rightarrow \mu \mu$ events. The $\Sigma E^{*}_{\text {T}}$ distribution is shown before and after applying the Smirnov-transform correction, and the $\phi$ distribution is shown before and after the $u_{x,y}$ correction. The lower panels show the data-to-prediction ratios, with the vertical bars showing the statistical uncertainty

### Event activity corrections

The pile-up of multiple proton–proton interactions has a significant impact on the resolution of the recoil. As described in Sect. 4, the pile-up is modelled by overlaying the simulated hard-scattering process with additional *pp* interactions simulated using Pythia 8 with the A2 tune. The average number of interactions per bunch crossing is defined, for each event, as $\left\langle \mu \right\rangle =\mathcal {L} \sigma _\text {in}/f_{\text {BC}}$, where $\mathcal {L}$ is the instantaneous luminosity, $\sigma _\text {in}$ is the total *pp* inelastic cross section and $f_{\text {BC}}$ is the average bunch-crossing rate. The distribution of $\left\langle \mu \right\rangle$ in the simulated event samples is reweighted to match the corresponding distribution in data. The distribution of $\left\langle \mu \right\rangle$ is affected in particular by the uncertainty in the cross section and properties of inelastic collisions. In the simulation, $\left\langle \mu \right\rangle$ is scaled by a factor $\alpha$ to optimise the modelling of observed data distributions which are relevant to the modelling of $u_{\mathrm {T}}$. A value of $\alpha =1.10\pm 0.04$ is determined by minimising the $\chi ^2$ function of the compatibility test between data and simulation for the $\Sigma E^{*}_{\text {T}}$ and $u_{\perp }^Z$ distributions, where the uncertainty accounts for differences in the values determined using the two distributions.

After the correction applied to the average number of pile-up interactions, residual data-to-prediction differences in the $\Sigma E^{*}_{\text {T}}$ distribution are responsible for most of the remaining $u_{\mathrm {T}}$ resolution mismodelling. The $\Sigma E^{*}_{\text {T}}$ distribution is corrected by means of a Smirnov transform, which is a mapping $x \rightarrow x'(x)$ such that a function *f*(*x*) is transformed into another target function *g*(*x*) through the relation $f(x) \rightarrow f(x') \equiv g(x)$ [115]. Accordingly, a mapping $\Sigma E^{*}_{\text {T}}\rightarrow \Sigma {E^{*}_{\text {T}}}'$ is defined such that the distribution of $\Sigma E^{*}_{\text {T}}$ in simulation, $h_{\mathrm {MC}}(\Sigma E^{*}_{\text {T}})$, is transformed into $h_{\mathrm {MC}}(\Sigma {E^{*}_{\text {T}}}')$ to match the $\Sigma E^{*}_{\text {T}}$ distribution in data, $h_{\mathrm {data}}(\Sigma E^{*}_{\text {T}})$. The correction is derived for *Z*-boson events in bins of $p_{\mathrm {T}}^{\ell \ell }$, as the observed differences in the $\Sigma E^{*}_{\text {T}}$ distribution depend on the *Z*-boson transverse momentum. The result of this procedure is illustrated in Fig. 11a. The modified distribution is used to parameterise the recoil response corrections discussed in the next section.

In *W*-boson events, the transverse momentum of the boson can only be inferred from $u_{\mathrm {T}}$, which has worse resolution compared to $p_{\mathrm {T}}^{\ell \ell }$ in *Z*-boson events. To overcome this limitation, a $p_{\text {T}}$-dependent correction is defined assuming that the $p_{\text {T}}$ dependence of differences between data and simulation in the $\Sigma E^{*}_{\text {T}}$ distribution in *W*-boson events follows the corresponding differences observed in *Z*-boson events. The $\Sigma E^{*}_{\text {T}}$ distribution to be matched by the simulation is defined as follows for *W*-boson events:

4 $$\begin{aligned}&\tilde{h}^W_{\text {data}}(\Sigma E^{*}_{\text {T}}, p_{\text {T}} ^W)\nonumber \\&\quad \equiv \, h_{\text {data}}^Z(\Sigma E^{*}_{\text {T}}, p_{\text {T}} ^{\ell \ell }) \left( \frac{h^W_{\text {data}}(\Sigma E^{*}_{\text {T}})}{h^W_{\text {MC}}(\Sigma E^{*}_{\text {T}})}\ \Big /\ \frac{h^Z_{\text {data}}(\Sigma E^{*}_{\text {T}})}{h^Z_{\text {MC}}(\Sigma E^{*}_{\text {T}})}\right) , \end{aligned}$$

where $p_{\text {T}} ^W$ is the particle-level *W*-boson transverse momentum, and $p_{\text {T}} ^{\ell \ell }$ the transverse momentum measured from the decay-lepton pair, used as an approximation of the particle-level $p_{\text {T}} ^Z$. The superscripts *W* and *Z* refer to *W*- or *Z*-boson event samples, and the double ratio in the second term accounts for the differences between the inclusive distributions in *W*- and *Z*-boson events. This correction is defined separately for positively and negatively charged *W* bosons, so as to incorporate the dependence of the $p_{\text {T}} ^W$ distribution on the charge of the *W* boson. Using $\tilde{h}^W_{\text {data}}(\Sigma E^{*}_{\text {T}}, p_{\text {T}} ^W)$ defined in Eq. (4) as the target distribution, the $p_{\text {T}} ^W$-dependent Smirnov transform of the $\Sigma E^{*}_{\text {T}}$ distribution in *W*-boson events is defined as follows:

$$\begin{aligned} \nonumber h^W_{\mathrm {MC}}(\Sigma E^{*}_{\text {T}}; p_{\text {T}} ^W) \, \rightarrow \, h_{\mathrm {MC}}^{W}(\Sigma {E^{*}_{\text {T}}}'; p_{\text {T}} ^W) \, \equiv \, \tilde{h}^W_{\mathrm {data}}(\Sigma E^{*}_{\text {T}}; p_{\text {T}} ^W). \end{aligned}$$

The validity of the approximation introduced in Eq. (4) is verified by comparing $h^W_{\text {data}}(\Sigma E^{*}_{\text {T}})/h^W_{\text {MC}}(\Sigma E^{*}_{\text {T}})$ and $h^Z_{\text {data}}(\Sigma E^{*}_{\text {T}})/h^Z_{\text {MC}}(\Sigma E^{*}_{\text {T}})$ in broad bins of $u_{\mathrm {T}}$. The associated systematic uncertainties are discussed in Sect. 8.3.

### Residual response corrections

In the ideal case of beams coinciding with the *z*-axis, the physical transverse momentum of *W* and *Z* bosons is uniformly distributed in $\phi$. However, an offset of the interaction point with respect to the detector centre in the transverse plane, the non-zero crossing angle between the proton beams, and $\phi$-dependent response of the calorimeters generate anisotropies in the reconstructed recoil distribution. Corresponding differences between data and simulation are addressed by effective corrections applied to $u_{x}$ and $u_{y}$ in simulation:where  and  are the mean values of these distributions in data and simulation, respectively. The corrections are evaluated in *Z*-boson events and parameterised as a function of $\Sigma E^{*}_{\text {T}}$. The effect of these corrections on the recoil $\phi$ distribution is illustrated in Fig. 11b.

**Fig. 12:**
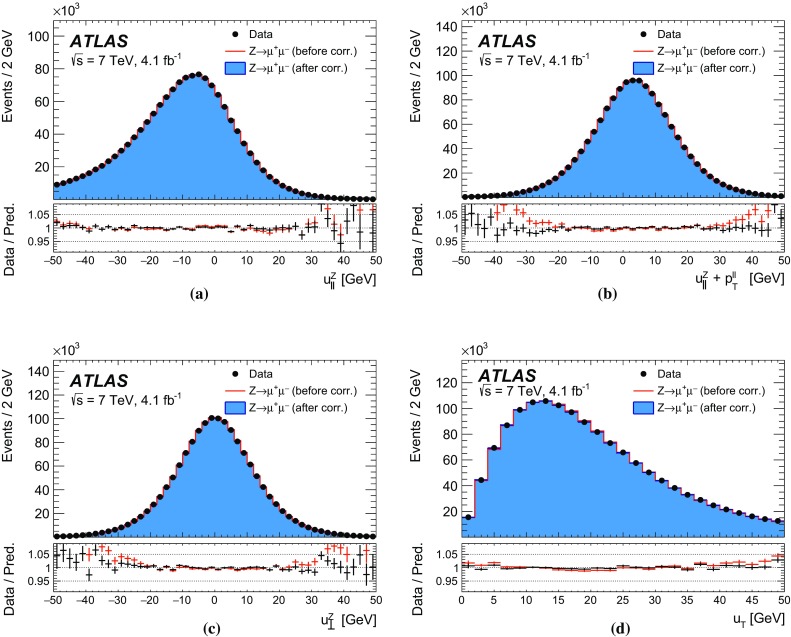
Recoil distributions for **a** $u_{\parallel }^Z$, **b** $u_{\parallel }^Z+p_{\text {T}} ^{\ell \ell }$, (c) $u_{\perp }^Z$, and (d) $u_{\text {T}}$ in $Z \rightarrow \mu \mu$ events. The data are compared to the simulation before and after applying the recoil corrections described in the text. The lower panels show the data-to-prediction ratios, with the vertical bars showing the statistical uncertainty

The transverse momentum of *Z* bosons can be reconstructed from the decay-lepton pair with a resolution of 1–$2\,\text {GeV}$, which is negligible compared to the recoil energy resolution. The recoil response can thus be calibrated from comparisons with the reconstructed $p_{\text {T}} ^{\ell \ell }$ in data and simulation. Recoil energy scale and resolution corrections are derived in bins of $\Sigma E^{*}_{\text {T}}$ and $p_{\text {T}} ^{\ell \ell }$ at reconstruction level, and are applied in simulation as a function of the particle-level vector-boson momentum $p_{\text {T}} ^V$ in both the *W*- and *Z*-boson samples. The energy scale of the recoil is calibrated by comparing the $u_{\parallel }^Z+p_{\text {T}} ^{\ell \ell }$ distribution in data and simulation, whereas resolution corrections are evaluated from the $u_{\perp }^Z$ distribution. Energy-scale corrections $b(p_{\text {T}} ^{V},\Sigma {E^{*}_{\text {T}}}')$ are defined as the difference between the average values of the $u_{\parallel }^Z+p_{\text {T}} ^{\ell \ell }$ distributions in data and simulation, and the energy-resolution correction factors $r(p_{\text {T}} ^{V},\Sigma {E^{*}_{\text {T}}}')$ as the ratio of the standard deviations of the corresponding $u_{\perp }^Z$ distributions.

The parallel component of $u_{\mathrm {T}}$ in simulated events is corrected for energy scale and resolution, whereas the perpendicular component is corrected for energy resolution only. The corrections are defined as follows:

5 $$\begin{aligned} u^{V,\text {corr}}_{\parallel }= & {} \left[ u^{V,\text {MC}}_{\parallel } - \left\langle u^{Z,\text {data}}_{\parallel } \right\rangle \!(p_{\text {T}} ^V,\Sigma {E^{*}_{\text {T}}}')\right] \cdot r(p_{\text {T}} ^V,\Sigma {E^{*}_{\text {T}}}') \, \nonumber \\&+ \left\langle u^{Z,\text {data}}_{\parallel } \right\rangle \!(p_{\text {T}} ^V,\Sigma {E^{*}_{\text {T}}}') + \, b(p_{\text {T}} ^V,\Sigma {E^{*}_{\text {T}}}'), \end{aligned}$$

6 $$\begin{aligned} u^{V,\text {corr}}_{\perp }= & {} u^{V,\text {MC}}_{\perp } \cdot r (p_{\text {T}} ^V,\Sigma {E^{*}_{\text {T}}}'), \end{aligned}$$

where $V=W,Z$, $u^{V,\text {MC}}_{\parallel }$ and $u^{V,\text {MC}}_{\perp }$ are the parallel and perpendicular components of $u_{\mathrm {T}}$ in the simulation, and $u^{V,\text {corr}}_{\parallel }$ and $u^{V,\text {corr}}_{\perp }$ are the corresponding corrected values. As for *b* and *r*, the average $\left\langle u^{Z,\text {data}}_{\parallel } \right\rangle$ is mapped as a function of the reconstructed $p_{\text {T}} ^{\ell \ell }$ in *Z*-boson data, and used as a function of $p_{\text {T}} ^V$ in both *W*- and *Z*-boson simulation. Since the resolution of $u_{\mathrm {T}}$ has a sizeable dependence on the amount of pile-up, the correction procedure is defined in three bins of $\left\langle \mu \right\rangle$, corresponding to low, medium, and high pile-up conditions, and defined by the ranges of $\left\langle \mu \right\rangle \, \in [2.5,6.5]$, $\left\langle \mu \right\rangle \, \in [6.5,9.5]$, and $\left\langle \mu \right\rangle \, \in [9.5,16.0]$, respectively. Values for $b(p_{\text {T}} ^{V},\Sigma {E^{*}_{\text {T}}}')$ are typically $O(100\,\text {MeV})$, and $r(p_{\text {T}} ^{V},\Sigma {E^{*}_{\text {T}}}')$ deviates from unity by 2% at most. The effect of the calibration is shown in Fig. 12 for $Z\rightarrow \mu \mu$ events. The level of agreement obtained after corrections is satisfactory, and similar performance is observed for $Z\rightarrow ee$ events.

**Fig. 13:**
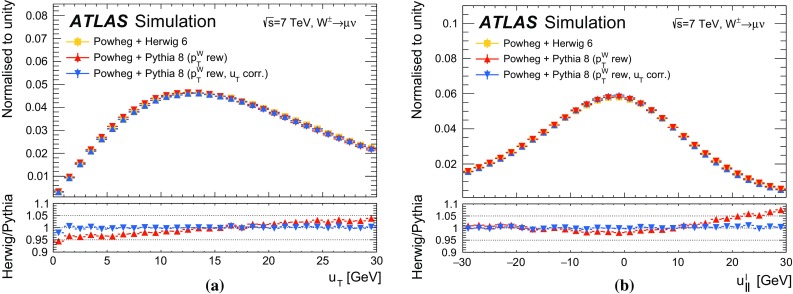
Distributions of **a** $u_{\text {T}}$ and **b** $u_{\parallel }^\ell$ in *W* events simulated using Powheg+Pythia 8 and Powheg+Herwig 6. The recoil response in Powheg+Pythia 8 is corrected to the Powheg+Herwig 6 response using simulated *Z* events following the method described in the text. The $p_{\text {T}} ^W$ distribution in Powheg+Pythia 8 is reweighted to the Powheg+Herwig 6 prediction. The lower panels show the ratios of Powheg+Herwig 6 to Powheg+Pythia 8, with and without the response correction in the Powheg+Pythia 8 sample

A closure test of the applicability of *Z*-based corrections to *W* production is performed using *W* and *Z* samples simulated with Powheg+Herwig 6, which provide an alternative model for the description of hadronisation and the underlying event. The procedure described above is used to correct the recoil response from Powheg+Pythia 8 to Powheg+Herwig 6, where the latter is treated as pseudodata. As shown in Fig. 13, the corrected *W* recoil distributions in Powheg+Pythia 8 match the corresponding distributions in Powheg+Herwig 6. For this study, the effect of the different particle-level $p_{\text {T}} ^W$ distributions in both samples is removed by reweighting the Powheg+Pythia 8 prediction to Powheg+Herwig 6. This study is performed applying the standard lepton selection cuts, but avoiding further kinematic selections in order to maximize the statistics available for the test.

### Systematic uncertainties

The recoil calibration procedure is sensitive to the following sources of systematic uncertainty: the uncertainty of the scale factor applied to the $\left\langle \mu \right\rangle$ distribution, uncertainties due to the Smirnov transform of the $\Sigma {E^{*}_{\text {T}}}$ distribution, uncertainties in the correction of the average value of the $u_{x,y}$ distributions, statistical uncertainties in the residual correction factors and their $p_{\text {T}}$ dependence, and expected differences in the recoil response between *Z*- and *W*-boson events.

The uncertainty from the $\left\langle \mu \right\rangle$ scale-factor $\alpha$ is evaluated by varying it by its uncertainty and repeating all steps of the recoil calibration procedure. These variations affect the determination of $m_W$ by less than $1\,\text {MeV}$.

The systematic uncertainty related to the dependence of the $\Sigma {E^{*}_{\text {T}}}$ correction on $p_{\text {T}}$ is estimated by comparing with the results of a $p_{\text {T}}$-inclusive correction. This source contributes, averaging over *W*-boson charges, an uncertainty of approximately $1\,\text {MeV}$ for the extraction of $m_W$ from the $p_{\text {T}}^\ell$ distribution, and $11\,\text {MeV}$ when using the $m_{\text {T}}$ distribution.

The recoil energy scale and resolution corrections of Eqs. (5) and (6) are derived from the *Z*-boson sample and applied to *W*-boson events. Differences in the detector response to the recoil between *W*- and *Z*-boson processes are considered as a source of systematic uncertainty for these corrections. Differences between the $u_{\perp }^W$ and $u_{\perp }^Z$ distributions originating from different vector-boson kinematic properties, different ISR and FSR photon emission, and from different selection requirements are, however, discarded as they are either accurately modelled in the simulation or already incorporated in the correction procedure.

To remove the effect of such differences, the two-dimensional distribution $h^W_\text {MC}(p_{\text {T}},\Sigma E^{*}_{\text {T}})$ in *W*-boson simulated events is corrected to match the corresponding distribution in *Z*-boson simulated events, treating the neutrinos in *W*-boson decays as charged leptons to calculate $u_{\text {T}}$ as in *Z*-boson events. Finally, events containing a particle-level photon from final-state radiation are removed. After these corrections, the standard deviation of the $u_{\perp }$ distribution agrees within 0.03% between simulated *W*- and *Z*-boson events. This difference is equivalent to 6% of the size of the residual resolution correction, which increases the standard deviation of the $u_{\perp }$ distribution by 0.5%. Accordingly, the corresponding systematic uncertainty due to the extrapolation of the recoil calibration from *Z*- to *W*-boson events is estimated by varying the energy resolution parameter *r* of Eqs. (5) and (6) by 6%. The impact of this uncertainty on the extraction of $m_W$ is approximately $0.2\,\text {MeV}$ for the $p_{\text {T}}^\ell$ distribution, and $5.1\,\text {MeV}$ for the $m_{\text {T}}$ distribution. The extrapolation uncertainty of the energy-scale correction *b* was found to be negligible in comparison.

**Table 6:** Systematic uncertainties in the $m_W$ measurement due to recoil corrections, for the different kinematic distributions and *W*-boson charge categories. Combined uncertainties are evaluated as described in Sect. 2.2

*W*-boson charge	$W^+$		$W^-$		Combined	
Kinematic distribution	$p_{\text {T}} ^\ell$	$m_{\mathrm {T}}$	$p_{\text {T}} ^\ell$	$m_{\mathrm {T}}$	$p_{\text {T}} ^\ell$	$m_{\mathrm {T}}$
$$\delta m_W$$ [MeV]						
$\left\langle \mu \right\rangle$ scale factor	0.2	1.0	0.2	1.0	0.2	1.0
$\Sigma E^{*}_{\text {T}}$ correction	0.9	12.2	1.1	10.2	1.0	11.2
Residual corrections (statistics)	2.0	2.7	2.0	2.7	2.0	2.7
Residual corrections (interpolation)	1.4	3.1	1.4	3.1	1.4	3.1
Residual corrections ($Z\rightarrow W$ extrapolation)	0.2	5.8	0.2	4.3	0.2	5.1
Total	2.6	14.2	2.7	11.8	2.6	13.0

In addition, the statistical uncertainty of the correction factors contributes $2.0\,\text {MeV}$ for the $p_{\text {T}}^\ell$ distribution, and $2.7\,\text {MeV}$ for the $m_{\text {T}}$ distribution. Finally, instead of using a binned correction, a smooth interpolation of the correction values between the bins is performed. Comparing the binned and interpolated correction parameters $b(p_{\text {T}} ^V,\Sigma {E^{*}_{\text {T}}}')$ and $r(p_{\text {T}} ^V,\Sigma {E^{*}_{\text {T}}}')$ leads to a systematic uncertainty in $m_W$ of 1.4 and $3.1\,\text {MeV}$ for the $p_{\text {T}} ^\ell$ and $m_{\mathrm {T}}$ distributions, respectively. Systematic uncertainties in the $u_{x,y}$ corrections are found to be small compared to the other systematic uncertainties, and are neglected.

The impact of the uncertainties of the recoil calibration on the extraction of the *W*-boson mass from the $p_{\text {T}} ^\ell$ and $m_{\mathrm {T}}$ distributions are summarised in Table 6. The determination of $m_W$ from the $p_{\text {T}} ^\ell$ distribution is only slightly affected by the uncertainties of the recoil calibration, whereas larger uncertainties are estimated for the $m_{\mathrm {T}}$ distribution. The largest uncertainties are induced by the $\Sigma {E^{*}_{\text {T}}}$ corrections and by the extrapolation of the recoil energy-scale and energy-resolution corrections from *Z*- to *W*-boson events. The systematic uncertainties are in general smaller for $W^-$ events than for $W^+$ events, as the $\Sigma {E^{*}_{\text {T}}}$ distribution in $W^-$ events is closer to the corresponding distribution in *Z*-boson events.

## Consistency tests with *Z*-boson events 

The $Z\rightarrow \ell \ell$ event sample allows several validation and consistency tests of the *W*-boson analysis to be performed. All the identification requirements of Sect. 5.1, the calibration and efficiency corrections of Sects. 7 and 8, as well as the physics-modelling corrections described in Sect. 6, are applied consistently in the *W*- and *Z*-boson samples. The *Z*-boson sample differs from the *W*-boson sample in the selection requirements, as described in Sect. 5.2. In addition to the event-selection requirements described there, the transverse momentum of the dilepton system, $p_{\text {T}} ^{\ell \ell }$, is required to be smaller than $30\,\text {GeV}$.

**Fig. 14:**
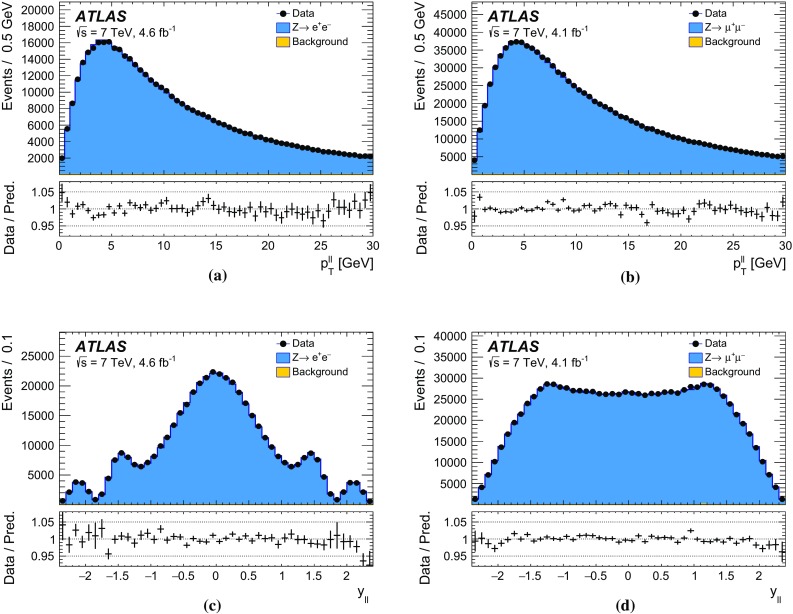
The **a**, **b** $p_{\text {T}} ^{\ell \ell }$ and **c**, **d** $y_{\ell \ell }$ distributions in *Z*-boson events for the **a**, **c** electron and **b**, **d** muon decay channels. The data are compared to the simulation including signal and backgrounds. Detector calibration and physics-modelling corrections are applied to the simulated events. Background events contribute less than 0.2% of the observed distributions. The lower panels show the data-to-prediction ratios, with the error bars showing the statistical uncertainty

The missing transverse momentum in *Z*-boson events is defined by treating one of the two decay leptons as a neutrino and ignoring its transverse momentum when defining the event kinematics. This procedure allows the $p_{\text {T}}^{\text {miss}}$ and $m_{\mathrm {T}}$ variables to be defined in the *Z*-boson sample in close analogy to their definition in the *W*-boson sample. The procedure is repeated, removing the positive and negative lepton in turn.

In the *Z*-boson sample, the background contribution arising from top-quark and electroweak production is estimated using Monte Carlo samples. Each process is normalised using the corresponding theoretical cross sections, evaluated at NNLO in the perturbative expansion of the strong coupling constant. This background contributes a 0.12% fraction in each channel. In the muon channel, the background contribution from multijet events is estimated to be smaller than 0.05% using simulated event samples of $b\bar{b}$ and $c\bar{c}$ production, and neglected. In the electron channel, a data-driven estimate of the multijet background contributes about a 0.1% fraction, before applying the isolation selections, which reduce it to a negligible level.

**Fig. 15:**
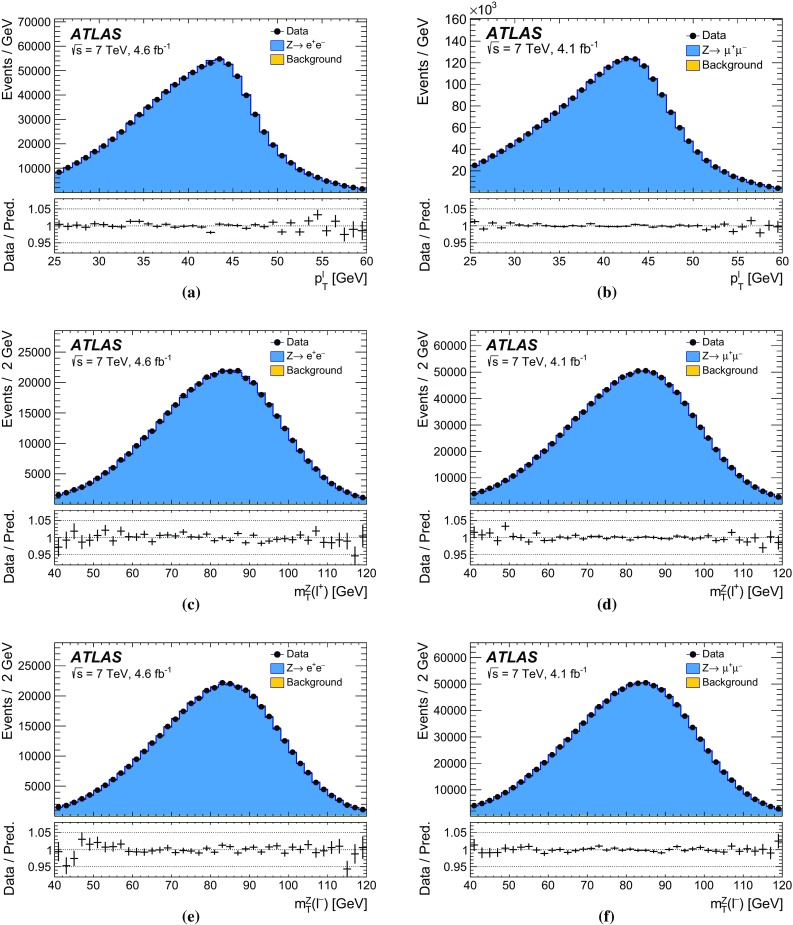
The $p_{\text {T}} ^{\ell }$ distribution in the **a** electron and **b** muon channels, and $m_{\mathrm {T}}$ distributions in the **c**, **e** electron and **d**, **f** muon decay channels for *Z* events when the **c**, **d** negatively charged, or **e**, **f** positively charged lepton is removed. The data are compared to the simulation including signal and backgrounds. Detector calibration and physics-modelling corrections are applied to the simulated events. Background events contribute less than 0.2% of the observed distributions. The lower panels show the data-to-prediction ratios, with the error bars showing the statistical uncertainty

Figure 14 shows the reconstructed distributions of $p_{\text {T}} ^{\ell \ell }$ and $y_{\ell \ell }$ in selected *Z*-boson events; these distributions are not sensitive to the value of $m_Z$. Figure 15 shows the corresponding distributions for $p_{\text {T}} ^{\ell }$ and $m_{\mathrm {T}}$, variables which are sensitive to $m_Z$. Data and simulation agree at the level of 1–2% percent in all the distributions.

The mass of the *Z* boson is extracted with template fits to the $m_{\ell \ell }$, $p_{\text {T}} ^{\ell }$, and $m_{\mathrm {T}}$ kinematic distributions. The extraction of the *Z*-boson mass from the dilepton invariant mass distribution is expected to yield, by construction, the value of $m_Z$ used as input for the muon-momentum and electron-energy calibrations, providing a closure test of the lepton calibration procedures. The $p_{\text {T}} ^\ell$ distribution is very sensitive to the physics-modelling corrections described in Sect. 6. The comparison of the value of $m_Z$ extracted from the $p_{\text {T}} ^\ell$ distribution with the value used as input for the calibration tests the physics modelling and efficiency corrections. Finally, $m_Z$ measurements from the $m_{\mathrm {T}}$ distribution provides a test of the recoil calibration.

**Fig. 16:**
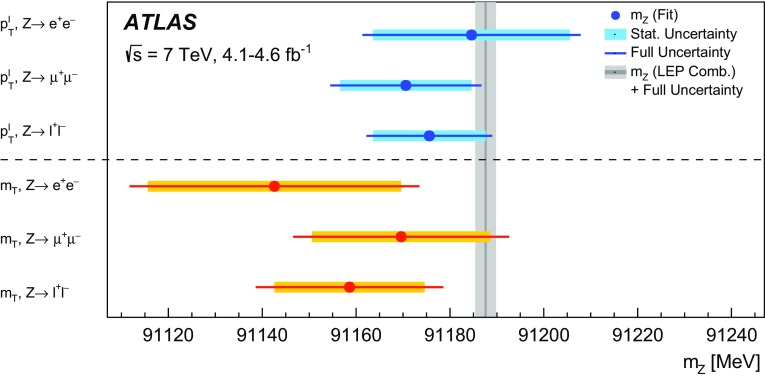
Summary of the $m_Z$ determinations from the $p_{\text {T}} ^\ell$ and $m_{\mathrm {T}}$ distributions in the muon and electron decay channels. The LEP combined value of $m_Z$, which is used as input for the detector calibration, is also indicated. The horizontal and vertical bands show the uncertainties of the $m_Z$ determinations and of the LEP combined value, respectively

**Table 7:** Difference between *Z*-boson mass, extracted from $p_{\text {T}} ^{\ell }$ and $m_{\mathrm {T}}$ distributions, and the LEP combined value. The results are shown separately for the electron and muon decay channels, and their combination. The first quoted uncertainty is statistical, the second is the experimental systematic uncertainty, which includes lepton efficiency and recoil calibration uncertainties where applicable. Physics-modelling uncertainties are neglected

Lepton charge	$\ell ^+$		$\ell ^-$		Combined	
Kinematic distribution	$p_{\text {T}} ^{\ell }$	$m_{\mathrm {T}}$	$p_{\text {T}} ^{\ell }$	$m_{\mathrm {T}}$	$p_{\text {T}} ^{\ell }$	$m_{\mathrm {T}}$
$$\Delta m_Z$$ [MeV]						
$Z \rightarrow ee$	$13 \pm 31\pm 10$	$-\,93 \pm 38\pm 15$	$-\,20 \pm 31\pm 10$	$\, 4 \pm 38\pm 15$	$-\,3 \pm 21\pm 10$	$-\,45 \pm 27\pm 15$
$Z \rightarrow \mu \mu$	$1 \pm 22\pm \;\,8$	$-\,35 \pm 28\pm 13$	$-\,36 \pm 22\pm \;\,8$	$-\,1 \pm 27\pm 13$	$-\,17 \pm 14\pm \;\,8$	$-\,18 \pm 19\pm 13$
Combined	$5 \pm 18\pm \;\,6$	$-\,58 \pm 23\pm 12$	$-\,31 \pm 18\pm \;\,6$	$\,1 \pm 22\pm 12$	$-\,12 \pm 12\pm \;\, 6$	$-\,29 \pm 16\pm 12$

Similarly to the *W*-boson mass, the value of $m_Z$ is determined by minimising the $\chi ^2$ function of the compatibility test between the templates and the measured distributions. The templates are generated with values of $m_Z$ in steps of 4 to $25\,\text {MeV}$ within a range of $\pm \, 450\,\text {MeV}$, centred around a reference value corresponding to the LEP combined value, $m_Z= 91187.5\,\text {MeV}$ [32]. The $\chi ^2$ function is interpolated with a second order polynomial. The minimum of the $\chi ^2$ function yields the extracted value of $m_Z$, and the difference between the extracted value of $m_Z$ and the reference value is defined as $\Delta m_{Z}$. The ranges used for the extraction are $[80, 100]\,\text {GeV}$ for the $m_{\ell \ell }$ distributions, $[30,55]\,\text {GeV}$ for the $p_{\text {T}} ^\ell$ distribution, and $[40,120]\,\text {GeV}$ for the $m_{\mathrm {T}}$ distribution. The extraction of $m_Z$ from the $m_{\mathrm {T}}$ distribution is performed separately for positively and negatively charged leptons in the event, by reconstructing $m_{\mathrm {T}}$ from the kinematic properties of one of the two charged leptons and of the recoil reconstructed by treating the other as a neutrino.

*Z*-boson mass fits are performed using the $m_{\mathrm {T}}$ and $p_{\text {T}} ^\ell$ distributions in the electron and muon decay channels, inclusively in $\eta$ and separately for positively and negatively charged leptons. The results of the fits are summarised in Fig. 16 and Table 7. The $p_{\text {T}} ^\ell$ fit results include all lepton reconstruction systematic uncertainties except the *Z*-based energy or momentum scale calibration uncertainties; the $m_{\mathrm {T}}$ fit results include recoil calibration systematic uncertainties in addition. Physics-modelling uncertainties are neglected.

The value of $m_Z$ measured from positively charged leptons is correlated with the corresponding extraction from the negatively charged leptons. The $p_{\text {T}} ^\ell$ distributions for positively and negatively charged leptons are statistically independent, but the $m_{\mathrm {T}}$ distributions share the same reconstructed recoil event by event, and are statistically correlated. In both cases, the decay of the *Z*-boson induces a kinematical correlation between the distributions of positively and negatively charged leptons. The correlation is estimated by constructing two-dimensional $\ell ^+$ and $\ell ^-$ distributions, separately for $p_{\text {T}} ^{\ell }$ and $m_{\mathrm {T}}$, fluctuating the bin contents of these distributions within their uncertainties, and repeating the fits for each pseudodata sample. The correlation values are $-\,7\%$ for the $p_{\text {T}} ^{\ell }$ distributions, and $-12\%$ for the $m_{\mathrm {T}}$ distributions.

Accounting for the experimental uncertainties as described above, the combined extraction of $m_Z$ from the $p_{\text {T}} ^\ell$ distribution yields a result compatible with the reference value within 0.9 standard deviations. The difference between the $m_Z$ extractions from positively and negatively charged lepton distributions is compatible with zero within 1.4 standard deviations. For the extraction from the $m_{\mathrm {T}}$ distribution, the compatibility with the reference value of $m_Z$ is at the level of 1.5 standard deviations. Fits using the lepton pair invariant mass distribution agree with the reference, yielding $\Delta m_Z = 1\pm 3\,\text {MeV}$ in the muon channel and $\Delta m_Z = 3\pm 5 \,\text {MeV}$ in the electron channel, as expected from the calibration procedure. In summary, the consistency tests based on the *Z*-boson sample agree with the expectations within the experimental uncertainties.

## Backgrounds in the *W*-boson sample 

The *W*-boson event sample, selected as described in Sect. 5.2, includes events from various background processes. Background contributions from *Z*-boson, $W\rightarrow \tau \nu$, boson pair, and top-quark production are estimated using simulation. Contributions from multijet production are estimated with data-driven techniques.

### Electroweak and top-quark backgrounds

The dominant sources of background contribution in the $W\rightarrow \ell \nu$ sample are $Z \rightarrow \ell \ell$ events, in which one of the two leptons escapes detection, and $W\rightarrow \tau \nu$ events, where the $\tau$ decays to an electron or muon. These background contributions are estimated using the Powheg+Pythia 8 samples after applying the modelling corrections discussed in Sect. 6, which include NNLO QCD corrections to the angular coefficients and rapidity distributions, and corrections to the vector-boson transverse momentum. The $Z\rightarrow ee$ background represents 2.9% of the $W^+\rightarrow e\nu$ sample and 4.0% of the $W^-\rightarrow e\nu$ sample. In the muon channel, the $Z\rightarrow \mu \mu$ background represents 4.8 and 6.3% of the $W^+\rightarrow \mu \nu$ and $W^-\rightarrow \mu \nu$ samples, respectively. The $W\rightarrow \tau \nu$ background represents 1.0% of the selected sample in both channels, and the $Z\rightarrow \tau \tau$ background contributes approximately 0.12%. The normalisation of these processes relative to the *W*-boson signal and the corresponding uncertainties are discussed in Sect. 4. A relative uncertainty of 0.2% is assigned to the normalisation of the $W\rightarrow \tau \nu$ samples with respect to the *W*-boson signal sample, to account for the uncertainty in the $\tau$-lepton branching fractions to electrons and muons. In the determination of the *W*-boson mass, the variations of $m_W$ are propagated to the $W\rightarrow \tau \nu$ background templates in the same way as for the signal.

Similarly, backgrounds involving top-quark (top-quark pairs and single top-quark) production, and boson-pair production are estimated using simulation, and normalisation uncertainties are assigned as discussed in Sect. 4. These processes represent 0.11 and 0.07% of the signal event selection, respectively.

Uncertainties in the distributions of the $W\rightarrow \tau \nu$ and $Z\rightarrow \ell \ell$ processes are described by the physics-modelling uncertainties discussed in Sect. 6, and are treated as fully correlated with the signal. Shape uncertainties for boson-pair production and top-quark production are considered negligible compared to the uncertainties in their cross sections, given the small contributions of these processes to the signal event selection.

### Multijet background

Inclusive multijet production in strong-interaction processes constitutes a significant source of background. A fraction of multijet events contains semileptonic decays of bottom and charm hadrons to muons or electrons and neutrinos, and can pass the *W*-boson signal selection. In addition, inclusive jet production contributes to the background if one jet is misidentified as electron or muon, and sizeable missing transverse momentum is reconstructed in the event. In-flight decays of pions or kaons within the tracking region can mimic the *W*-boson signal in the muon channel. In the electron channel, events with photon conversions and hadrons misidentified as electrons can be selected as *W*-boson events. Due to the small selection probability for multijet events, their large production cross section, and the relatively complex modelling of the hadronisation processes, the multijet background contribution cannot be estimated precisely using simulation, and a data-driven method is used instead.

The estimation of the multijet background contribution follows similar procedures in the electron and muon decay channels, and relies on template fits to kinematic distributions in background-dominated regions. The analysis uses the distributions of $p_{\text {T}}^{\text {miss}}$ , $m_{\mathrm {T}}$, and the $p_{\text {T}} ^\ell /m_{\mathrm {T}}$ ratio, where jet-enriched regions are obtained by relaxing a subset of the signal event-selection requirements. The first kinematic region, denoted FR1, is defined by removing the $p_{\text {T}}^{\text {miss}}$ and $m_{\mathrm {T}}$ requirements from the event selection. A second kinematic region, FR2, is defined in the same way as FR1, but by also removing the requirement on $u_{\mathrm {T}}$. Multijet background events, which tend to have smaller values of $p_{\text {T}}^{\text {miss}}$ and $m_{\mathrm {T}}$ than the signal, are enhanced by this selection. The $p_{\text {T}} ^\ell /m_{\mathrm {T}}$ distribution is sensitive to the angle between the $p_{\text {T}} ^{\ell }$ and $p_{\text {T}}^{\text {miss}}$ vectors in the transverse plane. Whereas *W*-boson events are expected to peak at values of $p_{\text {T}} ^\ell /m_{\mathrm {T}}=0.5$, relatively large tails are observed for multijet events.

Templates of the multijet background distributions for these observables are obtained from data by inverting the lepton energy-isolation requirements. Contamination of these control regions by electroweak and top production is estimated using simulation and subtracted. In the muon channel, the anti-isolation requirements are defined from the ratio of the scalar sum of the $p_{\text {T}}$ of tracks in a cone of size $\Delta R < 0.2$ around the reconstructed muon to the muon $p_{\text {T}}$. The isolation variable $p_{\text {T}} ^{\mu ,\text {cone}}$, introduced in Sect. 5.1, is required to satisfy $c_1< p_{\text {T}} ^{\mu ,\text {cone}} / p_{\text {T}} ^\ell < c_2$, where the anti-isolation boundaries $c_1$ and $c_2$ are varied as discussed below. In order to avoid overlap with the signal region, the lower boundary $c_1$ is always larger than 0.1. In the electron channel, the scalar sum of the $p_{\text {T}}$ of tracks in a cone of size $\Delta R < 0.4$ around the reconstructed electron, defined as $p_{\text {T}} ^{e,\text {cone}}$ in Sect. 5.1, is used to define the templates, while the requirements on the calorimeter isolation are omitted.

**Fig. 17:**
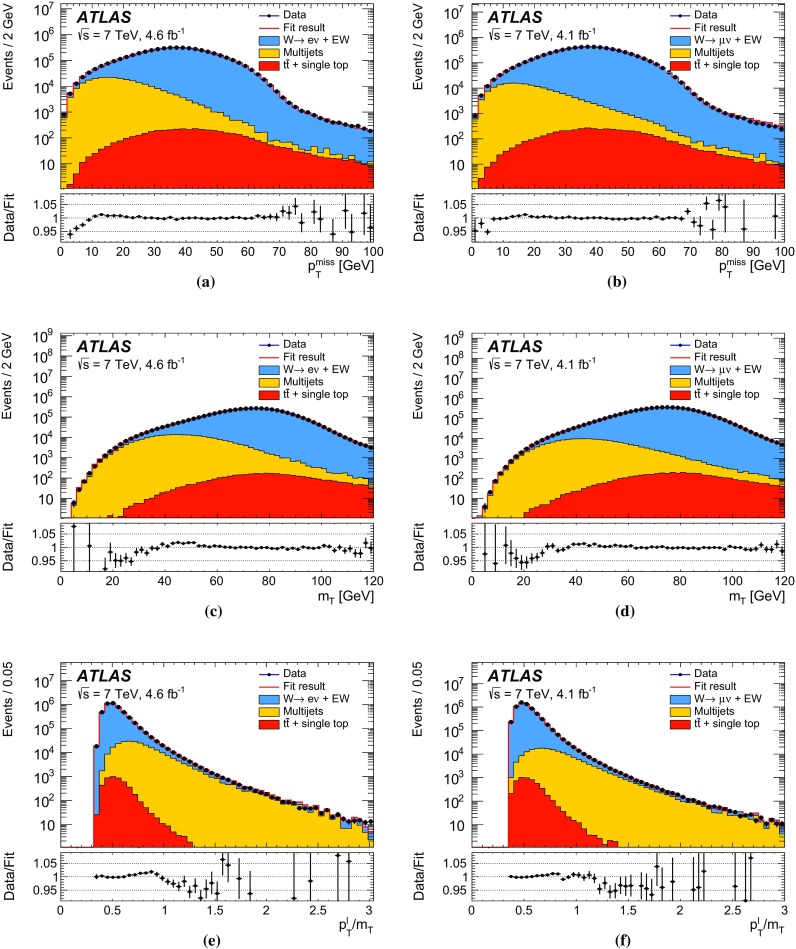
Example template fits to the **a**, **b** $p_{\text {T}}^{\text {miss}}$, **c**, **d** $m_{\mathrm {T}}$, and **e**, **f** $p_{\text {T}} ^\ell /m_{\mathrm {T}}$ distributions in the FR1 kinematic region, in the **a**, **c**, **e** electron and **b**, **d**, **f** muon decay channels. Multijet templates are derived from the data requiring $4\,\text {GeV}<p_{\text {T}} ^{e,\text {cone}}<8\,\text {GeV}$ in the electron channel, and $0.2<p_{\text {T}} ^{\mu ,\text {cone}} / p_{\text {T}} ^\ell <0.4$ in the muon channel. The data are compared to the simulation including signal and background contributions

**Fig. 18:**
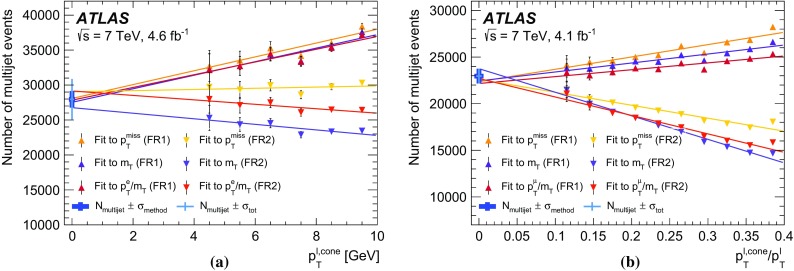
Estimated number of multijet-background events as a function of the lower bound of the isolation-variable range used to define the control regions, for **a** electron and **b** muon decay channel. The estimation is performed for the two regions FR1 and FR2 and three distributions $p_{\text {T}}^{\text {miss}}$, $m_{\mathrm {T}}$, and $p_{\text {T}} ^\ell /m_{\mathrm {T}}$, as described in the text. The linear extrapolations are indicated by the solid lines. The thick crosses show the results of the linear extrapolation of the background estimate to the signal region, including uncertainties from the extrapolation only. The thin crosses also include the uncertainty induced by the contamination of the control regions by EW and top-quark processes

The multijet background normalisation is determined by fitting each of the $p_{\text {T}}^{\text {miss}}$ , $m_{\mathrm {T}}$, and $p_{\text {T}} ^\ell /m_{\mathrm {T}}$ distributions in the two kinematic regions FR1 and FR2, using templates of these distributions based on multijet events and obtained with several ranges of the anti-isolation variables. The multijet background in the signal region is determined by correcting the multijet fraction fitted in the FR1 and FR2 for the different efficiencies of the selection requirements of the signal region. In the electron channel, $c_1$ is varied from 4 to $9\,\text {GeV}$ in steps of $1\,\text {GeV}$, and $c_2$ is set to $c_2 = c_1+1\,\text {GeV}$. In the muon channel, $c_1$ is varied from 0.1 to 0.37 in steps of 0.03, and $c_2$ is set to $c_2=c_1+0.03$. Example results of template fits in the electron and muon channels are shown in Fig. 17. The results corresponding to the various observables and to the different kinematic regions are linearly extrapolated in the isolation variables to the signal regions, denoted by $c_1 = 0$. Figure 18 illustrates the extrapolation procedure.

The systematic uncertainty in the multijet background fraction is defined as half of the largest difference between the results extrapolated from the different kinematic regions and observables. The multijet background contribution is estimated separately in all measurement categories. In the electron channel, the multijet background fraction rises from $0.58\pm 0.08\%$ at low $|\eta _\ell |$ to 1.73$\,\pm \,$0.19% in the last measurement bin, averaging the $W^+$ and $W^-$ channels. In the muon channel, the charge-averaged multijet background fraction decreases from $0.72\pm 0.07\%$ to $0.49\pm 0.03$%, when going from low to high $|\eta _\ell |$. The uncertainties in the multijet background fractions are sufficient to account for the observed residual discrepancies between the fitted distributions and the data (see Fig. 17). The estimated multijet background yields are consistent between $W^+$ and $W^-$, but the multijet background fraction is smaller in the $W^+$ channels due to the higher signal yield.

Corrections to the shape of the multijet background contributions and corresponding uncertainties in the distributions used to measure the *W*-boson mass are estimated with a similar procedure. The kinematic distributions in the control regions are obtained for a set of anti-isolation ranges, and parameterised with linear functions of the lower bound of the anti-isolation requirement. The distributions are extrapolated to the signal regions accordingly. Uncertainties in the extrapolated distributions are dominated by the statistical uncertainty, which is determined with a toy MC method by fluctuating within their statistical uncertainty the bin contents of the histograms in the various anti-isolation ranges. The resulting multijet background distribution is propagated to the templates, and the standard deviation of the determined values of $m_W$ yields the estimated uncertainty due to the shape of the multijet background. Uncertainties due to the choice of parameterisation are small in comparison and neglected.

Uncertainties in the normalisation of multijet, electroweak, and top-quark background processes are considered correlated across decay channels, boson charges and rapidity bins, whereas the uncertainty in the shape of multijet background is considered uncorrelated between decay channels and boson charges. The impact of the background systematic uncertainties on the determination of $m_W$ is summarised in Table 8.

**Table 8:** Systematic uncertainties in the $m_W$ measurement due to electroweak, top-quark, and multijet background estimation, for fits to the $p_\text {T}^\ell$ and $m_{\mathrm {T}}$ distributions, in the electron and muon decay channels, with positively and negatively charged *W* bosons

Kinematic distribution	$p_{\text {T}} ^\ell$				$m_{\mathrm {T}}$			
Decay channel	$W\rightarrow e\nu$		$W\rightarrow \mu \nu$		$W\rightarrow e\nu$		$W\rightarrow \mu \nu$	
*W*-boson charge	$W^+$	$W^-$	$W^+$	$W^-$	$W^+$	$W^-$	$W^+$	$W^-$
$\delta m_W$ [$\,\text {MeV}$]								
$W\rightarrow \tau \nu$ (fraction, shape)	0.1	0.1	0.1	0.2	0.1	0.2	0.1	0.3
$Z\rightarrow ee$ (fraction, shape)	3.3	4.8	–	–	4.3	6.4	–	–
$Z\rightarrow \mu \mu$ (fraction, shape)	–	–	3.5	4.5	–	–	4.3	5.2
$Z\rightarrow \tau \tau$ (fraction, shape)	0.1	0.1	0.1	0.2	0.1	0.2	0.1	0.3
*WW*, *WZ*, *ZZ* (fraction)	0.1	0.1	0.1	0.1	0.4	0.4	0.3	0.4
Top (fraction)	0.1	0.1	0.1	0.1	0.3	0.3	0.3	0.3
Multijet (fraction)	3.2	3.6	1.8	2.4	8.1	8.6	3.7	4.6
Multijet (shape)	3.8	3.1	1.6	1.5	8.6	8.0	2.5	2.4
Total	6.0	6.8	4.3	5.3	12.6	13.4	6.2	7.4

## Measurement of the *W*-boson mass 

This section presents the determination of the mass of the *W* boson from template fits to the kinematic distributions of the *W*-boson decay products. The final measured value is obtained from the combination of measurements performed using the lepton transverse momentum and transverse mass distributions in categories corresponding to the electron and muon decay channels, positively and negatively charged *W* bosons, and absolute pseudorapidity bins of the charged lepton, as illustrated in Table 1. The number of selected events in each category is shown in Table 9.

**Table 9:** Numbers of selected $W^+$ and $W^-$ events in the different decay channels in data, inclusively and for the various $|\eta _\ell |$ categories

$|\eta _\ell |$ range	0–0.8	0.8–1.4	1.4–2.0	2.0–2.4	Inclusive
$W^+\rightarrow \mu ^+\nu$	1 283 332	1 063 131	1 377 773	885 582	4 609 818
$W^-\rightarrow \mu ^-\bar{\nu }$	1 001 592	769 876	916 163	547 329	3 234 960

$|\eta _\ell |$ range	0–0.6	0.6–1.2		1.8–2.4	Inclusive
$W^+\rightarrow e^+\nu$	1 233 960	1 207 136		956 620	3 397 716
$W^-\rightarrow e^-\bar{\nu }$	969 170	908 327		610 028	2 487 525

### Control distributions 

**Fig. 19:**
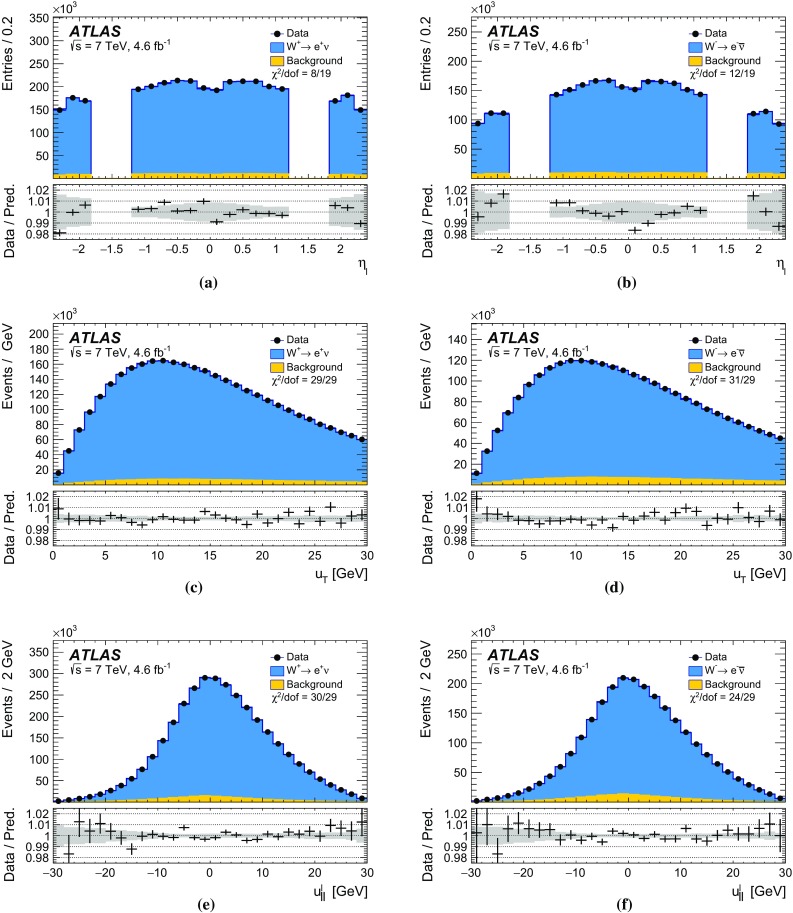
The **a**, **b** $\eta _\ell$, (c,d) $u_{\mathrm {T}}$, and **e**, **f** $u_\parallel ^\ell$ distributions for **a**, **c**, **e** $W^+$ events and **b**, **d**, **f** $W^-$ events in the electron decay channel. The data are compared to the simulation including signal and background contributions. Detector calibration and physics-modelling corrections are applied to the simulated events. The lower panels show the data-to-prediction ratios, the error bars show the statistical uncertainty, and the band shows the systematic uncertainty of the prediction. The $\chi ^2$ values displayed in each figure account for all sources of uncertainty and include the effects of bin-to-bin correlations induced by the systematic uncertainties

**Fig. 20:**
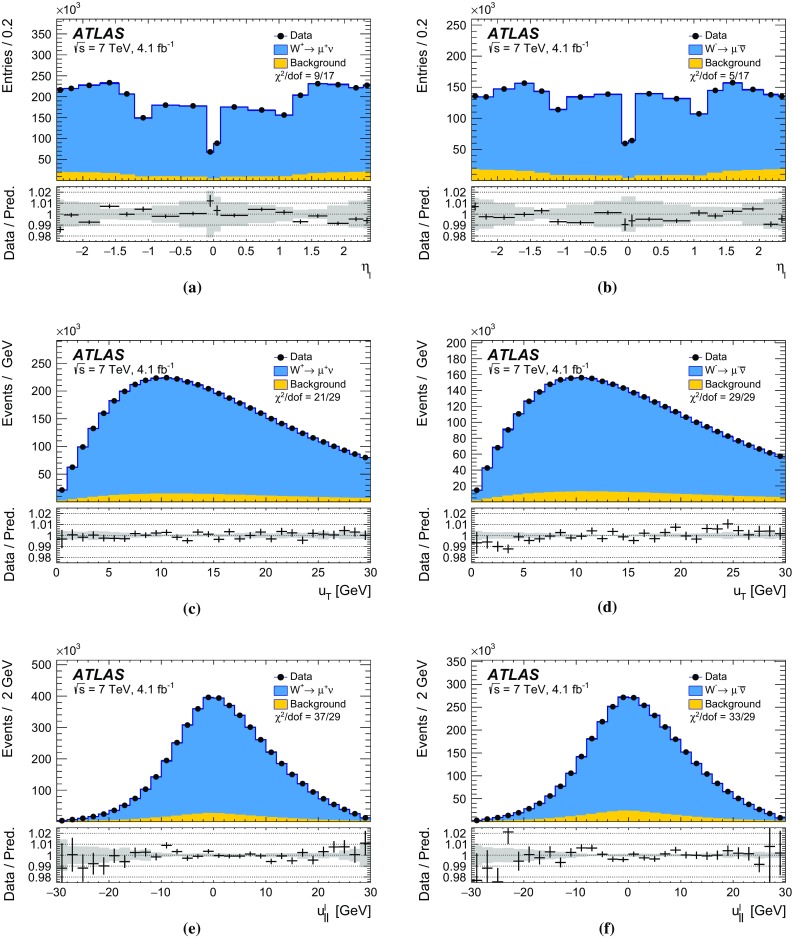
The **a**, **b** $\eta _\ell$, (c,d) $u_{\mathrm {T}}$, and **e**, **f** $u_\parallel ^\ell$ distributions for **a**, **c**, **e** $W^+$ events and **b**, **d**, **f** $W^-$ events in the muon decay channel. The data are compared to the simulation including signal and background contributions. Detector calibration and physics-modelling corrections are applied to the simulated events. The lower panels show the data-to-prediction ratios, the error bars show the statistical uncertainty, and the band shows the systematic uncertainty of the prediction. The $\chi ^2$ values displayed in each figure account for all sources of uncertainty and include the effects of bin-to-bin correlations induced by the systematic uncertainties

The detector calibration and the physics modelling are validated by comparing data with simulated *W*-boson signal and backgrounds for several kinematic distributions that are insensitive to the *W*-boson mass. The comparison is based on a $\chi ^2$ compatibility test, including statistical and systematic uncertainties, and the bin-to-bin correlations induced by the latter. The systematic uncertainty comprises all sources of experimental uncertainty related to the lepton and recoil calibration, and to the background subtraction, as well as sources of modelling uncertainty associated with electroweak corrections, or induced by the helicity fractions of vector-boson production, the vector-boson transverse-momentum distribution, and the PDFs. Comparisons of data and simulation for the $\eta _\ell$, $u_{\mathrm {T}}$, and $u^\ell _\parallel$ distributions, in positively and negatively charged *W*-boson events, are shown in Figs. 19 and 20 for the electron and muon decay channels, respectively.

Data and simulation agree within uncertainties for all distributions, as confirmed by the satisfactory $\chi ^2/$dof values. The effect of the residual discrepancies in the $u_{\mathrm {T}}$ distributions for $W^-\rightarrow \ell {\nu }$, visible at low values in Figs. 19d and 20d, is discussed in Sect. 11.5.

### Data-driven check of the uncertainty in the $p_{\text {T}} ^W$ distribution 

**Fig. 21:**
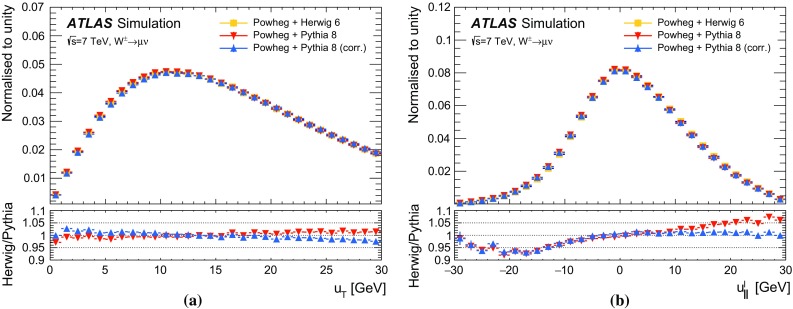
Distributions of **a** $u_{\text {T}}$ and **b** $u_{\parallel }^\ell$ in $W\rightarrow \mu \nu$ events simulated using Powheg+Pythia 8 and Powheg+Herwig 6 after all analysis selection cuts are applied. The Powheg+Pythia 8 distributions are shown before and after correction of the recoil response to that of Powheg+Herwig 6. The lower panels show the ratios of Powheg+Herwig 6 to Powheg+Pythia 8, with and without the recoil response correction in the Powheg+Pythia 8 sample. The discrepancy remaining after recoil corrections reflects the different $p_{\text {T}} ^W$ distributions

The uncertainty in the prediction of the $u^\ell _\parallel$ distribution is dominated by $p_{\text {T}}^W$ distribution uncertainties, especially at negative values of $u^\ell _\parallel$ in the kinematic region corresponding to $u^\ell _\parallel <-15\,\text {GeV}$. This is illustrated in Fig. 21, which compares the recoil distributions in the Powheg+Pythia 8 and Powheg+Herwig 6 samples, before and after the corrections described in Sect. 8.2 (the $p_{\text {T}} ^W$ distribution predicted by Powheg+Pythia 8 is not reweighted to that of Powheg+Herwig 6). As can be seen, the recoil corrections and the different $p_{\text {T}} ^W$ distributions have a comparable effect on the $u_{\text {T}}$ distribution. In contrast, the effect of the recoil corrections is small at negative values of $u^\ell _\parallel$, whereas the difference in the $p_{\text {T}} ^W$ distributions has a large impact in this region.

The sensitivity of the $u^\ell _\parallel$ distribution is exploited to validate the modelling of the $p_{\text {T}} ^W$ distribution by Pythia  8 AZ, and its theory-driven uncertainty, described in Sect. 6.5.2, with a data-driven procedure. The parton-shower factorisation scale $\mu _{\text {F}}$ associated with the $c\bar{q}\rightarrow W$ processes constitutes the main source of uncertainty in the modelling of the $p_{\text {T}} ^W$ distribution. Variations of the $u^\ell _\parallel$ distribution induced by changes in the factorisation scale of the $c\bar{q}\rightarrow W$ processes are parameterised and fitted to the data. The $u^\ell _\parallel$ distribution is predicted for the two boundary values of $\mu _{\text {F}}$, and assumed to vary linearly as a function of $\mu _{\text {F}}$. Variations induced by changes in $\mu _{\text {F}}$ are parameterised using a variable *s* defined in units of the initially allowed range, i.e. values of $s=-1,0,+1$ correspond to half the effect^3^ of changing from $\mu _{\text {F}}=m_V$ to $\mu _{\text {F}}=m_V/2,m_V,2m_V$ respectively. The optimal value of *s* is determined by fitting the fraction of events in the kinematic region $-30<u^\ell _\parallel <-15\,\text {GeV}$. The fit accounts for all experimental and modelling uncertainties affecting the $u^\ell _\parallel$ distribution, and gives a value of $s=-\,0.22 \pm 1.06$. The best-fit value of *s* confirms the good agreement between the the Pythia  8 AZ prediction and the data; its uncertainty is dominated by PDF and recoil-calibration uncertainties, and matches the variation range of $\mu _{\text {F}}$ used for the initial estimation of the $p_{\text {T}} ^W$ distribution uncertainty.

**Fig. 22:**
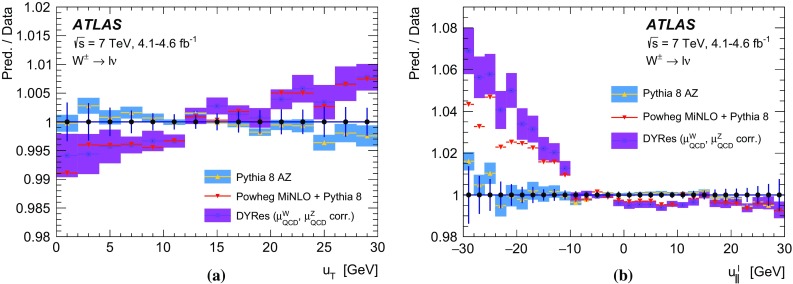
Ratio between the predictions of Pythia 8 AZ, DYRes and Powheg MiNLO+Pythia 8 and the data for the **a** $u_{\text {T}}$ and **b** $u_{\parallel }^\ell$ distributions in $W\rightarrow \ell \nu$ events. The *W*-boson rapidity distribution is reweighted according to the NNLO prediction. The error bars on the data points display the total experimental uncertainty, and the band around the Pythia 8 AZ prediction reflects the uncertainty in the $p_{\text {T}} ^W$ distribution. The uncertainty band around the DYRes prediction assumes that uncertainties induced by variations of the QCD scales $\mu _\text {F}$, $\mu _\text {R}$ and $\mu _\text {Res}$, collectively referred to as $\mu _\text {QCD}$, are fully correlated in *W* and *Z* production

This validation test supports the Pythia 8 AZ prediction of the $p_{\text {T}}^W$ distribution and the theory-driven associated uncertainty estimate. On the other hand, as shown in Fig. 22, the data disagree with the DYRes and Powheg MiNLO+Pythia 8 predictions. The latter are obtained reweighting the initial $p_{\text {T}} ^W$ distribution in Powheg+Pythia 8 according to the product of the $p_{\text {T}} ^Z$ distribution of Pythia  8 AZ, which matches the measurement of Ref. [44], and $R_{W/Z}(p_{\text {T}})$ as predicted by DYRes and Powheg MiNLO+Pythia 8. The uncertainty bands in the DYRes prediction are calculated using variations of the factorisation, renormalisation and resummation scales $\mu _\text {F}$, $\mu _\text {R}$ and $\mu _\text {Res}$ following the procedure described in Ref. [116, 117]. The uncertainty obtained applying correlated scale variations in *W* and *Z* production does not cover the observed difference with the data. The potential effect of using $R_{W/Z}(p_{\text {T}})$ as predicted by DYRes instead of Pythia  8 AZ for the determination of $m_W$ is discussed in Sect. 11.5.

### Results for $m_W$ in the measurement categories

Measurements of $m_W$ are performed using the $p_{\text {T}} ^\ell$ and $m_{\mathrm {T}}$ distributions, separately for positively and negatively charged *W* bosons, in three bins of $|\eta _\ell |$ in the electron decay channel, and in four bins of $|\eta _\ell |$ in the muon decay channel, leading to a total of 28 $m_W$ determinations. In each category, the value of $m_W$ is determined by a $\chi ^2$ minimisation, comparing the $p_{\text {T}} ^\ell$ and $m_{\mathrm {T}}$ distributions in data and simulation for different values of $m_W$. The templates are generated with values of $m_W$ in steps of 1 to $10\,\text {MeV}$ within a range of $\pm \,\, 400\,\text {MeV}$, centred around the reference value used in the Monte Carlo signal samples. The statistical uncertainty is estimated from the half width of the $\chi ^2$ function at the value corresponding to one unit above the minimum. Systematic uncertainties due to physics-modelling corrections, detector-calibration corrections, and background subtraction, are discussed in Sects. 6–8 and 10, respectively.

The lower and upper bounds of the range of the $p_{\text {T}} ^\ell$ distribution used in the fit are varied from 30 to $35\,\text {GeV}$, and from 45 to $50\,\text {GeV}$ respectively, in steps of $1\,\text {GeV}$. For the $m_{\mathrm {T}}$ distribution, the boundaries are varied from 65 to $70\,\text {GeV}$, and from 90 to $100\,\text {GeV}$. The total measurement uncertainty is evaluated for each range, after combining the measurement categories as described in Sect. 11.4 below. The smallest total uncertainty in $m_W$ is found for the fit ranges $32<p_{\text {T}} ^\ell <45\,\text {GeV}$ and $66<m_{\mathrm {T}}<99\,\text {GeV}$. The optimisation is performed before the unblinding of the $m_W$ value and the optimised range is used for all the results described below.

**Fig. 23:**
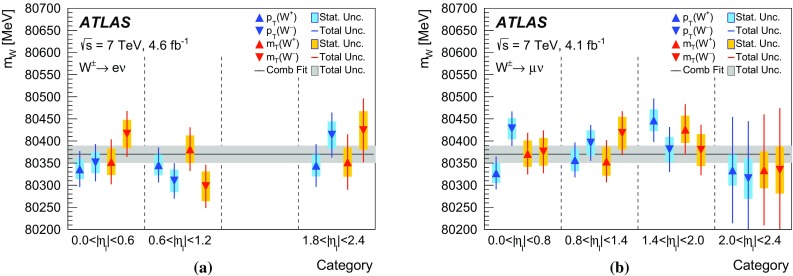
Overview of the $m_W$ measurements in the **a** electron and **b** muon decay channels. Results are shown for the $p_{\text {T}} ^\ell$ and $m_{\mathrm {T}}$ distributions, for $W^+$ and $W^-$ events in the different $|\eta _\ell |$ categories. The coloured bands and solid lines show the statistical and total uncertainties, respectively. The horizontal line and band show the fully combined result and its uncertainty

The final measurement uncertainty is dominated by modelling uncertainties, with typical values in the range 25–$35\,\text {MeV}$ for the various charge and $|\eta _\ell |$ categories. Lepton-calibration uncertainties are the dominant sources of experimental systematic uncertainty for the extraction of $m_W$ from the $p_{\text {T}} ^\ell$ distribution. These uncertainties vary from about $15\,\text {MeV}$ to about $35\,\text {MeV}$ for most measurement categories, except the highest $|\eta |$ bin in the muon channel where the total uncertainty of about $120\,\text {MeV}$ is dominated by the muon momentum linearity uncertainty. The uncertainty in the calibration of the recoil is the largest source of experimental systematic uncertainty for the $m_{\mathrm {T}}$ distribution, with a typical contribution of about $15\,\text {MeV}$ for all categories. The determination of $m_W$ from the $p_{\text {T}} ^\ell$ and $m_{\mathrm {T}}$ distributions in the various categories is summarised in Table 10, including an overview of statistical and systematic uncertainties. The results are also shown in Fig. 23. No significant differences in the values of $m_W$ corresponding to the different decay channels and to the various charge and $|\eta _\ell |$ categories are observed.

**Table 10:** Results of the $m_W$ measurements in the electron and muon decay channels, for positively and negatively charged *W* bosons, in different lepton-$|\eta |$ ranges, using the $m_{\mathrm {T}}$ and $p_{\text {T}} ^\ell$ distributions in the optimised fitting range. The table shows the statistical uncertainties, together with all experimental uncertainties, divided into muon-, electron-, recoil- and background-related uncertainties, and all modelling uncertainties, separately for QCD modelling including scale variations, parton shower and angular coefficients, electroweak corrections, and PDFs. All uncertainties are given in $\,\text {MeV}$

Channel ($m_\mathrm{T}$ fits)	$m_W$ [MeV]	Stat. Unc.	Muon Unc.	Elec. Unc.	Recoil Unc.	Bckg. Unc.	QCD Unc.	EW Unc.	PDF Unc.	Total Unc.
$W^{+}\rightarrow \mu \nu , |\eta |<0.8$	80371.3	29.2	12.4	0.0	15.2	8.1	9.9	3.4	28.4	47.1
$W^{+}\rightarrow \mu \nu , 0.8<|\eta |<1.4$	80354.1	32.1	19.3	0.0	13.0	6.8	9.6	3.4	23.3	47.6
$W^{+}\rightarrow \mu \nu , 1.4<|\eta |<2.0$	80426.3	30.2	35.1	0.0	14.3	7.2	9.3	3.4	27.2	56.9
$W^{+}\rightarrow \mu \nu , 2.0<|\eta |<2.4$	80334.6	40.9	112.4	0.0	14.4	9.0	8.4	3.4	32.8	125.5
$W^{-}\rightarrow \mu \nu , |\eta |<0.8$	80375.5	30.6	11.6	0.0	13.1	8.5	9.5	3.4	30.6	48.5
$W^{-}\rightarrow \mu \nu , 0.8<|\eta |<1.4$	80417.5	36.4	18.5	0.0	12.2	7.7	9.7	3.4	22.2	49.7
$W^{-}\rightarrow \mu \nu , 1.4<|\eta |<2.0$	80379.4	35.6	33.9	0.0	10.5	8.1	9.7	3.4	23.1	56.9
$W^{-}\rightarrow \mu \nu , 2.0<|\eta |<2.4$	80334.2	52.4	123.7	0.0	11.6	10.2	9.9	3.4	34.1	139.9
$W^{+}\rightarrow e\nu , |\eta |<0.6$	80352.9	29.4	0.0	19.5	13.1	15.3	9.9	3.4	28.5	50.8
$W^{+}\rightarrow e\nu , 0.6<|\eta |<1.2$	80381.5	30.4	0.0	21.4	15.1	13.2	9.6	3.4	23.5	49.4
$W^{+}\rightarrow e\nu , 1,8<|\eta |<2.4$	80352.4	32.4	0.0	26.6	16.4	32.8	8.4	3.4	27.3	62.6
$W^{-}\rightarrow e\nu , |\eta |<0.6$	80415.8	31.3	0.0	16.4	11.8	15.5	9.5	3.4	31.3	52.1
$W^{-}\rightarrow e\nu , 0.6<|\eta |<1.2$	80297.5	33.0	0.0	18.7	11.2	12.8	9.7	3.4	23.9	49.0
$W^{-}\rightarrow e\nu , 1.8<|\eta |<2.4$	80423.8	42.8	0.0	33.2	12.8	35.1	9.9	3.4	28.1	72.3
Channel ($p_\mathrm{T}^l$ fits)										
$W^{+}\rightarrow \mu \nu , |\eta |<0.8$	80327.7	22.1	12.2	0.0	2.6	5.1	9.0	6.0	24.7	37.3
$W^{+}\rightarrow \mu \nu , 0.8<|\eta |<1.4$	80357.3	25.1	19.1	0.0	2.5	4.7	8.9	6.0	20.6	39.5
$W^{+}\rightarrow \mu \nu , 1.4<|\eta |<2.0$	80446.9	23.9	33.1	0.0	2.5	4.9	8.2	6.0	25.2	49.3
$W^{+}\rightarrow \mu \nu , 2.0<|\eta |<2.4$	80334.1	34.5	110.1	0.0	2.5	6.4	6.7	6.0	31.8	120.2
$W^{-}\rightarrow \mu \nu , |\eta |<0.8$	80427.8	23.3	11.6	0.0	2.6	5.8	8.1	6.0	26.4	39.0
$W^{-}\rightarrow \mu \nu , 0.8<|\eta |<1.4$	80395.6	27.9	18.3	0.0	2.5	5.6	8.0	6.0	19.8	40.5
$W^{-}\rightarrow \mu \nu , 1.4<|\eta |<2.0$	80380.6	28.1	35.2	0.0	2.6	5.6	8.0	6.0	20.6	50.9
$W^{-}\rightarrow \mu \nu , 2.0<|\eta |<2.4$	80315.2	45.5	116.1	0.0	2.6	7.6	8.3	6.0	32.7	129.6
$W^{+}\rightarrow e\nu , |\eta |<0.6$	80336.5	22.2	0.0	20.1	2.5	6.4	9.0	5.3	24.5	40.7
$W^{+}\rightarrow e\nu , 0.6<|\eta |<1.2$	80345.8	22.8	0.0	21.4	2.6	6.7	8.9	5.3	20.5	39.4
$W^{+}\rightarrow e\nu , 1,8<|\eta |<2.4$	80344.7	24.0	0.0	30.8	2.6	11.9	6.7	5.3	24.1	48.2
$W^{-}\rightarrow e\nu , |\eta |<0.6$	80351.0	23.1	0.0	19.8	2.6	7.2	8.1	5.3	26.6	42.2
$W^{-}\rightarrow e\nu , 0.6<|\eta |<1.2$	80309.8	24.9	0.0	19.7	2.7	7.3	8.0	5.3	20.9	39.9
$W^{-}\rightarrow e\nu , 1.8<|\eta |<2.4$	80413.4	30.1	0.0	30.7	2.7	11.5	8.3	5.3	22.7	51.0

**Fig. 24:**
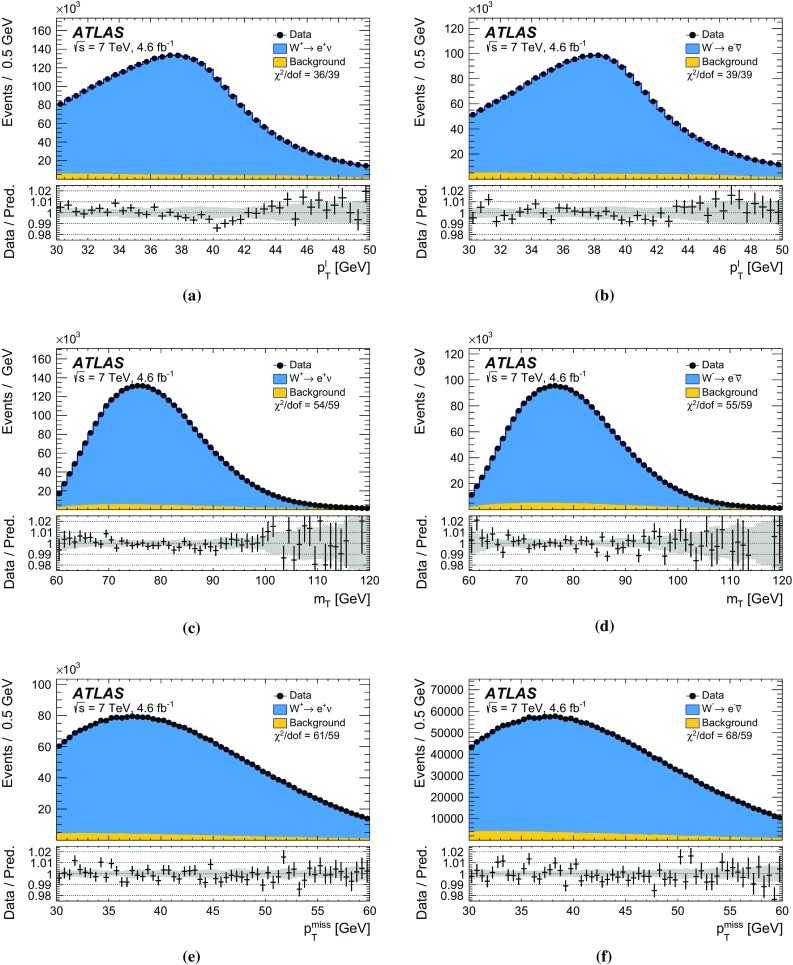
The **a**, **b** $p_{\text {T}} ^\ell$, **c**, **d** $m_{\mathrm {T}}$, and **e**, **f** $p_{\text {T}}^{\text {miss}}$ distributions for **a**, **c**, **e** $W^+$ events and **b**, **d**, **f** $W^-$ events in the electron decay channel. The data are compared to the simulation including signal and background contributions. Detector calibration and physics-modelling corrections are applied to the simulated events. For all simulated distributions, $m_W$ is set according to the overall measurement result. The lower panels show the data-to-prediction ratios, the error bars show the statistical uncertainty, and the band shows the systematic uncertainty of the prediction. The $\chi ^2$ values displayed in each figure account for all sources of uncertainty and include the effects of bin-to-bin correlations induced by the systematic uncertainties

**Fig. 25:**
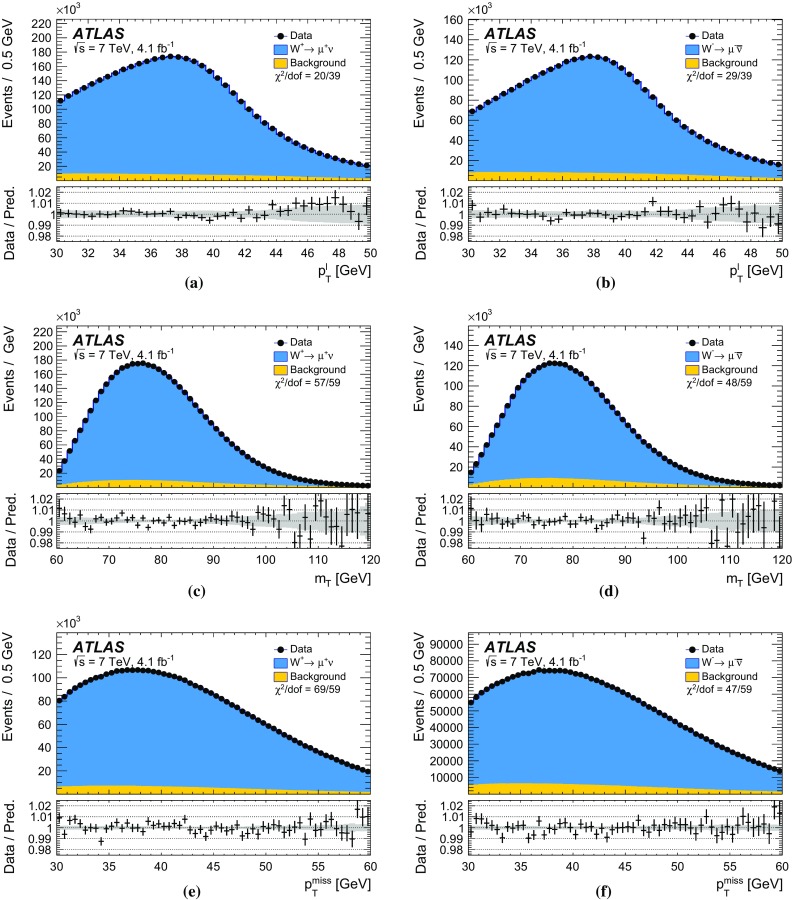
The **a**, **b** $p_{\text {T}} ^\ell$, **c**, **d** $m_{\mathrm {T}}$, and **e**, **f** $p_{\text {T}}^{\text {miss}}$ distributions for **a**, **c**, **e** $W^+$ events and **b**, **d**, **f** $W^-$ events in the muon decay channel. The data are compared to the simulation including signal and background contributions. Detector calibration and physics-modelling corrections are applied to the simulated events. For all simulated distributions, $m_W$ is set according to the overall measurement result. The lower panels show the data-to-prediction ratios, the error bars show the statistical uncertainty, and the band shows the systematic uncertainty of the prediction. The $\chi ^2$ values displayed in each figure account for all sources of uncertainty and include the effects of bin-to-bin correlations induced by the systematic uncertainties

The comparison of data and simulation for kinematic distributions sensitive to the value of $m_W$ provides further validation of the detector calibration and physics modelling. The comparison is performed in all measurement categories. The $\eta$-inclusive $p_{\text {T}} ^\ell$, $m_{\mathrm {T}}$ and $p_{\text {T}}^{\text {miss}}$ distributions for positively and negatively charged *W* bosons are shown in Figs. 24 and 25 for the electron and muon decay channels, respectively. The value of $m_W$ used in the predictions is set to the overall measurement result presented in the next section. The $\chi ^2$ values quantifying the comparison between data and prediction are calculated over the full histogram range and account for all sources of uncertainty. The bin-to-bin correlations induced by the experimental and physics-modelling systematic uncertainties are also accounted for. Overall, satisfactory agreement is observed. The deficit of data visible for $p_{\text {T}} ^\ell \sim 40$–$42\,\text {GeV}$ in the $W^+\rightarrow e\nu$ channel does not strongly affect the mass measurement, as the observed effect differs from that expected from $m_W$ variations. Cross-checks of possible sources of this effect were performed, and its impact on the mass determination was shown to be within the corresponding systematic uncertainties.

### Combination and final results

The measurements of $m_W$ in the various categories are combined accounting for statistical and systematic uncertainties and their correlations. The statistical correlation of the $m_W$ values determined from the $p_{\text {T}} ^\ell$ and $m_{\mathrm {T}}$ distributions is evaluated with the bootstrap method [118], and is approximately 50% for all measurement categories.

The systematic uncertainties have specific correlation patterns across the $m_W$ measurement categories. Muon-momentum and electron-energy calibration uncertainties are uncorrelated between the different decay channels, but largely correlated between the $p_{\text {T}} ^\ell$ and $m_{\mathrm {T}}$ distributions. Recoil-calibration uncertainties are correlated between electron and muon decay channels, and they are small for $p_{\text {T}} ^\ell$ distributions. The PDF-induced uncertainties are largely correlated between electron and muon decay channels, but significantly anti-correlated between positively and negatively charged *W* bosons, as discussed in Sect. 6. Due to the different balance of systematic uncertainties and to the variety of correlation patterns, a significant reduction of the uncertainties in the measurement of $m_W$ is achieved by combining the different decay channels and the charge and $|\eta _\ell |$ categories.

As discussed in Sect. 2, the comparison of the results from the $p_{\text {T}} ^\ell$ and $m_{\mathrm {T}}$ distributions, from the different decay channels, and in the various charge and $|\eta _\ell |$ categories, provides a test of the experimental and physics modelling corrections. Discrepancies between the positively and negatively charged lepton categories, or in the various $|\eta _\ell |$ bins would primarily indicate an insufficient understanding of physics-modelling effects, such as the PDFs and the $p_{\text {T}} ^W$ distribution. Inconsistencies between the electron and muon channels could indicate problems in the calibration of the muon-momentum and electron-energy responses. Significant differences between results from the $p_{\text {T}} ^\ell$ and $m_{\mathrm {T}}$ distributions would point to either problems in the calibration of the recoil, or to an incorrect modelling of the transverse-momentum distribution of the *W* boson. Several measurement combinations are performed, using the best linear unbiased estimate (BLUE) method  [119, 120]. The results of the combinations are verified with the HERAverager program [121], which gives very close results.

Table 11 shows an overview of partial $m_W$ measurement combinations. In the first step, determinations of $m_W$ in the electron and muon decay channels from the $m_{\mathrm {T}}$ distribution are combined separately for the positive- and negative-charge categories, and together for both *W*-boson charges. The results are compatible, and the positively charged, negatively charged, and charge-inclusive combinations yield values of $\chi ^2/$dof corresponding to 2 / 6, 7 / 6, and 11 / 13, respectively. Compatibility of the results is also observed for the corresponding combinations from the $p_{\text {T}} ^\ell$ distribution, with values of $\chi ^2/$dof of 5 / 6, 10 / 6, and 19 / 13, for positively charged, negatively charged, and charge-inclusive combinations, respectively. The $\chi ^2$ compatibility test validates the consistency of the results in the $W\rightarrow e\nu$ and $W\rightarrow \mu \nu$ decay channels. The precision of the determination of $m_W$ from the $m_{\mathrm {T}}$ distribution is slightly worse than the result obtained from the $p_{\text {T}} ^\ell$ distribution, due to the larger uncertainty induced by the recoil calibration. In addition, the impact of PDF- and $p_{\text {T}} ^W$-related uncertainties on the $p_{\text {T}} ^\ell$ fits is limited by the optimisation of the fitting range. In the second step, determinations of $m_W$ from the $p_{\text {T}} ^\ell$ and $m_{\mathrm {T}}$ distributions are combined separately for the electron and the muon decay channels. The results are compatible, with values of $\chi ^2/$dof of 4/5 and 8/5 in the electron channel for the $p_{\text {T}} ^\ell$ and $m_{\mathrm {T}}$ distributions, respectively, and values of 7/7 and 3/7 in the muon channel for the $p_{\text {T}} ^\ell$ and $m_{\mathrm {T}}$ distributions, respectively. The $m_W$ determinations in the electron and in the muon channels agree, further validating the consistency of the electron and muon calibrations. Agreement between the $m_W$ determinations from the $p_{\text {T}} ^\ell$ and $m_{\mathrm {T}}$ distributions supports the calibration of the recoil, and the modelling of the transverse momentum of the *W* boson.

**Table 11:** Results of the $m_W$ measurements for various combinations of categories. The table shows the statistical uncertainties, together with all experimental uncertainties, divided into muon-, electron-, recoil- and background-related uncertainties, and all modelling uncertainties, separately for QCD modelling including scale variations, parton shower and angular coefficients, electroweak corrections, and PDFs. All uncertainties are given in $\,\text {MeV}$

Combined categories	$m_w$ [MeV]	Stat. Unc.	Muon Unc.	Elec. Unc.	Recoil Unc.	Bckg. Unc.	QCD Unc.	EW Unc.	PDF Unc.	Total Unc.	$\chi ^2/$dof
$m_{\mathrm {T}}$, $W^+$, *e*-$\mu$	80370.0	12.3	8.3	6.7	14.5	9.7	9.4	3.4	16.9	30.9	2/6
$m_{\mathrm {T}}$, $W^-$, *e*-$\mu$	80381.1	13.9	8.8	6.6	11.8	10.2	9.7	3.4	16.2	30.5	7/6
$m_{\mathrm {T}}$, $W^\pm$, *e*-$\mu$	80375.7	9.6	7.8	5.5	13.0	8.3	9.6	3.4	10.2	25.1	11/13
$p_{\text {T}} ^\ell$, $W^+$, *e*-$\mu$	80352.0	9.6	6.5	8.4	2.5	5.2	8.3	5.7	14.5	23.5	5/6
$p_{\text {T}} ^\ell$, $W^-$, *e*-$\mu$	80383.4	10.8	7.0	8.1	2.5	6.1	8.1	5.7	13.5	23.6	10/6
$p_{\text {T}} ^\ell$, $W^\pm$, *e*-$\mu$	80369.4	7.2	6.3	6.7	2.5	4.6	8.3	5.7	9.0	18.7	19/13
$p_{\text {T}} ^\ell$, $W^\pm$, *e*	80347.2	9.9	0.0	14.8	2.6	5.7	8.2	5.3	8.9	23.1	4/5
$m_{\mathrm {T}}$, $W^\pm$, *e*	80364.6	13.5	0.0	14.4	13.2	12.8	9.5	3.4	10.2	30.8	8/5
$m_{\mathrm {T}}$-$p_{\text {T}} ^\ell$, $W^+$, *e*	80345.4	11.7	0.0	16.0	3.8	7.4	8.3	5.0	13.7	27.4	1/5
$m_{\mathrm {T}}$-$p_{\text {T}} ^\ell$, $W^-$, *e*	80359.4	12.9	0.0	15.1	3.9	8.5	8.4	4.9	13.4	27.6	8/5
$m_{\mathrm {T}}$-$p_{\text {T}} ^\ell$, $W^\pm$, *e*	80349.8	9.0	0.0	14.7	3.3	6.1	8.3	5.1	9.0	22.9	12/11
$p_{\text {T}} ^\ell$, $W^\pm$, $\mu$	80382.3	10.1	10.7	0.0	2.5	3.9	8.4	6.0	10.7	21.4	7/7
$m_{\mathrm {T}}$, $W^\pm$, $\mu$	80381.5	13.0	11.6	0.0	13.0	6.0	9.6	3.4	11.2	27.2	3/7
$m_{\mathrm {T}}$-$p_{\text {T}} ^\ell$, $W^+$, $\mu$	80364.1	11.4	12.4	0.0	4.0	4.7	8.8	5.4	17.6	27.2	5/7
$m_{\mathrm {T}}$-$p_{\text {T}} ^\ell$, $W^-$, $\mu$	80398.6	12.0	13.0	0.0	4.1	5.7	8.4	5.3	16.8	27.4	3/7
$m_{\mathrm {T}}$-$p_{\text {T}} ^\ell$, $W^\pm$, $\mu$	80382.0	8.6	10.7	0.0	3.7	4.3	8.6	5.4	10.9	21.0	10/15
$m_{\mathrm {T}}$-$p_{\text {T}} ^\ell$, $W^+$, *e*-$\mu$	80352.7	8.9	6.6	8.2	3.1	5.5	8.4	5.4	14.6	23.4	7/13
$m_{\mathrm {T}}$-$p_{\text {T}} ^\ell$, $W^-$, *e*-$\mu$	80383.6	9.7	7.2	7.8	3.3	6.6	8.3	5.3	13.6	23.4	15/13
$m_{\mathrm {T}}$-$p_{\text {T}} ^\ell$, $W^\pm$, *e*-$\mu$	80369.5	6.8	6.6	6.4	2.9	4.5	8.3	5.5	9.2	18.5	29/27

**Fig. 26:**
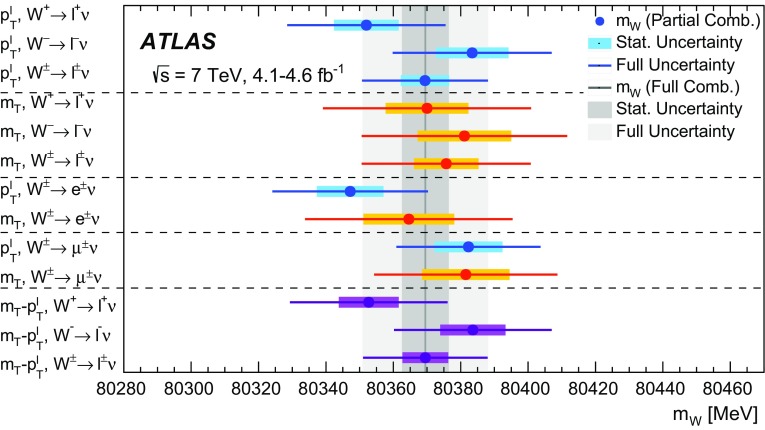
Overview of the $m_W$ determinations from the $p_{\text {T}} ^\ell$ and $m_{\mathrm {T}}$ distributions, and for the combination of the $p_{\text {T}} ^\ell$ and $m_{\mathrm {T}}$ distributions, in the muon and electron decay channels and for $W^+$ and $W^-$ events. The horizontal lines and bands show the statistical and total uncertainties of the individual $m_W$ determinations. The combined result for $m_W$ and its statistical and total uncertainties are also indicated (vertical line and bands)

The results are summarised in Fig. 26. The combination of all the determinations of $m_W$ reported in Table 10 has a value of $\chi ^2/$dof of 29 / 27, and yields a final result of

$$\begin{aligned} m_W= & {} 80369.5 \pm 6.8 (\text {stat.}) \pm 10.6 (\text {exp. syst.}) \\&\qquad \qquad \; \pm 13.6 (\text {mod. syst.}) \,\text {MeV}\\= & {} 80369.5 \pm 18.5 \,\text {MeV}, \end{aligned}$$

where the first uncertainty is statistical, the second corresponds to the experimental systematic uncertainty, and the third to the physics-modelling systematic uncertainty. The latter dominates the total measurement uncertainty, and it itself dominated by strong interaction uncertainties. The experimental systematic uncertainties are dominated by the lepton calibration; backgrounds and the recoil calibration have a smaller impact. In the final combination, the muon decay channel has a weight of 57%, and the $p_{\text {T}} ^\ell$ fit dominates the measurement with a weight of 86%. Finally, the charges contribute similarly with a weight of 52% for $W^{+}$ and of 48% for $W^{-}$.

The result is in agreement with the current world average of $m_W = 80385 \pm 15\,\text {MeV}$ [29], and has a precision comparable to the currently most precise single measurements of the CDF and D0 collaborations [22, 23].

### Additional validation tests

The final combination of $m_W$, presented above, depends only on template fits to the $p_{\text {T}} ^\ell$ and $m_{\mathrm {T}}$ distributions. As a validation test, the value of $m_W$ is determined from the $p_{\text {T}}^{\text {miss}}$ distribution, performing a fit in the range $30<p_{\text {T}}^{\text {miss}} <60\,\text {GeV}$. Consistent results are observed in all measurement categories, leading to combined results of $80364\pm 26$ (stat) MeV and $80367 \pm 23$ (stat) MeV for the electron and muon channels, respectively.

Several additional studies are performed to validate the stability of the $m_W$ measurement. The stability of the result with respect to different pile-up conditions is tested by dividing the event sample into three bins of $\left\langle \mu \right\rangle$, namely [2.5, 6.5], [6.5, 9.5], and [9.5, 16]. In each bin, $m_W$ measurements are performed independently using the $p_{\text {T}} ^\ell$ and $m_{\mathrm {T}}$ distributions. This categorisation also tests the stability of $m_W$ with respect to data-taking periods, as the later data-taking periods have on average more pile-up due to the increasing LHC luminosity.

The calibration of the recoil and the modelling of the $p_{\text {T}} ^W$ distribution are tested by performing $m_W$ fits in two bins of the recoil corresponding to $[0,15]\,\text {GeV}$ and $[15,30]\,\text {GeV}$, and in two regions corresponding to positive and negative values of $u_\parallel ^\ell$. The analysis is also repeated with the $p_{\text {T}}^{\text {miss}}$ requirement removed from the signal selection, leading to a lower recoil modelling uncertainty but a higher multijet background contribution. The stability of the $m_W$ measurements upon removal of this requirement is studied, and consistent results are obtained. All $m_W$ determinations are consistent with the nominal result. An overview of the validation tests is shown in Table 12, where only statistical uncertainties are given. Fitting ranges of $30<p_{\text {T}} ^\ell <50\,\text {GeV}$ and $65<m_{\mathrm {T}}<100\,\text {GeV}$ are used for all these validation tests, to minimise the statistical uncertainty.

**Table 12:** Summary of consistency tests for the determination of $m_W$ in several additional measurement categories. The $\Delta m_W$ values correspond to the difference between the result for each category and the inclusive result for the corresponding observable ($p_{\text {T}} ^\ell$ or $m_{\mathrm {T}}$). The uncertainties correspond to the statistical uncertainty of the fit to the data of each category alone. Fitting ranges of $30<p_{\text {T}} ^\ell <50\,\text {GeV}$ and $65<m_{\mathrm {T}}<100\,\text {GeV}$ are used

Decay channel	$W\rightarrow e\nu$		$W\rightarrow \mu \nu$		Combined	
Kinematic distribution	$p_{\text {T}} ^\ell$	$m_{\mathrm {T}}$	$p_{\text {T}} ^\ell$	$m_{\mathrm {T}}$	$p_{\text {T}} ^\ell$	$m_{\mathrm {T}}$
$$\Delta m_W$$ [MeV]						
$\left\langle \mu \right\rangle$ in [2.5, 6.5]	$8\pm 14$	$14\pm 18$	$-\,21\pm 12$	$0\pm 16$	$-\,9\pm 9\;\,$	$6\pm 12$
$\left\langle \mu \right\rangle$ in [6.5, 9.5]	$-\,6\pm 16$	$6\pm 23$	$12\pm 15$	$-\,8\pm 22$	$4\pm 11$	$-\,1\pm 16$
$\left\langle \mu \right\rangle$ in [9.5, 16]	$-\,1\pm 16$	$3\pm 27$	$25\pm 16$	$35\pm 26$	$12\pm 11$	$20\pm 19$
$u_{\mathrm {T}}$ in $[0,15]\,\text {GeV}$	$0\pm 11$	$-\,8\pm 13$	$5\pm 10$	$8\pm 12$	$3\pm \;\,7$	$-\,1\pm \;\,9$
$u_{\mathrm {T}}$ in $[15,30]\,\text {GeV}$	$10\pm 15$	$0\pm 24$	$-\,4\pm 14$	$-\,18\pm 22$	$2\pm 10$	$-\,10\pm 16$
$u_\parallel ^\ell <0\,\,\text {GeV}$	$8\pm 15$	$20\pm 17$	$3\pm 13$	$-\,1\pm 16$	$5\pm 10$	$9\pm 12$
$u_\parallel ^\ell >0\,\,\text {GeV}$	$-\,9\pm 10$	$1\pm 14$	$-\,12\pm 10$	$10\pm 13$	$-\,11\pm \;\, 7$	$6\pm 10$
No $p_{\text {T}}^{\text {miss}}$-cut	$14\pm \;\,9$	$-\,1\pm 13$	$10\pm \;\,8$	$-\,6\pm 12$	$12\pm \;\,6$	$-\,4\pm \;\,9$

**Fig. 27:**
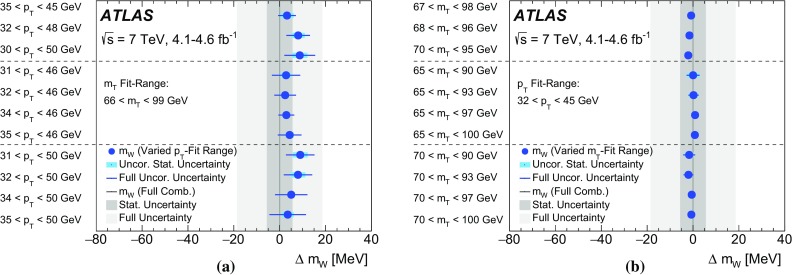
Stability of the combined measurement of $m_W$ with respect to variations of the kinematic ranges of **a** $p_{\text {T}} ^\ell$ and **b** $m_{\mathrm {T}}$ used for the template fits. The optimal $m_{\mathrm {T}}$ range is used for the $p_{\text {T}} ^\ell$ variations, and the optimal $p_{\text {T}} ^\ell$ range is used for the $m_{\mathrm {T}}$ variations. The effect on the result of symmetric variations of the fitting range boundaries, and its dependence on variations of the lower (upper) boundary for two values of the upper (lower) boundary for $p_{\text {T}} ^\ell$ ($m_{\mathrm {T}}$) are shown. The bands and solid lines respectively show the statistical and total uncertainty on the difference with the optimal result

The lower and upper bounds of the range of the $p_{\text {T}} ^\ell$ and $m_{\mathrm {T}}$ distributions are varied as in the optimisation procedure described in Sect. 11.3. The statistical and systematic uncertainties are evaluated for each range, and are only partially correlated between different ranges. Figure 27 shows measured values of $m_W$ for selected ranges of the $p_{\text {T}} ^\ell$ and $m_{\mathrm {T}}$ distributions, where only the uncorrelated statistical and systematic uncertainties with respect to the optimal range are shown. The observed variations are all within two standard deviations of the uncorrelated uncertainties, and small compared to the overall uncertainty of the measurement, which is illustrated by the band on Fig. 27. The largest dependence on the kinematic ranges used for the fits is observed for variations of the upper bound of the $p_{\text {T}} ^\ell$ distribution in the $W^+\rightarrow e\nu$ channel, and is related to the shape of the data-to-prediction ratio for this distribution in the region $40< p_{\text {T}} ^\ell < 42\,\text {GeV}$, as discussed in Sect. 11.3.

The effect of the residual discrepancies in the $u_{\mathrm {T}}$ distributions for $W^-\rightarrow \ell {\nu }$, visible at low values in Figs. 19-(d) and 20-(d), is estimated by adjusting, in turn, the particle-level $p_{\text {T}} ^W$ distribution and the recoil calibration corrections to optimize the agreement between data and simulation. The impact of these variations on the determination of $m_W$ is found to be small compared to the assigned $p_{\text {T}} ^W$ modelling and recoil calibration uncertainties, respectively.

**Table 13:** Results of the $m_{W^+}-m_{W^-}$ measurements in the electron and muon decay channels, and of the combination. The table shows the statistical uncertainties; the experimental uncertainties, divided into muon-, electron-, recoil- and background-uncertainties; and the modelling uncertainties, separately for QCD modelling including scale variations, parton shower and angular coefficients, electroweak corrections, and PDFs. All uncertainties are given in $\,\text {MeV}$

Channel	$m_{W^+}-m_{W^-}$[MeV]	Stat. Unc.	Muon Unc.	Elec. Unc.	Recoil Unc.	Bckg. Unc.	QCD Unc.	EW Unc.	PDF Unc.	Total Unc.
$W\rightarrow e\nu$	$-$ 29.7	17.5	0.0	4.9	0.9	5.4	0.5	0.0	24.1	30.7
$W\rightarrow \mu \nu$	$-$ 28.6	16.3	11.7	0.0	1.1	5.0	0.4	0.0	26.0	33.2
Combined	$-$ 29.2	12.8	3.3	4.1	1.0	4.5	0.4	0.0	23.9	28.0

When assuming $R_{W/Z}(p_{\text {T}})$ as predicted by DYRes, instead of Pythia  8 AZ, to model the $p_{\text {T}} ^W$ distribution, deviations of about 3% appear in the distribution ratios of Figs. 24 and 25. This degrades the quality of the mass fits, and shifts the fitted values of $m_W$ by about $-\,20$ to $-\,90$ MeV, depending on the channels, compared to the results of Table 11. Combining all channels, the shift is about $-\,60$ MeV. Since DYRes does not model the data distributions sensitive to $p_{\text {T}} ^W$, as shown in Fig. 22, these shifts are given for information only and are not used to estimate the uncertainty in $m_W$.

### Measurement of $m_{W^+}-m_{W^-}$

The results presented in the previous sections can be used to derive a measurement of the mass difference between the positively and negatively charged *W* bosons, $m_{W^+}-m_{W^-}$. Starting from the $m_W$ measurement results in the 28 categories described above, 14 measurements of $m_{W^+}-m_{W^-}$ can be constructed by subtraction of the results obtained from the $W^+$ and $W^-$ samples in the same decay channel and $|\eta |$ category. In practice, the $m_W$ values measured in $W^+$ and $W^-$ events are subtracted linearly, as are the effects of systematic uncertainties on these measurements, while the uncertainty contributions of a statistical nature are added in quadrature. Contrarily to the $m_W$ measurement discussed above, no blinding procedure was applied for the measurement of $m_{W^+}-m_{W^-}$.

**Fig. 28:**
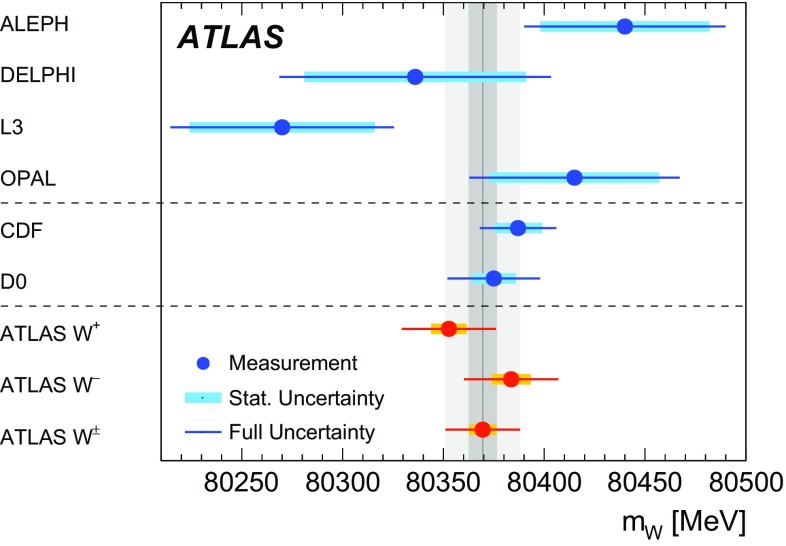
The measured value of $m_W$ is compared to other published results, including measurements from the LEP experiments ALEPH, DELPHI, L3 and OPAL [25–28], and from the Tevatron collider experiments CDF and D0 [22, 23]. The vertical bands show the statistical and total uncertainties of the ATLAS measurement, and the horizontal bands and lines show the statistical and total uncertainties of the other published results. Measured values of $m_W$ for positively and negatively charged *W* bosons are also shown

In this process, uncertainties that are anti-correlated between $W^+$ and $W^-$ and largely cancel for the $m_W$ measurement become dominant when measuring $m_{W^+}-m_{W^-}$. On the physics-modelling side, the fixed-order PDF uncertainty and the parton shower PDF uncertainty give the largest contributions, while other sources of uncertainty only weakly depend on charge and tend to cancel. Among the sources of uncertainty related to lepton calibration, the track sagitta correction dominates in the muon channel, whereas several residual uncertainties contribute in the electron channel. Most lepton and recoil calibration uncertainties tend to cancel. Background systematic uncertainties contribute as the *Z* and multijet background fractions differ in the $W^+$ and $W^-$ channels. The dominant statistical uncertainties arise from the size of the data and Monte Carlo signal samples, and of the control samples used to derive the multijet background.

The $m_{W^+}-m_{W^-}$ measurement results are shown in Table 13 for the electron and muon decay channels, and for the combination. The electron channel measurement combines six categories ($p_{\text {T}} ^\ell$ and $m_{\mathrm {T}}$ fits in three $|\eta _\ell |$ bins), while the muon channel has four $|\eta _\ell |$ bins and eight categories in total. The fully combined result is

$$\begin{aligned} m_{W^+}-m_{W^-}= & {} -29.2 \pm 12.8 (\text {stat.})\\&\pm 7.0 (\text {exp. syst.})\\&\pm 23.9 (\text {mod. syst.})\,\,\text {MeV}\\ \nonumber= & {} -29.2 \pm 28.0 \,\,\text {MeV}, \end{aligned}$$

where the first uncertainty is statistical, the second corresponds to the experimental systematic uncertainty, and the third to the physics-modelling systematic uncertainty.

**Fig. 29:**
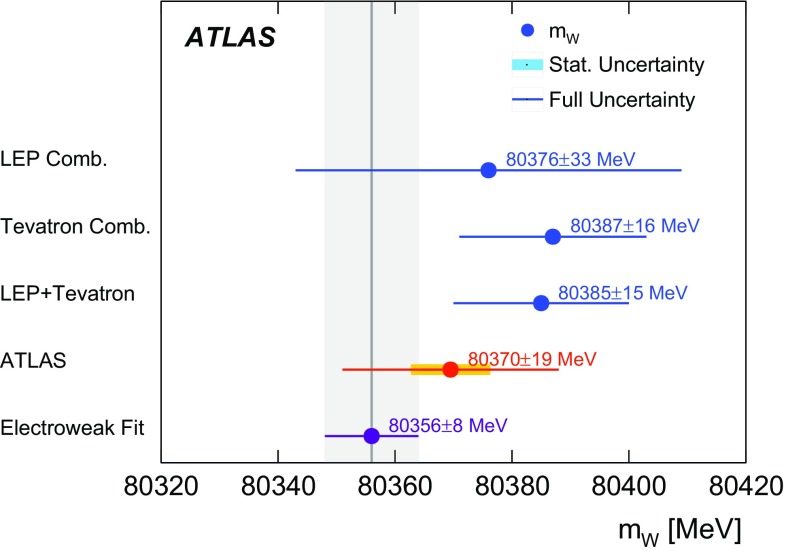
The present measurement of $m_W$ is compared to the SM prediction from the global electroweak fit [16] updated using recent measurements of the top-quark and Higgs-boson masses, $m_t=172.84 \pm 0.70\,\,\text {GeV}$ [122] and $m_{H}=125.09 \pm 0.24 \,\text {GeV}$ [123], and to the combined values of $m_W$ measured at LEP [124] and at the Tevatron collider [24]

**Fig. 30:**
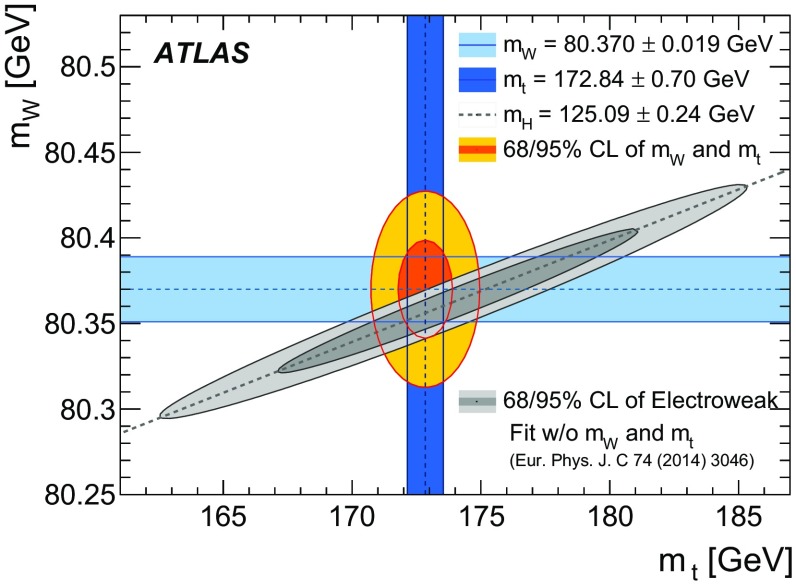
The 68 and 95% confidence-level contours of the $m_W$ and $m_t$ indirect determination from the global electroweak fit [16] are compared to the 68 and 95% confidence-level contours of the ATLAS measurements of the top-quark and *W*-boson masses. The determination from the electroweak fit uses as input the LHC measurement of the Higgs-boson mass, $m_{H}=125.09 \pm 0.24 \,\text {GeV}$ [123]

## Discussion and conclusions 

This paper reports a measurement of the *W*-boson mass with the ATLAS detector, obtained through template fits to the kinematic properties of decay leptons in the electron and muon decay channels. The measurement is based on proton–proton collision data recorded in 2011 at a centre-of-mass energy of $\sqrt{s} = 7\,\text {TeV}$ at the LHC, and corresponding to an integrated luminosity of 4.6 fb$^{-1}$. The measurement relies on a thorough detector calibration based on the study of *Z*-boson events, leading to a precise modelling of the detector response to electrons, muons and the recoil. Templates for the *W*-boson kinematic distributions are obtained from the NLO MC generator Powheg, interfaced to Pythia8 for the parton shower. The signal samples are supplemented with several additional physics-modelling corrections allowing for the inclusion of higher-order QCD and electroweak corrections, and by fits to measured distributions, so that agreement between the data and the model in the kinematic distributions is improved. The *W*-boson mass is obtained from the transverse-momentum distribution of charged leptons and from the transverse-mass distributions, for positively and negatively charged *W* bosons, in the electron and muon decay channels, and in several kinematic categories. The individual measurements of $m_W$ are found to be consistent and their combination yields a value of

$$\begin{aligned} \nonumber m_W= & {} 80370 \pm 7~(\text {stat.}) \pm 11~(\text {exp. syst.})\\&\pm 14~(\text {mod. syst.})\, \,\text {MeV}\\ \nonumber= & {} 80370 \pm 19 \,\,\text {MeV}, \end{aligned}$$

where the first uncertainty is statistical, the second corresponds to the experimental systematic uncertainty, and the third to the physics-modelling systematic uncertainty. A measurement of the $W^+$ and $W^-$ mass difference yields $m_{W^+}-m_{W^-} = -29 \pm 28\,\text {MeV}$.

The *W*-boson mass measurement is compatible with the current world average of $m_W = 80385 \pm 15 \,\text {MeV}$ [29], and similar in precision to the currently leading measurements performed by the CDF and D0 collaborations [22, 23]. An overview of the different $m_W$ measurements is shown in Fig. 28. The compatibility of the measured value of $m_W$ in the context of the global electroweak fit is illustrated in Figs. 29 and 30. Figure 29 compares the present measurement with earlier results, and with the SM prediction updated with regard to Ref. [16] using recent measurements of the top-quark and Higgs boson masses, $m_t=172.84 \pm 0.70\,\,\text {GeV}$ [122] and $m_{H}=125.09 \pm 0.24\,\,\text {GeV}$ [123]. This update gives a numerical value for the SM prediction of $m_W=80356\pm 8\,\text {MeV}$. The corresponding two-dimensional 68 and 95% confidence limits for $m_W$ and $m_t$ are shown in Fig. 30, and compared to the present measurement of $m_W$ and the average of the top-quark mass determinations performed by ATLAS [122].

The determination of the *W*-boson mass from the global fit of the electroweak parameters has an uncertainty of $8\,\text {MeV}$, which sets a natural target for the precision of the experimental measurement of the mass of the *W* boson. The modelling uncertainties, which currently dominate the overall uncertainty of the $m_W$ measurement presented in this paper, need to be reduced in order to fully exploit the larger data samples available at centre-of-mass energies of 8 and $13\,\hbox {TeV}$. Better knowledge of the PDFs, as achievable with the inclusion in PDF fits of recent precise measurements of *W*- and *Z*-boson rapidity cross sections with the ATLAS detector [41], and improved QCD and electroweak predictions for Drell–Yan production, are therefore crucial for future measurements of the *W*-boson mass at the LHC.

## Footnotes

1 Here, lepton refers to the negatively charged lepton from a $W^-$ or *Z* boson, and the neutrino from a $W^+$ boson.

2 Reconstruction-level distributions are obtained from the Powheg+Pythia 8 signal sample by reweighting the particle-level $p_{\text {T}} ^W$ distribution according to the product of the $p_{\text {T}} ^Z$ distribution in Pythia  8 AZ, and of $R_{W/Z}(p_{\text {T}})$ as predicted by Powheg+Pythia 8  AZNLO, DYRes and Powheg MiNLO+Pythia 8.

3 Half the effect is used because only one of the two quarks in the initial state is heavy, as discussed in Sect. 6.5.2.
